# Improved glycemic control with minimal systemic metformin exposure: Effects of Metformin Delayed-Release (Metformin DR) targeting the lower bowel over 16 weeks in a randomized trial in subjects with type 2 diabetes

**DOI:** 10.1371/journal.pone.0203946

**Published:** 2018-09-25

**Authors:** Robert R. Henry, Juan P. Frias, Brandon Walsh, Sharon Skare, John Hemming, Colleen Burns, Thomas A. Bicsak, Alain Baron, Mark Fineman

**Affiliations:** 1 University of California, San Diego, La Jolla, CA, United States of America; 2 National Research Institute, Los Angeles, CA, United States of America; 3 Elcelyx Therapeutics, Inc., San Diego, CA, United States of America; Garvan Institute of Medical Research, AUSTRALIA

## Abstract

**Objective:**

Metformin use is restricted in patients with renal impairment due to potential excess systemic accumulation. This study evaluated the glycemic effects and safety of metformin delayed-release (Metformin DR), which targets metformin delivery to the ileum to leverage its gut-based mechanisms of action while minimizing systemic exposure.

**Research designs and methods:**

Participants (T2DM [HbA1c 7–10.5%], eGFR ≥60 mL/min/1.73m^2^, not taking metformin for ≥2 months) were randomized to QD placebo (PBO); QD Metformin DR 600, 900, 1200, or 1500 mg; or to single-blind BID Metformin immediate-release (IR) 1000 mg. The primary endpoint was change in HbA1c for Metformin DR vs. PBO at 16 weeks in the modified intent-to-treat (mITT) population (≥ 1 post-baseline HbA1c while on study drug), using a mixed-effects repeated measures model.

**Results:**

571 subjects were randomized (56 years, 53% male, 80% white; BMI 32.2±5.5 kg/m^2^; HbA1c 8.6±0.9%; 51% metformin naive); 542 were in the mITT population. Metformin DR 1200 and 1500 mg significantly reduced HbA1c (-0.49±0.13% and -0.62±0.12%, respectively, vs. PBO -0.06±0.13%; p<0.05) and FPG (C_average Weeks 4–16_: -22.3±4.2 mg/dL and -25.1±4.1 mg/dL, respectively vs. -2.5±4.2 mg/dL p<0.05). Metformin IR elicited greater HbA1c improvement (-1.10±0.13%; p<0.01 vs. Placebo and all doses of Metformin DR) but with ~3-fold greater plasma metformin exposure. Normalizing efficacy to systemic exposure, glycemic improvements with Metformin DR were 1.5-fold (HbA1c) and 2.1-fold (FPG) greater than Metformin IR. Adverse events were primarily gastrointestinal but these were less frequent with Metformin DR (<16% incidence) vs. Metformin IR (28%), particularly nausea (1–3% vs 10%).

**Conclusion:**

Metformin DR exhibited greater efficacy per unit plasma exposure than Metformin IR. Future studies will evaluate the effects of Metformin DR in patients with type 2 diabetes and advanced renal disease.

**Trial registration:**

Clinicaltrials.gov NCT02526524.

## Introduction

Metformin is a first-line treatment for T2DM that has been used for over 60 years. Upon ingestion, approximately 50% of metformin is absorbed primarily from the duodenum and jejunum [1–4], and systemically-available metformin is renally excreted unchanged [1]. Metformin elicits concentration-dependent impairment of oxidative phosphorylation, which can increase lactate via both increased production and reduced clearance (conversion of lactate to pyruvate)[5, 6]. Most patients can adequately clear clinically-relevant doses of metformin to avoid substantial systemic accumulation. However, in instances of overdose [7] or when metformin is not adequately cleared, excessive metformin accumulation in the plasma and liver increases the risk of metformin-associated lactic acidosis (MALA), a rare (<0.1/‌1000 patient-years) but often fatal condition [5, 8–11]. Most cases of metformin accumulation are insufficient to cause MALA on their own, but its direct and independent effects on oxidative metabolism renders patients more susceptible to lactic acidosis in the event of an intercurrent illness or injury that also increases lactate production and/or disrupts lactate clearance (e.g., dehydration, sepsis, cirrhosis, hypoperfusion, acute kidney injury)[10–14]. As these intercurrent events are typically not predictable, metformin accumulation in moderate to severe renal impairment should be avoided and concerns over metformin accumulation have led to contraindication in patients with chronic kidney disease (CKD) Stage 4, and recommendation/labeling to use with caution and at reduced dose in CKD Stage 3B [8, 15, 16].

Restriction of metformin use in the CKD 3B/4 population is particularly problematic because most alternative agents that do not increase the risk of hypoglycemia are restricted or have modest efficacy in this population [17–19]. In particular, DPP4is, are less efficacious in the absence of metformin [17] and sodium-glucose co-transporter 2 inhibitors (SGLT2is) are poor to ineffective at glucose lowering in advanced renal disease [18, 20] and may pose a risk of acute renal failure. As such, increased use of hypoglycemia-inducing agents (insulin, sulfonylureas) is common in this population. Treatment with these agents is complicated by the fact that renal disease is an independent risk factor for hypoglycemia through several mechanisms including decreased renal insulin clearance and reduced renal glucose production during counterregulation [21–23]. Furthermore, hypoglycemia is of particular concern in patients with advanced renal disease, as it leads to greater risk of declining renal function, stroke, and excessive mortality [22, 24–26].

While current metformin formulations target systemic delivery of metformin, it is now understood that metformin also accumulates in the intestinal mucosa throughout the length of the gut [3, 4, 27–31], and that much of the glucose-lowering effect of metformin is mediated via actions in the gut [32–36]. Putative gut-based mechanisms include direct and indirect enhanced secretion of glucagon-like peptide-1 (GLP-1) and peptide YY (PYY) from the intestinal L-cell, via alterations in the gut bile acid pool, microbiome products, and effects on hepatic glucose production via intestinal vagal afferents [32, 33, 37, 38]. A delayed-release formulation of metformin (Metformin DR) is being developed to leverage the gut-based mechanisms of action of metformin and address the unmet medical need for metformin use in the advanced CKD population. To that end, Metformin DR is designed to dissolve in the distal small intestine where absorption is poor, thus dramatically limiting plasma exposure while retaining much of the glycemic efficacy observed with current formulations of metformin [34].

This Phase 2 study investigated the glycemic effects of a range of QD Metformin DR doses compared with placebo over 16 weeks in patients with T2DM who were metformin naïve or had discontinued metformin for at least 60 days. A single-blind reference arm of maximally effective (2000 mg) Metformin IR was also included.

## Materials and methods

### Study design

This randomized, Phase 2, parallel-group, multicenter, placebo-controlled study of double-blind placebo or 4 doses of Metformin DR (600, 900, 1200, and 1500 mg), and single-blind Metformin IR (2000 mg), included a 2-week lead-in period and a 16-week treatment period (S1 Fig). During the single-blind lead-in period, subjects received placebo once daily at the beginning of the morning meal (qAM) in order to assess subject compliance prior to randomization. Study participants were then assigned to 1 of 6 treatment arms in equal ratio using a centrally generated computer-based randomization scheme; randomization was stratified and conducted in blocks using screening HbA1c (<8.5% or ≥8.5%). Subjects randomized to Metformin IR were not informed of their treatment, but because Metformin IR was dosed BID study staff were not blinded to the treatment assignment and this treatment arm should be considered a reference arm.

The study consisted of a Screening Visit, a Lead-in Visit, and 7 treatment visits (Day 1 through Week 16). If previously taking metformin, subjects washed out of metformin therapy for 2 months following a preliminary washout screening. Subjects meeting all eligibility criteria initiated the 2-week placebo lead-in period at the Lead-in Visit (Week -2). Randomized study medication was inititated at Day 1 and continued through Week 16. Randomization was conducted centrally via an interactive web response system (IWRS) within each screening HbA1c stratum (<8.5% or ≥8.5%) using a computer-generated, list-based scheme. The study-site pharmacist or other medically qualified personnel contacted IWRS to randomize subjects and receive blinded study medication kit assignments. Subjects randomized to the single-blind (study sites were aware of this treatment assignment, but this information was not actively disclosed to the subjects) Metformin IR treatment arm initiated medication with 1000 mg qAM, and increased dosage to 1000 mg BID from Day 8 through study end. Metformin DR and Placebo were administered qAM with no titration. Visits occurred at Weeks 4, 8, 12, and 16 following an overnight fast; visits at Weeks 2 and 6 occured at least 4 hours after subjects administered the morning dose of study medication for pharmacokinetic (PK) sampling.

Subjects with an FPG or HbA1c value greater than a prespecified threshold (≥270 mg/dL from Week 4 through 8, or ≥240 mg/dL after Week 8; HbA1c increase of >1.5% from baseline; HbA1c >11.0%) had the test repeated within 3 to 7 days. If the results from both tests exceeded the threshold, the subject was monitored and could be rescued with non-metformin antidiabetic therapy according to Investigator judgment.

Metformin DR tablets were produced according to current Good Manufacturing Practices and comprised an immediate-release metformin hydrochloride core overlaid with a proprietary enteric coat to delay disintegration and dissolution until pH 6.5. The Metformin IR treatment was commercially-available Glucophage® (Bristol-Myers Squibb; Princeton, NJ).

The study protocol was conducted in accordance with Good Clinical Practice and approved by the institutional review board/ethics committee of the participating centers. All but three of the study sites conducted the study under the oversight of Copernicus Group Institutional Review Board (Research Triangle Park, NC, USA) with the remaining three operating under their respective local review boards. All participants provided written informed consent prior to enrollment.

### Eligibility criteria

Subjects were males and non-pregnant females, at least 25 years of age, with T2DM (HbA1c 7.0% to 10.5%) and an estimated glomerular filtration rate (eGFR) of ≥60 mL/min/1.73 m^2^ who were not taking metformin for at least 2 months. Stable doses of a concomitant thiazolidinedione, sulfonylurea, dipeptidyl peptidase-4 inhibitor (DPP4i), and alpha glucosidase inhibitor were allowed. Inclusion / Exclusion criteria are listed in the Supplementary Material (S1 File).

### Analysis populations

The ITT Population, used for safety analyses, consists of subjects randomized on Day 1 who received at least one dose of randomized study medication. The mITT Population consists of ITT subjects with at least one post-baseline value for HbA1c that was collected no more than 1 week after discontinuing study medication and prior to taking a new anti-diabetic concomitant medication that could reasonably be expected to influence subsequent glycemic data, and was the primary analysis population for efficacy endpoints. The Evaluable Population included subjects who completed the study without any major protocol deviations and was used in supportive analyses.

### Study endpoints

The primary endpoint was change in HbA1c from baseline at Week 16 in the mITT population. Additional endpoints included changes in HbA1c at timepoints prior to Week 16, change in FPG, fasting plasma metformin concentrations, and percent change in body weight. Change in FPG was analyzed using the pharmacodynamic parameter, C_average_, to provide an integrated value of glucose-lowering throughout the duration of the trial and was calculated for each subject as the area under the curve of the changes in FPG over Weeks 4 to 16 divided by the time on study medication.

Secondary endpoints also included assessments of treatment-emergent adverse events (TEAEs), coded and categorized per the MedDRA coding dictionary.

### Population PK modeling

Metformin exposure (AUC_0-24h_) was estimated from samples obtained in this study using a Population PK model developed to characterize the absorption and disposition of metformin. The population PK model was developed using a dataset that included 5,854 plasma and 762 urine observations from 108 subjects with varying degrees of renal impairment from prior studies of Metformin DR who received orally administered single or multiple doses of metformin given as Metformin IR, Metformin extended-release (XR), or Metformin DR. [39]

### Statistical analysis

The primary analysis of the primary efficacy endpoint (change in HbA1c from baseline at Week 16) used a mixed model with repeated measures (MMRM) that included fixed class effects for treatment, time, treatment-by-time interaction, and baseline HbA1c as a covariate. For the primary analysis, missing data was not imputed in the MMRM model. Dose response analyses were conducted using a general linear model with a linear contrast that accounted for sample size and dose level for each ascending dose [40].

Study power was calculated based on detecting a statistically significant difference for at least one Metformin DR treatment versus Placebo. The assumptions for the calculation included a two-sided α = 0.05, 90% power, a two sample t-test with a common standard deviation of 1.195% and a true HbA1c difference between treatments of -0.6%. Using these assumptions, the number of subjects to be randomized was 92 per treatment in order to get approximately 85 subjects with at least one post-baseline HbA1c value. Supportive analyses were conducted in the mITT and ITT Populations using the last observation carried forward (LOCF) and in the Evaluable Population using observed data. No adjustments for multiplicity were made in testing the primary hypotheses or in testing secondary and additional endpoints.

The secondary and additional endpoints, including change in FPG values, plasma fasting metformin concentrations, and percent change in body weight, were analyzed in the same manner as described for the primary efficacy endpoint. Each model also included the baseline value that corresponds to the particular dependent variable as a covariate. Analyses of dichotomous endpoints employed a logistic regression model adjusting for baseline HbA1c to compare the probability of achieving the dependent variable of interest using similar censoring and imputation methods as for the primary analysis.

A time to event analysis was conducted using the System Organ Class for gastrointestinal TEAEs in the ITT Population. The time to the first event was analyzed using a Cox proportional hazards regression model with treatment as a factor.

All statistical analyses were conducted using SAS® version 9.3.

The full study protocol is provided in the Supplemental Material (S2 File).

## Results

**Fig 1:**
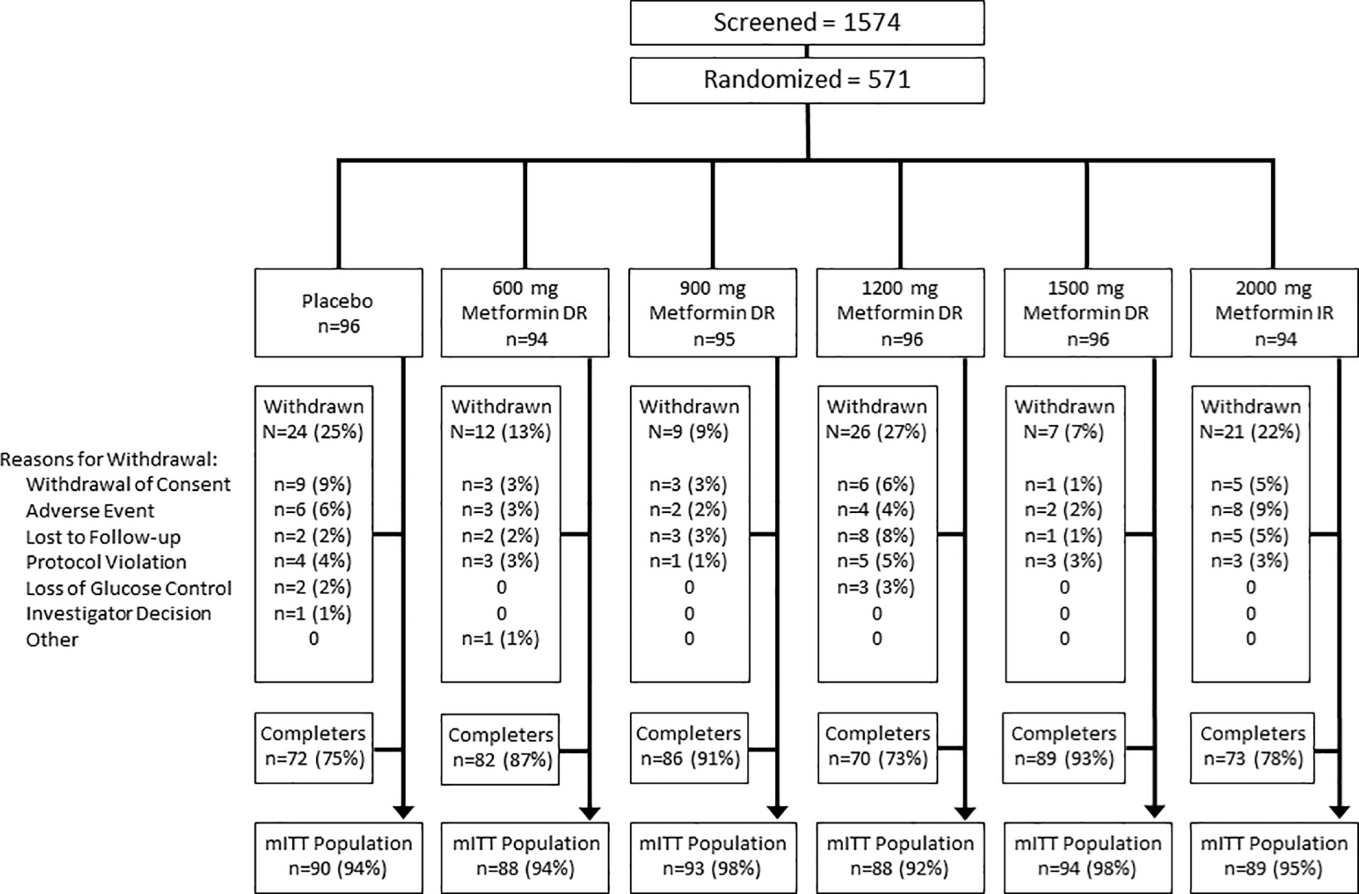
Study flow diagram.

All of the 571 participants who were randomized at 118 US sites were included in the ITT population, 542 subjects were included in the mITT Population, and 403 subjects were included in the Evaluable Population (Fig 1). The first study visit occurred on 02 September 2015 and last study visit occurred on 22 September 2016. Demographic and baseline characteristics were similarly distributed across treatments (Table 1). Most subjects were White (80%), 43% were Hispanic or Latino, and 53% were male. Mean age was 56 years, mean body weight was 91 kg, and mean BMI was 32 kg/m^2^ at baseline. Mean screening HbA1c was 8.6%, and 52% of subjects had an HbA1c ≥8.5%. Mean fasting glucose at baseline was 204.5 mg/dL. Mean duration of T2DM was 7.9 years.

**Table 1:** Subject demographics.

	Placebo (N = 96)	600 mg Met DR (N = 94)	900 mg Met DR (N = 95)	1200 mg Met DR (N = 96)	1500 mg Met DR (N = 96)	2000 mg Met IR (N = 94)	All Subjects (N = 571)
**Age (y)**							
Mean (SD)	57 (11)	56 (10)	55 (10)	55 (11)	55 (9)	57 (11)	56 (11)
**Sex, (%)**							
Female / Male	55 / 45	49 / 51	40 / 60	47 / 53	44 / 56	48 / 52	47 / 53
**Race [1], n (%)**							
American Indian or Alaska Native	0	2 (2)	0	0	0	2 (2)	4 (1)
Asian	5 (5)	4 (4)	6 (6)	3 (3)	3 (3)	2 (2)	23 (4)
Black or African American	17 (18)	14 (15)	7 (7)	23 (24)	19 (20)	8 (9)	88 (15)
Native Hawaiian or Other Pacific Islander	1 (1)	0	0	0	1 (1)	0	2 (<1)
White	72 (75)	76 (81)	82 (86)	70 (73)	73 (76)	83 (88)	456 (80)
Other	1 (1)	0	0	0	0	0	1 (<1)
**Ethnicity, n (%)**							
Hispanic or Latino	37 (39)	36 (38)	44 (46)	41 (43)	42 (44)	45 (48)	245 (43)
**Body Weight (kg)**							
Mean (SD)	86 (17)	94 (20)	89 (18)	93 (21)	95 (22)	89 (21)	91 (20)
**BMI (kg/m**^**2**^**)**							
Mean (SD)	31 (5)	33 (5)	32 (6)	32 (6)	33 (6)	32 (5)	32 (5)
**HbA1c (%)**							
Mean (SD)	8.6 (0.9)	8.6 (0.9)	8.7 (0.8)	8.7 (0.9)	8.6 (0.9)	8.6 (0.9)	8.6 (0.9)
**HbA1c Stratum, n (%)**							
<8.5%	45 (47)	45 (48)	45 (47)	46 (48)	46 (48)	45 (48)	272 (48)
≥8.5%	51 (53)	49 (52)	50 (53)	50 (52)	50 (52)	49 (52)	299 (52)
**FPG Concentration (mg/dL)**							
Mean (SD)	204 (57)	204 (58)	202 (45)	205 (59)	212 (54)	200 (50)	205 (54)
**Duration of T2DM at Screening (y)**							
Mean (SD)	8.3 (7.0)	6.6 (5.2)	8.8 (8.0)	7.3 (6.3)	7.6 (6.0)	8.6 (7.1)	7.9 (6.7)
**eGFR (mL/min/1.73m**^**2**^**)**							
Mean (SD)	96.4 (27.4)	94.4 (19.1)	94.5 (22.8)	96.2 (24.5)	95.9 (22.8)	96.2 (22.4)	95.6 (23.2)
**eGFR Subgroup, n (%)**							
<90 mL/min/1.73m^2^	42 (44)	42 (45)	46 (48)	36 (38)	42 (44)	43 (46)	251 (44)
≥90 mL/min/1.73m^2^	54 (56)	52 (55)	49 (52)	60 (63)	54 (56)	51 (54)	320 (56)
**Metformin Dose Prior to Washout (mg/d)**							
Mean (SD)	1689 (515)	1693 (475)	1698 (523)	1493 (550)	1485 (535)	1660 (472)	1622 (516)

Abbreviations: DR = Delayed-release; eGFR = Estimated glomerular filtration rate; FPG = Fasting plasma glucose; HbA1c = Hemoglobin-specific A1c fraction; IR = Immediate-release; Met = Metformin; T2DM = Type 2 diabetes mellitus.

[1] A subject may contribute more than one race to the summary.

Among randomized subjects, 48.5% washed out of metformin (mean dose of metformin prior to washout: 1,622±516 mg/day [range across treatments of approximately 1500–1700 mg/day]). The majority of subjects who had prior recent metformin use had been using Metformin IR (n = 235; 85%). A total of 218 (38.2%) subjects continued using allowed prior concomitant diabetes medication(s) during the study. The most common prior concomitant diabetes medications were sulfonylureas (34.5%; range across treatments 26.6–41.1%) and DPP4is (7.2%; range across treatments 5.2–10.6%). Other prior concomitant diabetes medications were used by few subjects overall (thiazolidinediones 2.8%, alpha glucosidase inhibitors 0.1%).

### Metformin exposure

**Fig 2:**
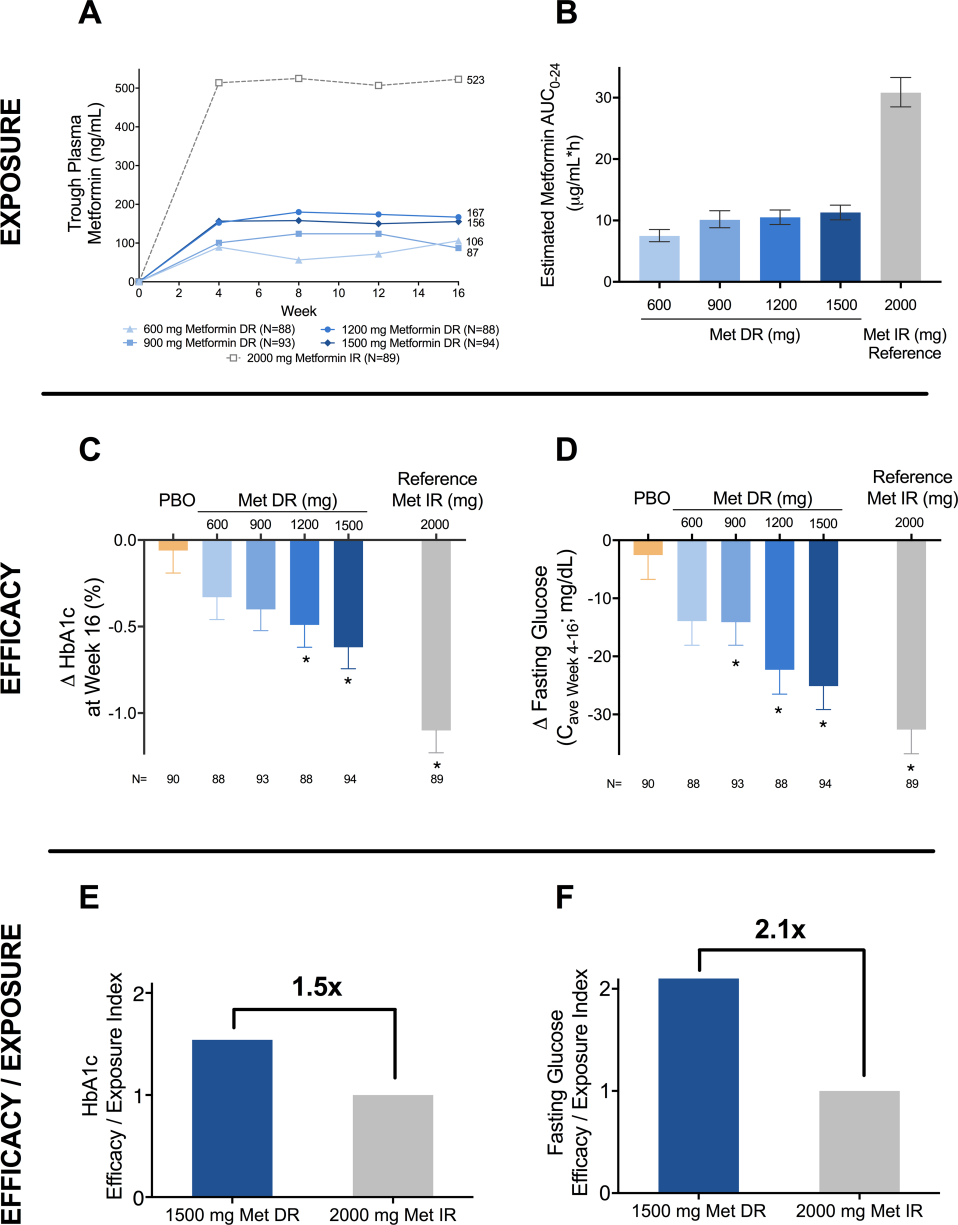
Systemic exposure and glycemic efficacy of metformin DR and metformin IR. Upper Panel: Metformin systemic (plasma) exposure (A) observed at trough (median) and (B) steady state AUC0-24h (geometric mean [95%CI]) estimated from trough and post-dose sampling. Middle Panel: Efficacy presented as (C) HbA1c change at Week 16 (LS mean + SE) and (D) Caverage Week 4–16 change in fasting glucose from baseline (LS mean + SE). Lower Panel: The efficacy/exposure relationship of 1500 mg Metformin DR and 2000 mg Metformin IR represents HbA1c (E) and fasting glucose improvement (F) per unit of systemic metformin exposure. Efficacy/exposure data are HbA1c (LS mean reduction from baseline at Week 16) or reduction in fasting glucose (Caverage Week 4–16) divided by calculated metformin exposure (AUC0-24h); data are normalized to Metformin IR 2000 mg. Data are from the mITT Population (n = 542), with the exception of modeled steady-stated metformin AUC0-24h (ITT population; n = 571). * p<0.05 vs. Placebo.

Plasma metformin samples were obtained at trough (Weeks 4, 8, 12, and 16) and at ≥4 hours post-dose (Weeks 2 and 6). Fig 2A presents median trough plasma metformin concentrations for the mITT Population, which were markedly lower with Metformin DR than with Metformin IR. Population PK modeling (ITT Population) estimated 24-hour exposure (based on fasting and post-dose sampling) to be ≤37% of Metformin IR for all Metformin DR doses (Fig 2B).

### Glycemic control

#### HbA1c

For subjects in the ITT Population requiring washout of previous metformin treatment, the mean±SD HbA1c before washout was 7.9±0.9% (range 7.7±1.0% to 8.1±0.9% across treatment groups), and was 8.7±0.9% at baseline (range: 8.5±0.9% to 9.0±1.0%). For subjects not on metformin at enrollment, the baseline HbA1c was 8.6±0.9% (range 8.4±0.9% to 8.7±0.7%). The mean±SD baseline HbA1c across all treatment arms (ITT or mITT Population) was 8.6±1.0%.

Metformin DR elicited a significant dose response in HbA1c (p = 0.0011), with statistically significant reductions at Week 16 of -0.49±0.13% (1200 mg) and -0.62±0.12% (1500 mg) vs. -0.06±0.13% (Placebo; Fig 2C). Effects were seen as early as Week 4 for 900, 1200, and 1500 mg (S2 Fig). Statistically significant HbA1c reductions vs. Placebo were observed at each time point through Week 16. Metformin DR 900 mg elicited significantly greater reductions than Placebo at all time points except Week 16 (p = 0.06). The 2000 mg Metformin IR reference arm elicited significantly greater reductions in HbA1c from baseline (-1.10±0.13% at Week 16).

Analysis of HbA1c response by subgroups of interest, including prior metformin washout, number of antidiabetes medications, baseline eGFR, ethnicity, and baseline HbA1c stratum (<8.5% or ≥8.5%) were performed in prespecified analyses. Of note, subjects who washed out of previous metformin use exhibited a markedly different profile compared with those that did not wash-out of previous metformin use (S3 Fig). Washout subjects exhibited a more robust apparent dose response with Metformin DR 1500 mg resulting in a change in HbA1c that was 72% of that seen with Metformin IR 2000 mg (i.e., nearly dose proportional), but with maintained low systemic exposure. Analyses of multiple baseline covariates suggest that prior metformin use per se did not influence HbA1c reduction. Specifically, other subject attributes such as increase in HbA1c during washout, baseline HbA1c, and eGFR likely accounted for this observed difference in treatment effect between washout and non-washout subjects.

#### Fasting plasma glucose

Metformin DR exhibited a significant dose response in FPG (p = 0.0022; Fig 2D; S2 Fig), with statistically significant reductions at Week 16 of -20.3±8.1 mg/dL (1200 mg) and -23.4±7.9 mg/dL (1500 mg) vs. -3.1±5.8 mg/dL (Placebo). Compared with Placebo, Metformin DR 1200 mg and 1500 mg achieved statistically greater reductions in FPG from Week 4 through Week 16. As with HbA1c, 900 mg Metformin DR elicited significantly greater FPG reductions than Placebo at earlier timepoints (Weeks 4 and 8), but not later timepoints. At all visits, Metformin IR 2000 mg achieved a significantly greater FPG reduction than Placebo, 600 mg Metformin DR, and 900 mg Metformin DR. Fasting glucose reductions with Metformin IR 2000 mg were not significantly different from Metformin DR 1500 mg from Weeks 8 through 16.

Evaluating the reduction in FPG using C_average Week 4–16_ demonstrates a similar pattern of change. In this analysis, the reduction in FPG (C_average Week 4–16_) was significantly greater than Placebo for 900 mg Metformin DR, 1200 mg Metformin DR, 1500 mg Metformin DR, and 2000 mg Metformin IR (Fig 2D). As observed with change in FPG at Week 16, 2000 mg Metformin IR elicited significantly greater improvement in FPG C_average Week 4–16_ compared with the lower doses of Metformin DR (600 mg and 900 mg); however, the improvement in FPG C_average Week 4–16_ with 1500 mg Metformin DR, although numerically smaller, approached that observed with 2000 mg Metformin IR, eliciting 77% of the effect observed with 2000 mg Metformin IR (there was no statistically significant difference between either 1200 mg or 1500 mg Metformin DR and the 2000 mg Metformin IR dose). Subjects who washed out of metformin also exhibited a more robust dose response in C_average Week 4–16_, with 1500 mg Metformin DR achieving a similar mean reduction to that observed with 2000 mg Metformin IR (-33.9 [5.45] mg/dL vs. -39.5 [5.65] mg/dL, respectively).

#### Efficacy in relation to systemic metformin exposure

The glycemic effects of Metformin DR and Metformin IR described above were observed in the context of markedly different systemic exposure. Therefore, in order to explore the benefit:risk of both formulations, glycemic efficacy was evaluated relative to systemic exposure. When normalized to overall systemic metformin exposure, Metformin DR 1500 mg exhibited 1.5-fold greater HbA1c improvement and 2.1-fold greater fasting glucose improvement than 2000 mg Metformin IR (Fig 2E and 2F).

### Additional endpoints

The percentage of subjects requiring rescue medication was greatest with Placebo (20%), and ranged from 5.7% to 13.6% across Metformin DR treatment arms, and 4.5% with Metformin IR 2000 mg. The use of rescue medication was significantly lower with 2000 mg Metformin IR and 1200 mg Metformin DR compared to Placebo, and there was no significant difference in the use of rescue medication between 2000 mg Metformin IR and Metformin DR doses above 600 mg. Body weight was largely unchanged throughout the trial, with Placebo-corrected LS mean (SE) percent change in body weight from baseline at Week 16 ranging from 0.34% (0.45) with 900 mg Metformin DR to -0.96% (0.46) with 1200 mg Metfromin DR.

### Safety and tolerability

Overall, 46% of subjects reported an adverse event during the study (range 40% to 55% across treatment arms). TEAEs leading to discontinuation of study medication ranged from 2.1% to 7.3% in the Metformin DR treatment arms, compared with 8.5% with Metformin IR treatment and 6.3% with Placebo. The most common TEAEs leading to study medication discontinuation were gastrointestinal in nature (2.3%) and included diarrhea, nausea, and abdominal pain (8, 3, and 2 subjects, respectively, with most events occurring in the Metformin IR treatment arm). Serious TEAEs occurred in 4.2% of Placebo subjects, compared with an incidence ranging from 0% to 4.2% of subjects across Metformin DR treatment arms and 1.1% with Metformin IR; one Placebo subject experienced a TEAE leading to death.

The most common adverse events overall were diarrhea and hyperglycemia (8.6% and 8.4%, respectively); no other events occurred in ≥5% of subjects overall (Table 2). Diarrhea was not reported with Placebo and the greatest incidence was observed with Metformin IR (13.8%). Nausea incidence was 1.0–3.2% across Placebo and all Metformin DR treatment arms, and occurred at 9.6% with Metformin IR. Hyperglycemia incidence tended to be greater with Placebo and lower doses of Metformin DR. The only other events that occurred in ≥5% of subjects in any treatment arm were: worsening of type 2 diabetes (5.2%; Placebo) and upper respiratory tract infection (5.3%; 900 mg Metformin DR).

**Table 2:** Common adverse events.

Treatment-Emergent Adverse Events [1]	Placebo (N = 96) n (%)	600 mg Met DR (N = 94) n (%)	900 mg Met DR (N = 95) n (%)	1200 mg Met DR (N = 96) n (%)	1500 mg Met DR (N = 96) n (%)	2000 mg Met IR (N = 94) n (%)	All Subjects (N = 571) n (%)
Diarrhea	0	7 (7.4)	7 (7.4)	11 (11.5)	11 (11.5)	13 (13.8)	49 (8.6)
Hyperglycemia	10 (10.4)	8 (8.5)	10 (10.5)	7 (7.3)	8 (8.3)	5 (5.3)	48 (8.4)
Nausea	1 (1.0)	3 (3.2)	3 (3.2)	1 (1.0)	3 (3.1)	9 (9.6)	20 (3.5)
Upper respiratory tract infection	2 (2.1)	1 (1.1)	5 (5.3)	1 (1.0)	0	2 (2.1)	11 (1.9)
Worsening Type 2 diabetes mellitus	5 (5.2)	3 (3.2)	1 (1.1)	0	0	0	9 (1.6)

[1] Treatment-emergent adverse events are defined as those occurring at or after the first administration of randomized study medication at Visit 3 (Day 1) through Study Termination, or existing prior to the time of, and worsening after the time of the first administration of randomized study medication

**Fig 3:**
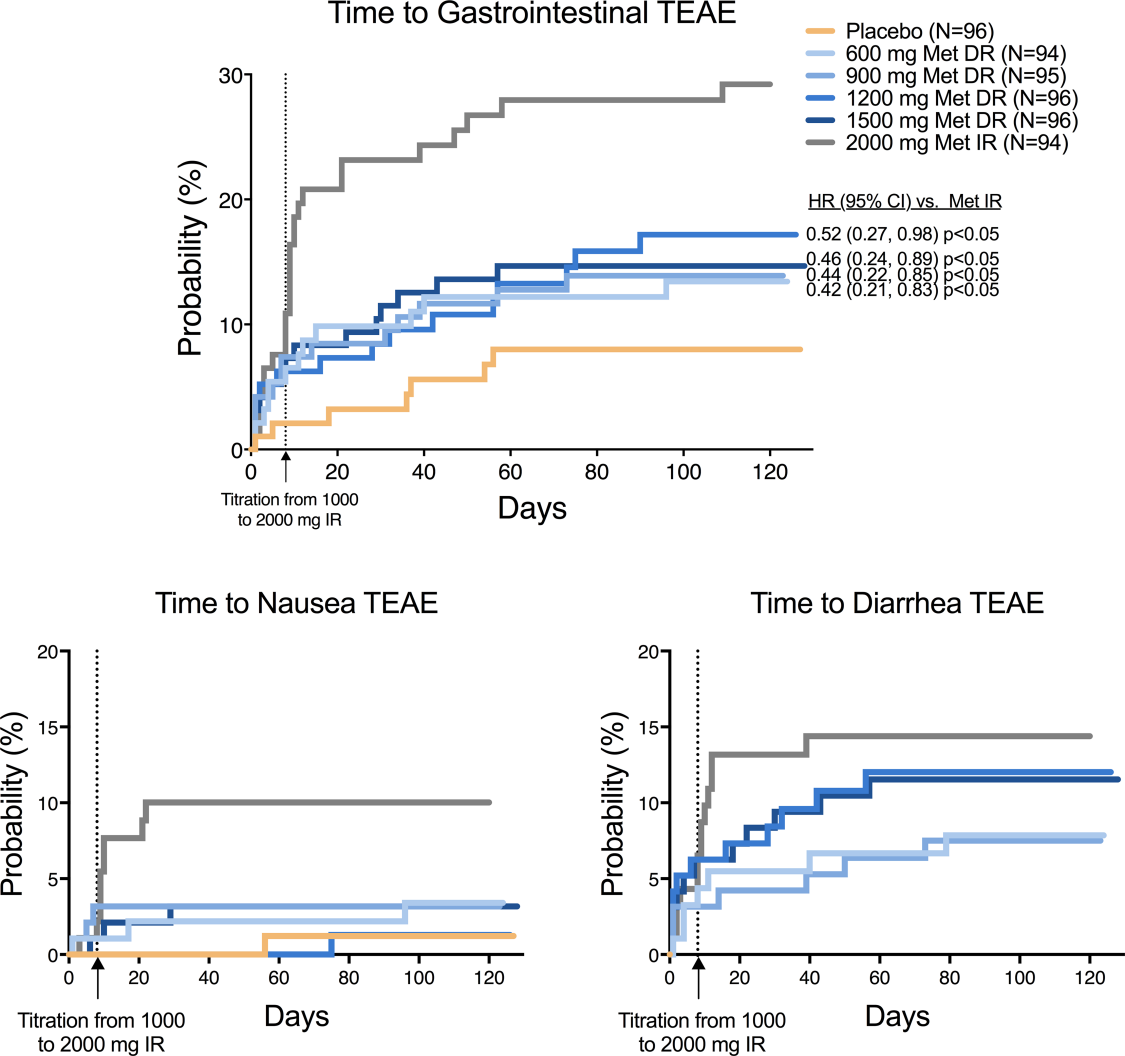
Time to occurrence of gastrointestinal treatment-emergent adverse events. Probability of any gastrointestinal treatment-emergent adverse event (top figure) or nausea/diarrhea events (bottom figures). Abbreviations: CI = Confidence interval; DR = Delayed-release; HR = Hazard ratio; IR = Immediate-release; Met = Metformin; TEAE = Treatment-emergent adverse event. Data are from the ITT Population (n = 571).

The adverse events most commonly associated with metformin use are upper and lower gastrointestinal tract events [8]. Because of the delayed-release profile of Metformin DR, whereby metformin is not released until it reaches the lower gastrointestinal tract, it was of interest to examine gastrointestinal events between treatments. The Metformin IR reference treatment exhibited an earlier time to first gastrointestinal event relative to all Metformin DR arms and placebo (p<0.05 in pairwise comparison), most pronounced at the onset of titration to the full 2000 mg dose at the end of the first week of treatment (Fig 3). No Metformin DR treatment exhibited a significant difference from placebo. Similar patterns were observed with TEAEs of nausea. Although the overall incidence of diarrhea with 1200 mg and 1500 mg Metformin DR, approached that of 2000 mg Metformin IR, diarrhea with Metformin IR exhibited an earlier onset upon titration to the 2000 mg/day dose on Day 8 compared to Metformin DR, which had a more gradual increase in incidence over the first 60 days of treatment.

With the exception of the gastrointestinal adverse events noted above, there were no clinically important differences in adverse event profiles or mean changes in clinical laboratory measures (including blood lacate) or vital signs among treatments.

## Discussion

Owing in part to the deleterious microvascular effects of hyperglycemia and advancing age, a significant proportion (~9% overall; ~19% age ≥ 65 years) of T2DM patients have advanced renal disease (CKD Stages 3B or 4), which results in impaired metformin clearance [41]. Metformin DR is being developed to leverage the gut-based mechanisms of metformin and thereby provide a metformin treatment option with low systemic exposure that may be appropriate for use in T2DM patients with advanced CKD. The delayed release is achieved by use of an pH-dependent enteric coating technology that targets delivery of metformin to the ileum (pH 6.5) where absorption is poor, and density of L-cells and microbiota is high. This study evaluated QD doses of 600 mg, 900 mg, 1200 mg, and 1500 mg Metformin DR, compared with Placebo, over 16 weeks of treatment in subjects with T2DM and eGFR ≥60 mL/min/1.73 m^2^. A single-blind 2000 mg Metformin IR reference arm (1000 mg BID) was also included.

Metformin DR exhibited markedly reduced bioavailability compared with Metformin IR, with trough concentrations reduced by approximately 70% across all doses compared with Metformin IR. Population PK modeling to estimate 24-hour exposure also indicated that all doses of Metformin DR evaluated in this study resulted in approximately 1/3 the exposure of 2000 mg Metformin IR.

Metformin DR exhibited a statistically significant dose response for change from baseline in both HbA1c and FPG with statistically significant improvements in both HbA1c and FPG for 1200 mg and 1500 mg doses compared with Placebo. While the 2000 mg Metformin IR dose elicited significantly greater reductions in HbA1c compared with all doses of Metformin DR, improvement in FPG (C_average Week4-16_) was numerically but not significantly greater with Metformin IR compared to the higher doses of Metformin DR (1200 mg or 1500 mg). Of note, there was no evidence of a plateau for the glycemic effects of Metformin DR, indicating that higher doses of Metformin DR may provide additional glycemic benefit.

The differential effect of 1500 mg Metformin DR compared with 2000 mg Metformin IR on FPG compared with HbA1c implies that Metformin DR at the doses studied can achieve a reduction in FPG approaching that observed with a maximum dose (2000 mg) of Metformin IR, but that Metformin IR may provide additional postprandial glucose control not achieved with Metformin DR. The mechanism responsible for differential postprandial glucose control is unknown, but could result from either systemic or upper bowel metformin exposure as both are reduced or eliminated with the Metformin DR formulation.

When efficacy is considered in the context of markedly lower systemic exposure, Metformin DR shows a favorable profile relative to Metformin IR. The improved efficacy in relation to exposure (Fig 2E and 2F) may be an important consideration supporting use in patients with advanced renal disease, and could be a consideration when evaluating the benefit:risk profile of metformin use in patients at risk of metformin plasma accumulation (and therefore MALA) due to reduced renal function. Importantly, given the greater efficacy per unit of exposure with Metformin DR and the fact that relative bioavailability increases with lower doses of metformin (resulting in proportionally lower gut retention of metformin), reducing current metformin (IR or XR) doses in an attempt to achieve the low systemic exposure of Metformin DR would likely result in a disproportionate loss in efficacy.

Adverse events with all active treatments were primarily gastrointestinal in nature, and were qualitatively similar to those commonly observed with metformin use. Of note, despite being administered QD with no initial dose titration (as was done with Metformin IR), the overall incidence of gastrointestinal events was lower with Metformin DR compared with Metformin IR, particularly for events of nausea. This difference may be related to Metformin DR bypassing the upper gastrointestinal tract prior to dissolution, and could also have been influenced by differences in blinding between Metformin DR and Metformin IR. Events of diarrhea and nausea notably increased upon titration from 1000 mg/day to 2000 mg/day of Metformin IR. However, the single-blind nature of the Metformin IR treatment arm limits the conclusions that can be drawn from these comparisons.

This Phase 2b study evaluated Metformin DR in subjects with normal renal function or mild renal impairment (CKD Stage 1 and 2), in part because Metformin IR (the reference treatment arm) was until recently contraindicated in CKD Stage 3. Recent labeling changes in the US and EU now allow metformin use in CKD Stage 3A. For patients with CKD Stage 3B, initiation of metformin is not allowed per US labeling, and continued use when transitioning to CKD 3B is allowed with caution; in the EU, patients with CKD Stage 3B may use metformin at a suboptimal dose (500 mg BID maximum) which maintains systemic exposure at levels consistent with less advanced renal disease but disproportionately reduces the amount of metformin delivered to the lower bowel and the attendant glucose lowering effects. While a recent uncontrolled, open-label study has confirmed that a reduced metformin dose in CKD 3B (1000 mg total daily dose) and CKD 4 (500 mg total daily dose) results in acceptable systemic metformin concentrations, neither HbA1c nor FPG values changed in either group. Importantly, this open-label study had a small sample size that included both subjects previously taking metformin and subjects who initiated metformin at these low doses, and was not the correct design to assess efficacy [42].

The effects of Metformin DR 1500 mg on both HbA1c and FPG seen in this study were lower than those seen with the Metformin IR 2000 mg reference arm, but consistent with the FPG reductions seen in an earlier 12-week study [34]. It is noteworthy that the effect of Metformin XR 2000 mg used in the earlier study appeared to be attenuated relative to that seen with Metformin IR 2000 mg used in the current study, consistent with modest differences in efficacy for the two formulations reported in product labelling. Thus, while the results from the earlier 12-week study may have suggested that Metformin DR doses ≥1200 mg would perform similarly to Metformin IR 2000 mg, it is possible that differences in efficacy between Metformin IR and Metformin XR may partly explain differences in relative efficacy between the trials.

There were several limitations to this study. First, the open-label reference treatment arm of 2000 mg Metformin IR limits conclusions that can be drawn from comparisons with this arm. This treatment arm was included as a reference arm to contextualize the findings from the placebo and Metformin DR treatment arms. Second, this study evaluated patients with CKD Stage 1 and 2. Future studies in patients with more advanced renal disease, the population for whom this formulation of metformin was developed, will be conducted to confirm these findings in the target population. Finally, the duration of the study (16 weeks), while adequate to assess changes in glycemic control, do not provide an indication of long-term safety/tolerability or durability of treatment effect; longer studies will assess this in the target treatment population.

Metformin DR, with its markedly reduced systemic exposure combined with clinically meaningful glycemic efficacy, exhibits a unique benefit:risk profile not achievable with current metformin formulations. Metformin DR has the potential to be useful in patients for whom metformin is contraindicated or constrained due to concerns of metformin overexposure such as patients with advanced renal impairment.

## Supporting information

S1 Table Consort checklist. Consort 2010 checklist information for a randomized trial.
(DOC)

S1 Fig Study design. Abbreviations: BID = twice daily; DR = delayed-release; IR = immediate-release; Met = metformin; qAM = once daily in the morning.
[1] Subjects washed out of prior metformin therapy if appropriate based on the Investigator’s clinical judgment. Such subjects could qualify for study enrollment at Screening after a 60- to 75-day metformin washout period.
[2] Placebo Lead-in occurred within 2 weeks following Screening. Placebo tablets were identical in size and appearance to Met DR tablets to maintain the treatment blind. The 2-week lead-in period used 600 mg matched placebo tablets (1 tablet qAM).
[3] The Met IR group titrated to a dose of 1000 mg Met IR BID (2000 mg Met IR per day in equal divided doses) on Day 8 from a starting dose of 1000 mg Met IR qAM.
(PDF)

S2 Fig Change in HbA1c and fasting plasma glucose over time. Data are from the mITT Population (n = 542). (LS mean + SE) * = p<0.05 vs. Placebo. DR = Delayed-release; IR = Immediate-release; Met = Metformin.
(PDF)

S3 Fig HbA1c response by metformin washout subgroup. Data are from the mITT Population (n = 542). (LS mean + SE) * = p<0.05 vs. Placebo. DR = Delayed-release; IR = Immediate-release; Met = Metformin.
(PDF)

S1 File Study inclusion/exclusion criteria. (DOCX)

S2 File Study protocol. (PDF)
