# Supplementary material for: Improved glycemic control with minimal systemic metformin exposure: Effects of Metformin Delayed-Release (Metformin DR) targeting the lower bowel over 16 weeks in a randomized trial in subjects with type 2 diabetes
Source: PLoS One. 2018 Sep 25;13(9):e0203946. doi: 10.1371/journal.pone.0203946 (PMC6155522; doi:10.1371/journal.pone.0203946)
Supplement: S1 Fig — Abbreviations: BID = twice daily; DR = delayed-release; IR = immediate-release; Met = metformin; qAM = once daily in the morning. [1] Subjects washed out of prior metformin therapy if appropriate based on the Investigator’s clinical judgment. Such subjects could qualify for study enrollment at Screening after a 60- to 75-day metformin washout period. [2] Placebo Lead-in occurred within 2 weeks following Screening. Placebo tablets were identical in size and appearance to Met DR tablets to maintain the treatment blind. The 2-week lead-in period used 600 mg matched placebo tablets (1 tablet qAM). [3] The Met IR group titrated to a dose of 1000 mg Met IR BID (2000 mg Met IR per day in equal divided doses) on Day 8 from a starting dose of 1000 mg Met IR qAM. (PDF) [file pone.0203946.s002.pdf]

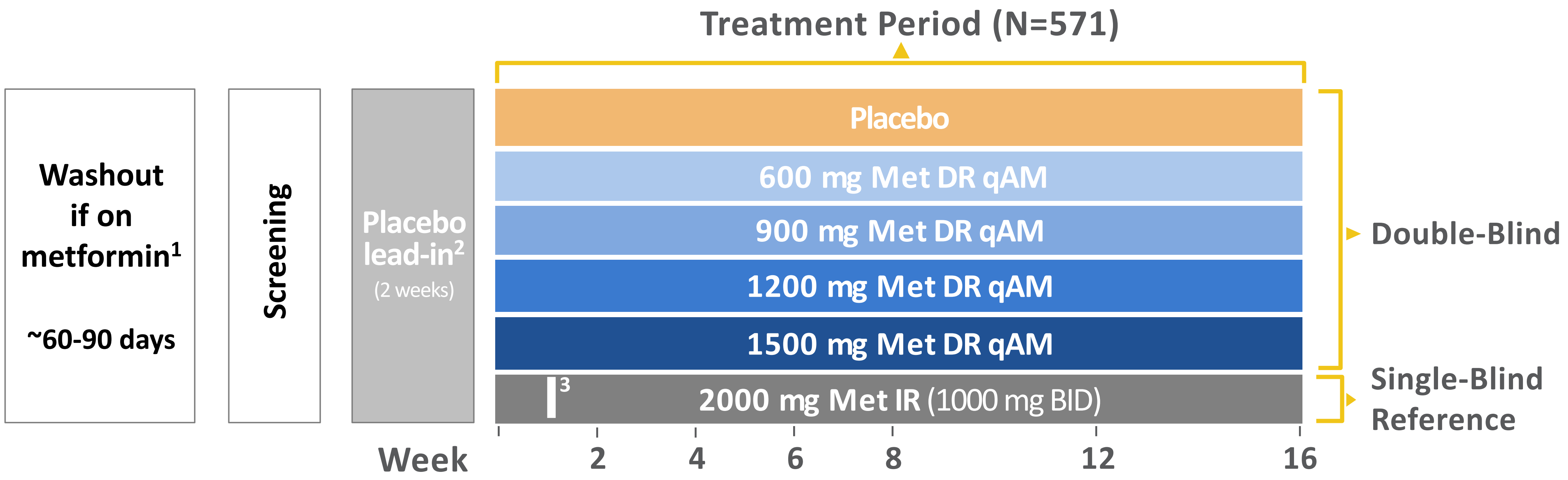
Treatment Period (N=571)
Washout if on metformin[1]
~60-90 days
Screening
Placebo lead-in[2]
(2 weeks)
Placebo
600 mg Met DR qAM
900 mg Met DR qAM
1200 mg Met DR qAM
1500 mg Met DR qAM
2000 mg Met IR (1000 mg BID)
3
Double-Blind
Single-Blind Reference
Week
2
4
6
8
12
16
