# Supplementary material for: Improved glycemic control with minimal systemic metformin exposure: Effects of Metformin Delayed-Release (Metformin DR) targeting the lower bowel over 16 weeks in a randomized trial in subjects with type 2 diabetes
Source: PLoS One. 2018 Sep 25;13(9):e0203946. doi: 10.1371/journal.pone.0203946 (PMC6155522; doi:10.1371/journal.pone.0203946)
Supplement: S2 Fig — Data are from the mITT Population (n = 542). (LS mean + SE) * = p<0.05 vs. Placebo. DR = Delayed-release; IR = Immediate-release; Met = Metformin. (PDF) [file pone.0203946.s003.pdf]

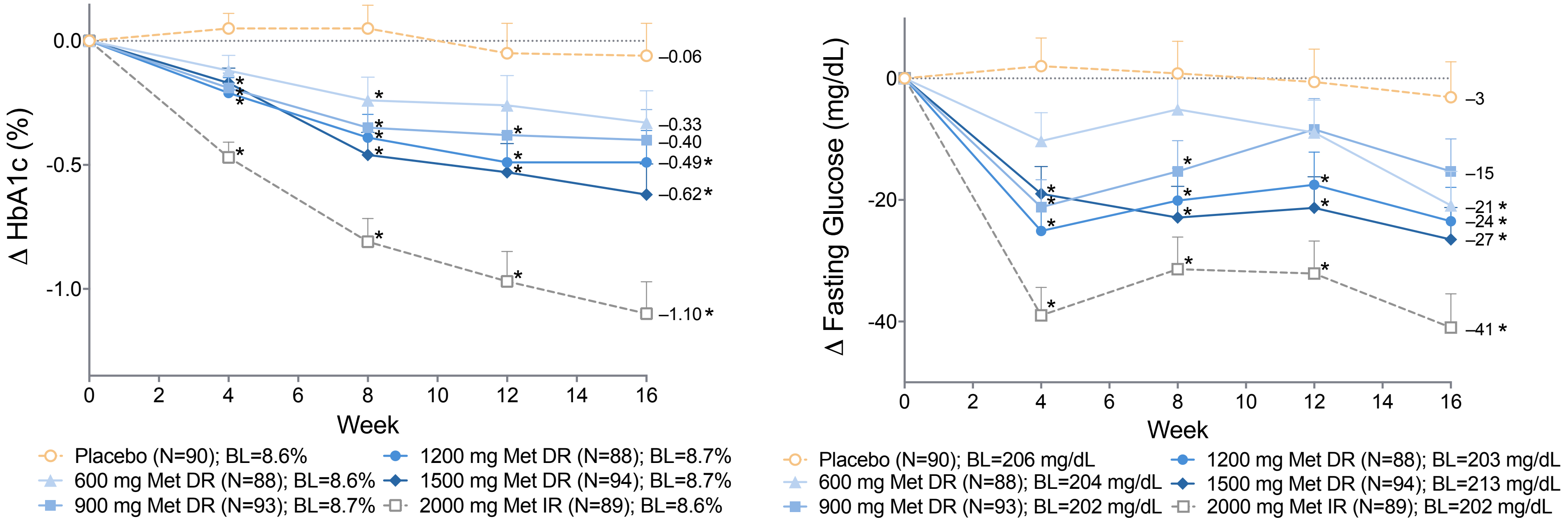

Δ HbA1c (%)
0.0
-0.5
-1.0
Week
0
2
4
6
8
10
12
14
16
–0.06
–0.33
–0.40
–0.49*
–0.62*
–1.10*
Placebo (N=90); BL=8.6%
600 mg Met DR (N=88); BL=8.6%
900 mg Met DR (N=93); BL=8.7%
1200 mg Met DR (N=88); BL=8.7%
1500 mg Met DR (N=94); BL=8.7%
2000 mg Met IR (N=89); BL=8.6%
Δ Fasting Glucose (mg/dL)
0
-20
-40
Week
–3
–15
–21 *
–24 *
–27 *
–41 *
Placebo (N=90); BL=206 mg/dL
600 mg Met DR (N=88); BL=204 mg/dL
900 mg Met DR (N=93); BL=202 mg/dL
1200 mg Met DR (N=88); BL=203 mg/dL
1500 mg Met DR (N=94); BL=213 mg/dL
2000 mg Met IR (N=89); BL=202 mg/dL
