# Supplementary figures and images for: Improved glycemic control with minimal systemic metformin exposure: Effects of Metformin Delayed-Release (Metformin DR) targeting the lower bowel over 16 weeks in a randomized trial in subjects with type 2 diabetes
Source: PLoS One. 2018 Sep 25;13(9):e0203946. doi: 10.1371/journal.pone.0203946 (PMC6155522; doi:10.1371/journal.pone.0203946)

## Non-Washout

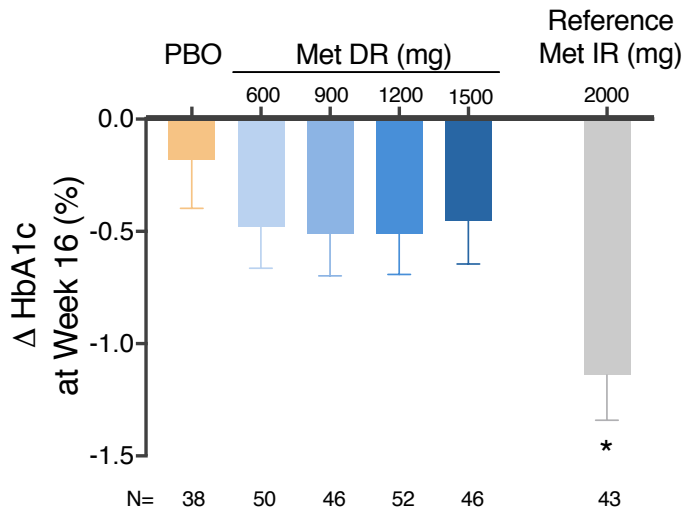

## Washout

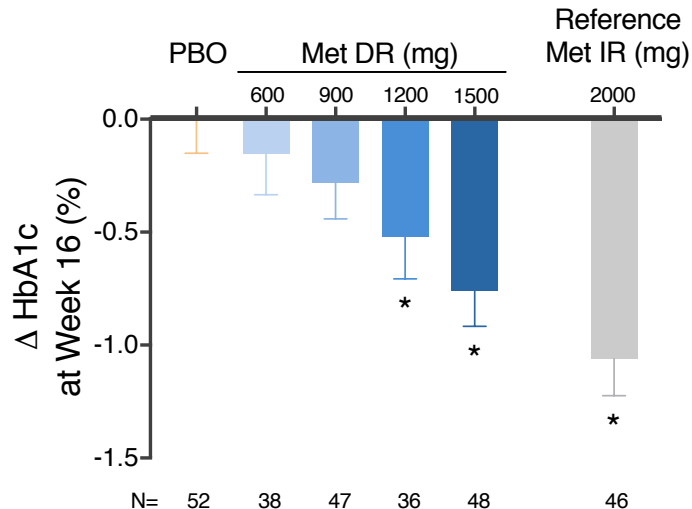

Supplement: S3 Fig — Data are from the mITT Population (n = 542). (LS mean + SE) * = p<0.05 vs. Placebo. DR = Delayed-release; IR = Immediate-release; Met = Metformin. (PDF) [file pone.0203946.s004.pdf]
