# Supplementary material for: Improved glycemic control with minimal systemic metformin exposure: Effects of Metformin Delayed-Release (Metformin DR) targeting the lower bowel over 16 weeks in a randomized trial in subjects with type 2 diabetes
Source: PLoS One. 2018 Sep 25;13(9):e0203946. doi: 10.1371/journal.pone.0203946 (PMC6155522; doi:10.1371/journal.pone.0203946)
Supplement: S1 File — (DOCX) [file pone.0203946.s005.docx]

**Supplementary Data: Inclusion/Exclusion Criteria**

Inclusion Criteria:

Each subject must meet the following criteria to be enrolled in this study.

1. Is at least 25 years old at Visit 1 (Screening).
2. Is male, or is female and meets all of the following criteria:
   1. Not breastfeeding
   2. Negative pregnancy test result (human chorionic gonadotropin, beta subunit [βhCG]) at Visit 1 (Screening) (not applicable to post-menopausal or surgically sterile females)
   3. Surgically sterile, postmenopausal, or if of childbearing potential, must practice and be willing to continue to practice appropriate birth control (defined as a method which results in a low failure rate of less than 1% per year when used consistently and correctly such as implants, injectables, oral contraceptives, some intrauterine contraceptive devices [IUDs], sexual abstinence, tubal ligation, condom with spermicide, or a vasectomized partner) during the entire duration of the study.
3. Body mass index (BMI) 20.0 to 45.0 kg/m^2^ (inclusive) at Visit 1 (Screening).
4. Has a physical examination with no clinically significant abnormalities as judged by the investigator.
5. Has T2DM and an HbA1c of 7.0% to 10.5%, inclusive.
6. Has an eGFR value of ≥60 mL/min/1.73 m^2^ based on the Modification of Diet in Renal Disease (MDRD) equation:
   eGFR (mL/min/1.73 m^2^) = 175 x (S_cr, std_)^-1.154^ x (Age)^-0.203^ x (0.742 if female) x (1.212 if African American).
7. Either is not treated with or has been on a stable treatment regimen with any of the following medications for a minimum of 3 months prior to Visit 1 (Screening):
   1. Thiazolidinedione, sulfonylurea, dipeptidyl peptidase-4 inhibitors, and alpha-glucosidase inhibitors
   2. Hormone replacement therapy (female subjects) and testosterone (male subjects)
   3. Oral contraceptives (female subjects)
   4. Antihypertensive agents
   5. Lipid-lowering agents
   6. Thyroid replacement therapy
   7. Antidepressant agents.
8. Ability to understand and willingness to adhere to protocol requirements.

Exclusion Criteria:

Any subject who meets any of the following criteria is to be excluded from the study.

1. Has a clinically significant medical condition as judged by the investigator that could potentially affect study participation and/or personal well-being, including but not limited to the following conditions:
   1. Hepatic disease
   2. Gastrointestinal disease, including but not limited to:
      1. History or presence of inflammatory bowel disease or other severe gastrointestinal disease, particularly those that may impact gastric emptying, such as gastroparesis and pyloric stenosis
      2. Surgical gastrointestinal procedure that may impact the gut hormonal response to study medication such as gastric bypass surgery or gastric banding surgery
   3. Endocrine disorder (T2DM is allowed)
   4. Cardiovascular disease
   5. Central nervous system diseases
   6. Psychiatric or neurological disorders
   7. Organ transplantation
   8. Chronic or acute infection
   9. Orthostatic hypotension, fainting spells or blackouts
   10. Allergy or hypersensitivity
2. A history of diabetic ketoacidosis or hyperosmolar non-ketotic hyperglycemia within the past year.
3. Prior major surgery of any kind within 6 months of Visit 1 (Screening).
4. A history of >3% weight change within 3 months of Visit 1 (Screening).
5. A clinical laboratory test (clinical chemistry, hematology, or urinalysis) abnormality, other than that related to T2DM, judged by the investigator to be clinically significant.
6. An alanine aminotransferase (ALT) or aspartate aminotransferase (AST) result >2.5 × upper limit of normal (ULN) or a bilirubin result >1.5 × ULN.
7. A physical, psychological, or historical finding that, in the investigator’s opinion, would make the subject unsuitable for the study.
8. Has been treated, is currently being treated, or is expected to require or undergo treatment with any of the following excluded medications:
   1. Metformin within 2 months of Visit 1 (Screening)
   2. Insulin within 2 weeks of Visit 1 (Screening) or for more than 1 week within 3 months of Visit 1 (Screening)
   3. Glucagon-like peptide-1 receptor agonists or sodium-glucose co-transporter 2 inhibitors within 3 months of Visit 1 (Screening)
   4. Drugs known to affect body weight, including prescription medications (e.g., phentermine/topiramate [QSYMIA^®^], orlistat [XENICAL^®^ or ALLI^®^], lorcaserin [BELVIQ^®^], bupropion/naltrexone [CONTRAVE^®^]), and over-the-counter anti‑obesity agents within 3 months of Visit 1 (Screening)
   5. Systemic corticosteroids by oral, intravenous, or intramuscular route; or potent, inhaled, or intrapulmonary steroids known to have a high rate of systemic absorption within 3 months of Visit 1 (Screening)
   6. Planned use of any drug treatment that affects gastric pH (prescription or over-the-counter), such as H2-receptor antagonists and proton pump inhibitors, after Visit 2 (Week -2), or planned chronic use (i.e., more than twice per week) of any antacids after Visit 2 (Week -2).
   7. Cationic drugs that are eliminated by renal tubular secretion (e.g., amiloride, digoxin, morphine, procainamide, flecainide, quinidine, quinine, ranitidine, triamterene, trimethoprim, and vancomycin) within 1 week of Visit 1 (Screening)
   8. Iodinated contrast dye within 1 week prior to Visit 1 (Screening)
   9. Investigational drug within 2 months (or five half-lives of the investigational drug, whichever is greater) of the date of the first dose of randomized study medication
   10. Met DR or double-blind matching placebo for Met DR at any time prior to Visit 1 (Screening)
9. Currently abuses drugs or alcohol or has a history of abuse that in the investigator’s opinion would cause the individual to be noncompliant with study procedures.
10. Had a blood transfusion or experienced significant blood loss (i.e., >500 mL), including loss due to blood donation, within 2 months prior to Visit 1 (Screening), or is planning to donate blood during the study.
11. Has known immune system based allergies or hypersensitivity to any component of study treatment. A history of gastrointestinal intolerance to metformin is not exclusionary.
12. Is employed by Elcelyx Therapeutics, Inc. (that is an employee, contract worker, or designee of the company).
13. Has a fasting plasma glucose value >270 mg/dL at Visit 1 (Screening), Visit 2 (Week ‑2), and an unscheduled visit to be completed within 1 week following Visit 2. The unscheduled visit is to be completed only for subjects with a fasting plasma glucose value >270 mg/dL at Visit 1 and Visit 2.
