# Supplementary material for: Improved glycemic control with minimal systemic metformin exposure: Effects of Metformin Delayed-Release (Metformin DR) targeting the lower bowel over 16 weeks in a randomized trial in subjects with type 2 diabetes
Source: PLoS One. 2018 Sep 25;13(9):e0203946. doi: 10.1371/journal.pone.0203946 (PMC6155522; doi:10.1371/journal.pone.0203946)
Supplement: S2 File — (PDF) [file pone.0203946.s006.pdf]

Clinical Study Protocol LCRM112

A RANDOMIZED, DOUBLE-BLIND, PARALLEL-GROUP, MULTICENTER, PLACEBO-CONTROLLED, DOSE-RANGING STUDY TO EVALUATE THE GLYCEMIC EFFECTS, SAFETY, AND TOLERABILITY OF METFORMIN DELAYED-RELEASE IN SUBJECTS WITH TYPE 2 DIABETES MELLITUS

Original Protocol: 18 May 2015
Amendment 1: 24 July 2015
Amendment 2: 08 October 2015
Amendment 3: 30 November 2015
Amendment 4: 18 January 2016
Amendment 5: 30 March 2016

Development Phase: Phase 2

IND Number: 115,365

Sponsor: Elcelyx Therapeutics, Inc.
11975 El Camino Real, Suite 305
San Diego, CA 92130 USA

Medical Monitor: Phillippa Miranda, MD
Medical Director
Medpace
Telephone: (513) 579-9911 ext. 2275

Confidentiality Statement

This protocol is a confidential communication and is the property of Elcelyx Therapeutics, Inc. (Elcelyx). Acceptance implies an agreement not to disclose the information contained herein that is not otherwise publicly available, except for use by an Institutional Review Board (IRB) for the purpose of obtaining approval to conduct the study. The IRB is requested and expected to maintain confidentiality. This document may not be used or published in whole or in part without the consent of Elcelyx.

Clinical Study Protocol LCRM112

A RANDOMIZED, DOUBLE-BLIND, PARALLEL-GROUP, MULTICENTER, PLACEBO-CONTROLLED, DOSE-RANGING STUDY TO EVALUATE THE GLYCEMIC EFFECTS, SAFETY, AND TOLERABILITY OF METFORMIN DELAYED-RELEASE IN SUBJECTS WITH TYPE 2 DIABETES MELLITUS

Original Protocol: 18 May 2015
Amendment 1: 24 July 2015
Amendment 2: 08 October 2015
Amendment 3: 30 November 2015
Amendment 4: 18 January 2016
Amendment 5: 30 March 2016

Protocol approved by:

[signature] 30-MAR-2016

Mark Fineman, PhD
Chief Scientific Officer
Elcelyx Therapeutics, Inc.
Date

[signature] 30-MAR-2016

Alain Baron, MD
Chief Executive Officer
Elcelyx Therapeutics, Inc.
Date

[signature] 30-MAR-2016

Phillippa Miranda, MD
Medical Director
Medpace
Date

Protocol Amendment 5: Summary of Changes

Date: 30 March 2016

Amendment Purpose: The purpose of this amendment is to broaden the HbA1c criterion for study eligibility to 7.0% to 10.5% and to further clarify the guidance on re-screening of subjects, HbA1c retesting at Visit 2, excluded cationic drugs, and the timing of the morning dose of study medication before Visit 4 and Visit 6. At the time of 50% of subjects randomized, >60% of subjects had a baseline HbA1c of ≥8.5%, whereas per protocol Section 3.1 (Design Description) enrollment is to target ≥30% of subjects with an HbA1c of ≥8.5%. Thus, this change to the HbA1c range will support the specified target as well as more robust subgroup comparisons.

SECTION	AMENDMENT 4 TEXT	AMENDMENT 5 TEXT
Summary – Study Population<br>Section 4.1 – Population to be Studied	Approximately 552 males and non-pregnant females, at least 25 years of age, with T2DM (HbA1c 7.5% to 10.5%) and an estimated glomerular filtration rate (eGFR) of ≥60 mL/min/1.73 m^2 who are not taking metformin for at least 2 months prior to Screening (Visit 1) are to be randomized in this study.	Approximately 552 males and non-pregnant females, at least 25 years of age, with T2DM (HbA1c 7.0% to 10.5%) and an estimated glomerular filtration rate (eGFR) of ≥60 mL/min/1.73 m^2 who are not taking metformin for at least 2 months prior to Screening (Visit 1) are to be randomized in this study.
Summary – Study Population<br>Section 4.1 – Population to be Studied<br>Section 6.1.1 – Visit WS: Washout Screening Procedures	As subjects are required to have HbA1c 7.5 to 10.5% (inclusive) at Visit 1 and Visit 2, it is recommended (but not required) that subjects have HbA1c 7.0 to 9.5% (inclusive) prior to initiation of the wash out period.	It is recommended (but not required) that subjects have HbA1c 7.0 to 9.5% (inclusive) prior to initiation of the wash out period.
Summary – Study Methods	• For Visit 4 (Week 2) and Visit 6 (Week 6):<br>o Subjects are to administer their morning dose of study medication with food at least 4 hours before coming to the clinic	• For Visit 4 (Week 2) and Visit 6 (Week 6):<br>o Subjects are to administer their morning dose of study medication with food at least 4 hours before the blood collection at these visits and on the same day as these visits
Section 4.2 – Inclusion Criteria	Inclusion Criterion 5. Has T2DM and an HbA1c of 7.5% to 10.5%, inclusive.	Inclusion Criterion 5. Has T2DM and an HbA1c of 7.0% to 10.5%, inclusive.
Section 4.3 – Exclusion Criteria	Exclusion criterion 8.g. Cationic drugs that are eliminated by renal tubular secretion (e.g., amiloride, digoxin, morphine, procainamide, quinidine, quinine, ranitidine, triamterene, trimethoprim, and vancomycin) within 1 week of Visit 1 (Screening)	Exclusion criterion 8.g. Cationic drugs that are eliminated by renal tubular secretion (e.g., amiloride, digoxin, morphine, procainamide, flecainide, quinidine, quinine, ranitidine, triamterene, trimethoprim, and vancomycin) within 1 week of Visit 1 (Screening)
Section 4.4 – Subject Restrictions	For Visit 4 (Week 2) and Visit 6 (Week 6):	For Visit 4 (Week 2) and Visit 6 (Week 6):

SECTION	AMENDMENT 4 TEXT	AMENDMENT 5 TEXT
	Administer the morning dose of study medication with food at least 4 hours before coming to the clinic	Administer the morning dose of study medication with food at least 4 hours before the blood collection at these visits and on the same day as these visits.
Section 6.1.2 – Visit 1: Screening Procedures	*Not applicable*	Individuals who do not meet study requirements outlined in the inclusion and exclusion criteria (Sections 4.2 and 4.3) may re-screen for the study with a new screening number after 2 weeks if appropriate based on the investigator's clinical judgment.
Section 6.1.3 – Visit 2 (Week -2): Enrollment, Lead-in Period	If the results of any HbA1c test at Visit 2 fails eligibility thresholds outlined in inclusion criterion 5 (HbA1c of 7.5% to 10.5% inclusive) then both of the criteria below must be met for the subject to continue in the study.<br>• The value increased or decreased by ≤ 0.3% relative to a qualifying Visit 1 value, and<br>• The value is assessed as not a clinically significant change from the Visit 1 value by the investigator in consultation with the medical monitor (or designees)	If the result of the HbA1c test at Visit 2 fails eligibility thresholds outlined in inclusion criterion 5 (HbA1c of 7.0% to 10.5% inclusive), individuals may re-qualify for randomization by having the test repeated once at an unscheduled visit within 7 days following Visit 2 if appropriate based on the investigator's clinical judgment in consultation with the medical monitor (or designees). The repeat HbA1c value must be 7.0% to 10.5% inclusive for the subject to continue in the study.
Section 6.1.6 – Visit 4 (Week 2) and Visit 6 (Week 6): Randomized Treatment Period	Subjects are to administer their morning dose of study medication with food at least 4 hours before coming to the clinic.	Subjects are to administer their morning dose of study medication with food at least 4 hours before the blood collection at these visits and on the same day as these visits.
Page 2 – Sponsor Signature Page	Mark Fineman, PhD<br>Senior Vice President, Research and Development<br>Elcelyx Therapeutics, Inc.	Mark Fineman, PhD<br>Chief Scientific Officer<br>Elcelyx Therapeutics, Inc.

PROTOCOL SUMMARY

STUDY TITLE: A RANDOMIZED, DOUBLE-BLIND, PARALLEL-GROUP, MULTICENTER, PLACEBO-CONTROLLED, DOSE-RANGING STUDY TO EVALUATE THE GLYCEMIC EFFECTS, SAFETY, AND TOLERABILITY OF METFORMIN DELAYED-RELEASE IN SUBJECTS WITH TYPE 2 DIABETES MELLITUS

STUDY PHASE: Phase 2

PRIMARY OBJECTIVE

The primary objective of this study is to:

- Assess the effect of metformin delayed-release (Met DR) compared to placebo on the change in hemoglobin-specific A1c fraction (HbA1c) at Week 16 of treatment

SECONDARY OBJECTIVES

The secondary objectives of this study are to:

- Assess the effect of Met DR compared to placebo at Week 16 of treatment on the following:
  - Change in fasting plasma glucose concentrations
  - Proportion of subjects achieving HbA1c ≤7%
- Compare the effects of Met DR and metformin immediate-release (Met IR) at Week 16 of treatment on the following:
  - Change in HbA1c
  - Change in fasting plasma glucose concentrations
  - Proportion of subjects achieving HbA1c ≤7%
- Assess the safety and tolerability of Met DR over the 16-week treatment period

STUDY DESIGN

- LCRM112 is a Phase 2, randomized, double-blind, parallel-group, multicenter, placebo-controlled, 7-arm, dose-ranging study that will be conducted in approximately 552 subjects with type 2 diabetes mellitus (T2DM).
- Enrollment will target ≥30% of subjects with an HbA1c of ≥8.5%.
- The study will include a 2-week single-blind lead-in period and a 16-week double-blind treatment period.
- During the lead-in period, subjects will be enrolled and will receive placebo once daily in the morning (qAM) (1× 600 mg matched placebo tablet) at the beginning of the morning meal.
- During the treatment period, subjects will be randomly assigned to 1 of 7 treatment arms (Groups A, B, C, D, E, F, and G) in the ratio of 1:1:2:2:2:2:2, respectively. There will be 2 placebo arms (Groups A and B), 4 Met DR arms (Groups C, D, E, and F) and 1 Met IR arm (Group G). Groups A, B, C, D, E, and F will be double-blinded. Group G will be single-blinded.
- Randomization will be stratified by screening HbA1c (<8.5% or ≥8.5%).

Randomized Treatment Arms and Dose Regimens (Protocol LCRM112)

Treatment Arm	N	Morning Dose Regimen [1]	Evening Dose Regimen [1]
A (Placebo)	46	2× 600 mg matched placebo	NA
B (Placebo)	46	1× 600 mg matched placebo<br>1× 900 mg matched placebo	NA
C (600 mg Met DR qAM)	92	1× 600 mg Met DR<br>1× 600 mg matched placebo	NA
D (900 mg Met DR qAM)	92	1× 600 mg matched placebo<br>1× 900 mg Met DR	NA
E (1200 mg Met DR qAM)	92	2× 600 mg Met DR	NA
F (1500 mg Met DR qAM)	92	1× 600 mg Met DR<br>1× 900 mg Met DR	NA
G (2000 mg Met IR)	92	1× 1000 mg Met IR	1× 1000 mg Met IR

Abbreviations: BID=twice daily; qAM=once daily in the morning; NA=not applicable

[1] Number of tablets × tablet dose strength.

- A detailed schedule of study procedures by visit is presented in Appendix 5. The overall study design is shown in the following figure:

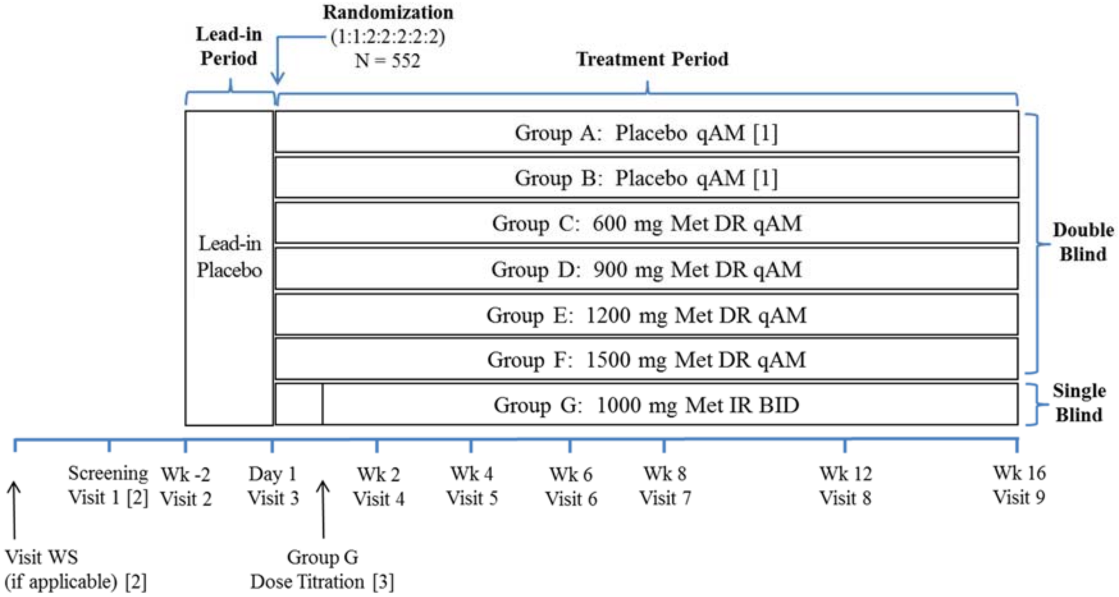

Abbreviations: BID=twice daily; qAM=once daily in the morning

[1] Placebo tablets will be identical in size and appearance to Met DR tablets to maintain the treatment blind. The 2-week lead-in period will use 600 mg matched placebo tablets (1 tablet qAM).

[2] Subjects may wash out of prior metformin therapy after initiation of screening procedures at Visit WS (Washout Screening) before Visit 1 (Screening) if appropriate based on the investigator's clinical judgment. Such subjects may qualify for study enrollment at Visit 1 (Screening) after a 60- to 75-day metformin wash out period. Visit 2 (Week -2) will occur within 2 weeks following Visit 1 (Screening).

[3] The Met IR group will titrate to a dose of 1000 mg Met IR BID (2000 mg Met IR per day in equal divided doses) on Day 8 from a starting dose of 1000 mg Met IR qAM.

VISIT STRUCTURE

- The study will consist of a screening visit (Visit 1), a lead-in visit (Visit 2), and 7 treatment visits (Visits 3 through 9).
- Subjects may wash out of prior metformin therapy after initiation of screening procedures at Visit WS (Washout Screening) before Visit 1 (Screening), if appropriate based on the investigator's clinical judgment. Such subjects may qualify for study enrollment at Visit 1 (Screening) after a 60- to 75-day metformin wash out period.
- Visit 1 procedures may be conducted over >1 day.
- Visit 2 (Week -2) will occur within 2 weeks following Visit 1 (Screening).
- Visit 3 (Day 1) will occur 2 weeks (±3 days) following Visit 2 (Week -2). All visits subsequent to Visit 3 will occur at 2- or 4-week intervals (±3 days relative to Visit 3 [Day 1]).
- Visits 2, 3, 5, 7, 8, and 9 (Week -2, Day 1, and Weeks 4, 8, 12, and 16) are to occur between 0600 and 1000 hours if possible.
- Visits 4 and 6 (Weeks 2 and 6) are to occur at least 4 hours after subjects administer the morning dose of study medication and before subjects who are randomized to single-blind Group G administer the evening dose of study medication.

STUDY DURATION

The total study duration is approximately 18 to 32 weeks depending on the number of days between Visit WS (if applicable), Visit 1, and Visit 2. The study will include a screening period of up to 3 months, a 2-week lead-in period, and a 16-week treatment period.

STUDY POPULATION

Approximately 552 males and non-pregnant females, at least 25 years of age, with T2DM (HbA1c 7.0% to 10.5%) and an estimated glomerular filtration rate (eGFR) of ≥60 mL/min/1.73 m^2 who are not taking metformin for at least 2 months prior to Screening (Visit 1) are to be randomized in this study. Subjects may wash out of prior metformin therapy after initiation of screening procedures at Visit WS (Washout Screening) if appropriate based on the investigator's clinical judgment. It is recommended (but not required) that subjects have HbA1c 7.0 to 9.5% (inclusive) prior to initiation of the wash out period.

STUDY MEDICATION

Study LCRM112 will evaluate the following study medications.

Study medication for the lead-in period:

- Placebo (EFP0084): placebo-matched tablets for EFB0082

Study medications for the treatment period:

- Placebo (EFP0084): placebo-matched tablets for EFB0082
- Placebo (EFP0085): placebo-matched tablets for EFB0083
- Met DR (EFB0082): 600 mg metformin hydrochloride (HCl) delayed-release tablets
- Met DR (EFB0083): 900 mg metformin HCl delayed-release tablets
- Met IR (Glucophage®): 1000 mg metformin HCl immediate-release tablets

Study medications will be supplied in blister packs, should be stored as indicated on the label, and should be used only as directed by study-site personnel.

STUDY METHODS

- The schedule and timing of study procedures by visit are presented in Appendix 5.
- At Visit 2 (Week -2), subjects will complete eligibility evaluations, and those who meet all eligibility requirements will be enrolled in the study and will initiate treatment with lead-in study medication.
- For Visit 3 (Day 1), subjects who complete the lead-in period and demonstrate adequate compliance with protocol procedures as determined by the investigator will be randomly assigned to a treatment arm; will complete baseline efficacy, pharmacokinetic, and safety assessments; and initiate treatment with randomized study medication.
- For Visits 2, 3, 5, 7, 8, and 9 (Week -2, Day 1 and Weeks 4, 8, 12, and 16):
    - Subjects are to arrive at the clinic after fasting (no food or drink except water) overnight for at least 10 hours
    - Subjects are to bring their study medication with them to the clinic (except for Visit 2), and wait to take their morning dose after the fasting blood draws (except for Visit 9; study medication will not be administered on the day of this visit)
    - Subjects are to bring their morning dose of approved antidiabetic medications with them to the clinic if they are routinely taking these medications and wait to take their morning dose after the fasting blood draws
    - Efficacy, pharmacokinetic, and safety assessments will be performed at these visits
- Subjects randomized to receive single-blind Met IR will titrate to a dose of 1000 mg Met IR twice daily (BID) (2000 mg Met IR per day in equal divided doses) on Day 8 from a starting dose of 1000 mg Met IR qAM. On Day 8, study site personnel are to make a scheduled telephone call to the subject to remind the subject to increase the dose of treatment by adding the PM dose (Group G only). Treatment G will not be blinded from the study site personnel but the identity of the treatment will not be actively disclosed to subjects (single-blind).
- For Visit 4 (Week 2) and Visit 6 (Week 6):
    - Subjects are to administer their morning dose of study medication with food at least 4 hours before the blood collection at these visits and on the same day as these visits
    - Subjects who routinely take morning doses of approved antidiabetic medications are to administer these medications as typically scheduled before coming to the clinic
    - Subjects randomized to Group G are to wait to take the evening dose of study medication after the plasma metformin blood collection
    - Pharmacokinetic and safety assessments will be performed at these visits
- Subjects are to be instructed to monitor blood glucose as per the guidance of the investigator (or qualified designee) and as appropriate for their medical management, and to promptly report as directed any values associated with hypoglycemia or greater than the prespecified hyperglycemic thresholds defined below.

- Subjects should administer their doses of study medication at the beginning of a meal and at approximately the same time each day throughout the study unless instructed otherwise by the investigator. Subjects will be instructed to record the time and date of each dose of study medication and complete a dosing diary.
- A subject with a fasting plasma glucose or HbA1c value greater than a prespecified threshold as defined below is to have the test repeated within 3 to 7 days from the date the sample was collected. If the results from both tests are above the threshold, the subject is to be appropriately monitored and rescued with antidiabetic therapy as per the judgment of the investigator.
    - Fasting plasma glucose value greater than 270 mg/dL from Week 4 through Week 8
    - Fasting plasma glucose value greater than 240 mg/dL from >Week 8 through Week 16
    - HbA1c value increased by more than 1.5% relative to baseline from Week 4 through Week 16
    - HbA1c value greater than 11.0% from Week 4 through Week 16

EFFICACY ASSESSMENTS

- HbA1c values
- Fasting plasma glucose concentrations

PHARMACOKINETIC ASSESSMENTS

- Plasma metformin concentrations

SAFETY ASSESSMENTS

- Adverse events and concomitant medication reviews
- Physical examinations
- 12-lead electrocardiograms (ECGs)
- Vital signs (sitting systolic and diastolic blood pressure, heart rate, and body weight)
- Clinical laboratory measures (including anion gap, lactate, and vitamin B_{12} concentrations)

STATISTICAL CONSIDERATIONS

Analysis Populations

The following populations will be used for the summaries and analyses of the study data:

- **Enrolled:** The Enrolled Population will consist of subjects who take at least one dose of lead-in study medication during the lead-in period.
- **Randomized**: The Randomized Population will consist of subjects who are randomized on Day 1.
- **Intent-to-Treat (ITT)**: The ITT Population will consist of subjects who take at least one dose of randomized study medication. The ITT Population will be used for analyses of safety and for sensitivity analyses of primary, secondary, and additional endpoints.
- **Modified ITT (mITT)**: The mITT Population will consist of all ITT subjects who have at least one post-baseline value for HbA1c that is collected no more than 1 week after discontinuing study medication and prior to administration of any rescue medications for worsening hyperglycemia. The mITT Population will be the primary analysis population for the primary, secondary, and additional efficacy endpoints.
- **Evaluable**: The Evaluable Population will consist of mITT subjects who complete the study through Week 16 with no major protocol deviations and who never receive rescue medication for worsening hyperglycemia. The Evaluable Population will be used for sensitivity analyses of primary, secondary, and additional endpoints.

Study Endpoints

The primary endpoint is the change in HbA1c from baseline to Week 16.

The secondary endpoints are the:

- Change in fasting plasma glucose concentrations from baseline to Week 16
- Proportion of subjects achieving HbA1c ≤7% at Week 16

Additional endpoints are:

- Change in HbA1c from baseline to intermediate visits over the 16-week treatment period
- Proportion of subjects achieving HbA1c ≤6.5% at Week 16
- Change in fasting plasma glucose concentrations from baseline to intermediate visits over the 16-week treatment period

- Proportion of subjects requiring rescue medications for worsening hyperglycemia during the treatment period
- Plasma fasting metformin concentrations

The safety endpoints are:

- Adverse events, with a focus on treatment-emergent adverse events, defined as those occurring at or after the first administration of randomized study medication at Visit 3 through Study Termination, or existing prior to the time of and worsening after the time of the first administration of randomized study medication
- Changes from baseline in ECG results
- Changes from baseline in concomitant medications and physical examination findings
- Changes from baseline in vital signs and clinical laboratory measures (including plasma lactate, anion gap, and vitamin B_{12})

Hypothesis Testing for the Primary Endpoint

The primary hypotheses to be tested for the primary endpoint (change from baseline to Week 16 in HbA1c) are based on assessment of Met DR relative to placebo (P, treatments A and B pooled). Define μ_j, j = C, D, E, and F, and μ_p as the changes from baseline to Week 16 in HbA1c for treatments C, D, E, F, and placebo, respectively. The primary hypotheses to be tested for the primary endpoint are

$$H_{0i}: \mu_j = \mu_P$$

$$H_{ai}: \mu_j \neq \mu_P$$

where (i,j) = (1, C; 2, D; 3, E; 4, F).

The primary analysis population for analyses of the primary endpoint will be the mITT Population. Missing data will be treated as missing with no imputation and endpoint values based on measurements obtained after initiation of rescue medication for worsening hyperglycemia will be censored.

SAMPLE SIZE

The sample size for this study is based on analysis to be conducted using hypotheses H_{0i} and H_{ai} in the mITT Population. The power is calculated based on detecting a statistically significant difference for at least one Met DR treatment versus placebo. The assumptions for the calculation are shown below.

- Two-sided $\alpha = 0.05$
- Power = 90%
- Two sample t-test with a common SD = 1.195%
- True value of $\mu_i - \mu_P = -0.6\%$
- Balanced randomization
- Percentage of subjects with no data available = 5%

Using these assumptions, the number of subjects required is 85 in each treatment. After adjusting for the missing data rate, this results in approximately 92 randomized subjects in each treatment. Thus, the total sample size planned for the study is 552 randomized subjects.

SPONSOR: Elcelyx Therapeutics, Inc.

TABLE OF CONTENTS

1 INTRODUCTION 14
1.1 Description of Metformin DR 14
1.2 Clinical Experience with Metformin DR 14
1.3 Rationale for Conduct of the Study and Dosage Selection 15
1.3.1 Rationale for Conduct of the Study 15
1.3.2 Dosage Selection 15
2 STUDY OBJECTIVES 16
2.1 Primary Objective 16
2.2 Secondary Objectives 16
3 STUDY DESIGN 16
3.1 Design Description 16
3.2 Visit Structure 18
3.3 Study Duration 19
4 STUDY POPULATION 19
4.1 Population to Be Studied 19
4.2 Inclusion Criteria 19
4.3 Exclusion Criteria 20
4.4 Subject Restrictions 22
4.5 Prior and Concomitant Medications 23
4.5.1 Prior Medications 23
4.5.2 Concomitant Medications 23
5 STUDY MEDICATIONS 24
5.1 Formulation, Packaging, and Storage 24
5.2 Dispensing of Study Medication 24
5.3 Medication Administration Procedures and Route 24
5.4 Randomization Schedule and Blinding Procedures 25
5.5 Drug Accountability 25
5.6 Treatment Compliance 26
5.7 Study Medication Dosing Diaries 26
6 STUDY METHODS 26
6.1 Study Procedures 26
6.1.1 Visit WS: Washout Screening Procedures 27
6.1.2 Visit 1: Screening Procedures 28
6.1.3 Visit 2 (Week -2): Enrollment, Lead-in Period 29
6.1.4 Visit 3 (Day 1): Randomization 31
6.1.5 Visit 5 (Week 4), Visit 7 (Week 8), and Visit 8 (Week 12): Randomized Treatment Period 32
6.1.6 Visit 4 (Week 2) and Visit 6 (Week 6): Randomized Treatment Period 33
6.1.7 Visit 9 (Week 16): Randomized Treatment Period and Study Termination 33
6.1.8 Early Termination 34
7 ETHICAL SAFETY CONSIDERATIONS 35
7.1 Common Adverse Events 35
7.2 Standard of Care 35
7.3 Hypoglycemia 35
7.4 Hyperglycemia 36
7.5 Lactic Acidosis 37
8 EFFICACY ASSESSMENTS 37
9 PHARMACOKINETIC ASSESSMENTS 37
10 SAFETY ASSESSMENTS 37
10.1 Adverse Events and Subject Withdrawals 38
10.1.1 Adverse Events 38
10.1.1.1 Intensity 39

10.1.1.2 Causality 39
10.1.1.3 Pregnancy 39
10.1.2 Serious Adverse Events 39
10.2 Clinical Laboratory Evaluations 42
10.2.1 Chemistry 43
10.2.2 Hematology 43
10.2.3 Urinalysis 43
10.3 Other Safety Assessments 43
10.3.1 Vital Signs 43
10.3.2 Physical Examinations 44
10.3.3 Electrocardiogram 44
10.3.4 Pregnancy Testing 44
11 BLOOD VOLUME 44
12 DISCONTINUING SUBJECTS 44
12.1 Unblinding 45
13 DATA MANAGEMENT 45
13.1 General Guidelines 45
14 STATISTICAL CONSIDERATIONS 46
14.1 Analysis Populations 46
14.2 Study Endpoints 46
14.3 Summary of Study Subjects 47
14.3.1 Demographic and Baseline Characteristics 47
14.3.2 Subject Disposition, Medical History, Protocol Deviations, and Study Medication Compliance 47
14.3.3 Concomitant Medications 48
14.4 Methods for Handling Data Issues 48
14.4.1 Defining the Study Baseline 48
14.4.2 Handling of Multiple Observations 48
14.4.3 Handling of Missing Data 49
14.5 Efficacy Analyses 50
14.5.1 General Considerations 50
14.5.2 Hypotheses Testing for the Primary Efficacy Endpoint 50
14.5.3 Analyses of the Primary Efficacy Endpoint 50
14.5.4 Analyses of Secondary and Additional Endpoints 51
14.5.5 Dose Response Analyses 51
14.5.6 Multiplicity 51
14.5.7 Subgroup Analyses 52
14.6 Safety Analyses 52
14.6.1 Extent of Exposure 52
14.6.2 Adverse Events 52
14.6.3 Clinical Laboratory Evaluations 53
14.6.4 Physical Examinations, 12-Lead ECGs, and Vital Signs 53
14.7 Interim Analysis 53
14.8 Randomization and Stratification 53
14.9 Justification of Sample Size 53
15 INVESTIGATOR AND SPONSOR OBLIGATIONS 54
15.1 Medical Supervision 54
15.2 Study Initiation and Discontinuation 54
15.3 Laboratory Accreditation 55
15.4 Data Reporting 55
15.5 Study Monitoring 55
15.6 Record Retention 56
15.7 Deviation from the Protocol 56
15.8 Quality Assurance 57

16 DISCLOSURE OF DATA AND PUBLICATIONS 57
17 ETHICAL CONSIDERATIONS 58
17.1 Institutional Review Board 58
18 INFORMED CONSENT 58
19 FINAL REPORT 58
REFERENCE LIST 59
INVESTIGATOR'S ACCEPTANCE 60

LIST OF IN-TEXT TABLES

Table 1: Randomized Treatment Arms and Dose Regimens (Protocol LCRM112) 17

LIST OF IN-TEXT FIGURES

Figure 1: Study Design (Protocol LCRM112) 18

LIST OF APPENDICES

Appendix 1. Protocol Amendment 1: Summary of Changes 61
Appendix 2. Protocol Amendment 2: Summary of Changes 67
Appendix 3. Protocol Amendment 3: Summary of Changes 72
Appendix 4. Protocol Amendment 4: Summary of Changes 74
Appendix 5. Study Plan (Protocol LCRM112) 77

LIST OF ABBREVIATIONS

ALT	alanine aminotransferase
AST	aspartate aminotransferase
ATC	Anatomical Therapeutic Chemical
βhCG	human chorionic gonadotropin, beta subunit
BID	twice daily
BMI	body mass index
CFR	Code of Federal Regulations
CRO	Contract Research Organization
ECG	electrocardiogram
eCRF	electronic case report form
EDC	Electronic Data Capture
eGFR	estimated glomerular filtration rate
FDA	Food and Drug Administration
GCP	Good Clinical Practices
GLM	general linear model
HbA1c	hemoglobin-specific A1c fraction
HCl	hydrochloride
HIPAA	Health Insurance Portability and Accountability Act
ICF	Informed Consent Form
ICH	International Conference on Harmonisation
IRB	Institutional Review Board
ITT	Intent-to-Treat
IUD	intrauterine contraceptive device
IWRS	interactive web response system
LOCF	last observation carried forward
MALA	metformin-associated lactic acidosis
MDRD	Modification of Diet in Renal Disease
Met DR	delayed-release metformin
Met IR	immediate-release metformin
Met XR	extended-release metformin
mITT	Modified Intent-to-Treat
qAM	once daily in the morning
SAE	serious adverse event
SAP	Statistical Analysis Plan
SAS	Statistical Analysis System
T2DM	type 2 diabetes mellitus
TEAE	treatment-emergent adverse event
ULN	upper limit of normal
WS	washout screening

1 INTRODUCTION

1.1 Description of Metformin DR

Metformin is an oral drug that has been used for more than 50 years to treat patients with type 2 diabetes mellitus (T2DM). The most common side effects of metformin are gastrointestinal. Lactic acidosis is a very rare risk of chronic metformin use (0.03 cases/1000 patient-years) that is fatal in approximately 50% of cases and linked to metformin exposure.[1] Circulating metformin is renally cleared and the risk of metformin-associated lactic acidosis (MALA) increases at high systemic concentrations. Thus, metformin is currently contraindicated in T2DM patients with renal impairment.

Recent discoveries indicate that metformin exerts its glucose-lowering effects predominantly through presystemic effects in the lower bowel including direct or indirect interactions with enteroendocrine L-cells.[2, 3] Importantly, systemic exposure to metformin is not required for pharmacologic activity. Given this finding, the sponsor is developing a delayed-release formulation of metformin (Met DR) with the aim of reducing systemic bioavailability of metformin while targeting delivery of the drug to the L-cells in the distal small intestine. The L-cell is sparsely located in the duodenum but increases in density from the duodenum to the rectum. When commercially available forms of metformin (metformin immediate-release [Met IR] and metformin extended-release [Met XR]) are orally administered, approximately 40% of the metformin dose is absorbed in the duodenum and upper jejunum with approximately 10% absorption in the ileum and colon.[4] The remainder of the dose may accumulate in the mucosa of the bowel and is ultimately eliminated in the feces. In contrast, Met DR uses a pH 6.5 enteric-coated technology to bypass the region of the gut with the highest absorption (proximal small intestine) and targets delivery to the distal small intestine, a region with poor metformin absorption and higher L-cell density. Targeted delivery of metformin to the distal small intestine results in reduced bioavailability and allows for use of lower metformin doses. The resulting reduction in systemic exposure with Met DR may provide a better alternative for certain patients at risk of lactic acidosis due to metformin plasma accumulation (e.g., those with renal impairment or for populations at risk for declining renal function such as the elderly).

1.2 Clinical Experience with Metformin DR

Met DR has been evaluated in proof-of-concept studies in healthy volunteers (Study LCPOC6) and subjects with T2DM (Studies LCPOC10 and LCRM104) and in a single daily-dose crossover study that compared metformin pharmacokinetics of Met DR, Met IR, and Met XR formulations (Study LCRM103). The results of these short-term clinical studies indicate that Met DR administration results in substantially reduced systemic exposure to metformin relative to currently approved metformin-containing products, yet maintains comparable efficacy in lowering blood glucose and enhancing gut hormone release

in T2DM patients. Importantly, Study LCRM104 demonstrated that providing the total daily dose of Met DR once a day (with the morning or evening meal) in subjects with T2DM is similarly effective as twice a day (with the morning and evening meals) in lowering plasma glucose, providing further evidence that metformin's effects do not rely on sustained metformin plasma concentrations.

The results from Study LCRM105, a 12-week, Phase 2 study in subjects with T2DM, indicated that 600, 800, and 1000 mg/day doses of Met DR produced clinically relevant reductions in fasting plasma glucose and maintenance of glycemic control at both Week 4 and Week 12 relative to baseline. Mean hemoglobin-specific A1c fraction (HbA1c) differences from placebo at 12 weeks were -0.48%, -0.45% and -0.35% for 600 mg, 800 mg, and 1000 mg Met DR, respectively. The mean HbA1c difference from placebo for the 1000 mg Met XR reference arm was -0.45%.

As part of a clinical development program to determine the safety of Met DR in renally impaired patients, a single-dose study was conducted to assess metformin plasma exposure and lactate levels in T2DM subjects with normal renal function and mild, moderate, or severe renal impairment (Study LCRM101). Consistent with earlier studies with Met DR, a single dose of 1000 mg Met DR resulted in statistically significantly lower peak and overall systemic metformin exposures compared to 1000 mg Met XR.

1.3 Rationale for Conduct of the Study and Dosage Selection

1.3.1 Rationale for Conduct of the Study

The purpose of the present study is to compare the glycemic effects of Met DR to placebo in subjects with T2DM at 16 weeks. The study is designed to evaluate several doses of Met DR compared to placebo. A single-blind reference treatment of 2000 mg Met IR per day administered as equal divided doses (1000 mg Met IR twice daily [BID]) will also be included.

1.3.2 Dosage Selection

Based on Phase 2 studies, 1200 mg Met DR is expected to be well tolerated and provide efficacy results similar to 2000 mg Met IR with lower plasma exposure. 600 mg Met DR has previously been shown to have similar magnitude of effect as 1000 mg Met XR on fasting plasma glucose over 12 weeks. The glycemic effect of 1500 mg Met DR will be evaluated to explore the higher end of the dose range. 2000 mg Met IR will be included as a single-blind active reference treatment.

In the present study, subjects will receive total daily doses of 600, 900, 1200, or 1500 mg Met DR or 2000 mg Met IR. The single-blind Met IR group will titrate to 2000 mg from a starting dose of 1000 mg (see Section 5.3). The recommended Met IR starting dose for patients with T2DM is 500 mg BID or 850 mg once a day, given with meals, and the

maximum recommended dose is 2550 mg/day given in divided doses BID or three times a day with meals. Thus, the target dosages selected for this study are within the recommended daily dose for patients diagnosed with T2DM.

2 STUDY OBJECTIVES

2.1 Primary Objective

The primary objective of this study is to:

- Assess the effect of Met DR compared to placebo on the change in HbA1c at Week 16 of treatment

2.2 Secondary Objectives

The secondary objectives of this study are to:

- Assess the effect of Met DR compared to placebo at Week 16 of treatment on the following:
    - Change in fasting plasma glucose concentrations
    - Proportion of subjects achieving HbA1c ≤7%
- Compare the effects of Met DR and Met IR at Week 16 of treatment on the following:
    - Change in HbA1c
    - Change in fasting plasma glucose concentrations
    - Proportion of subjects achieving HbA1c ≤7%
- Assess the safety and tolerability of Met DR over the 16-week treatment period

3 STUDY DESIGN

3.1 Design Description

LCRM112 is a Phase 2, randomized, double-blind, parallel-group, multicenter, placebo-controlled, 7-arm, dose-ranging study that will be conducted in approximately 552 subjects with T2DM. Enrollment will target ≥30% of subjects with an HbA1c of ≥8.5%. The study will include a 2-week single-blind lead-in period and a 16-week double-blind treatment period. During the lead-in period, subjects will be enrolled and will receive placebo once daily in the morning (qAM) (1× 600 mg matched placebo tablet) at the beginning of the morning meal. During the treatment period, subjects will be randomly assigned to 1 of 7 treatment arms (Groups A, B, C, D, E, F, and G) in the ratio of 1:1:2:2:2:2:2, respectively as shown in Table 1. There will be 2 placebo arms (Groups A and B), 4 Met DR arms (Groups C, D, E, and F) and 1 Met IR arm (Group G). Groups A, B, C, D, E, and F will be

double-blinded. Group G will be single-blinded. Randomization will be stratified by screening HbA1c (<8.5% or ≥8.5%).

Table 1: Randomized Treatment Arms and Dose Regimens (Protocol LCRM112)

Treatment Arm	N	Morning Dose Regimen [1]	Evening Dose Regimen [1]
A (Placebo)	46	2× 600 mg matched placebo	NA
B (Placebo)	46	1× 600 mg matched placebo<br>1× 900 mg matched placebo	NA
C (600 mg Met DR qAM)	92	1× 600 mg Met DR<br>1× 600 mg matched placebo	NA
D (900 mg Met DR qAM)	92	1× 600 mg matched placebo<br>1× 900 mg Met DR	NA
E (1200 mg Met DR qAM)	92	2× 600 mg Met DR	NA
F (1500 mg Met DR qAM)	92	1× 600 mg Met DR<br>1× 900 mg Met DR	NA
G (2000 mg Met IR)	92	1× 1000 mg Met IR	1× 1000 mg Met IR

Abbreviations: BID=twice daily; qAM=once daily in the morning; NA=not applicable
[1] Number of tablets × tablet dose strength.

A detailed schedule of study procedures by visit is presented in Appendix 5.

The overall study design is shown in Figure 1.

Figure 1: Study Design (Protocol LCRM112)

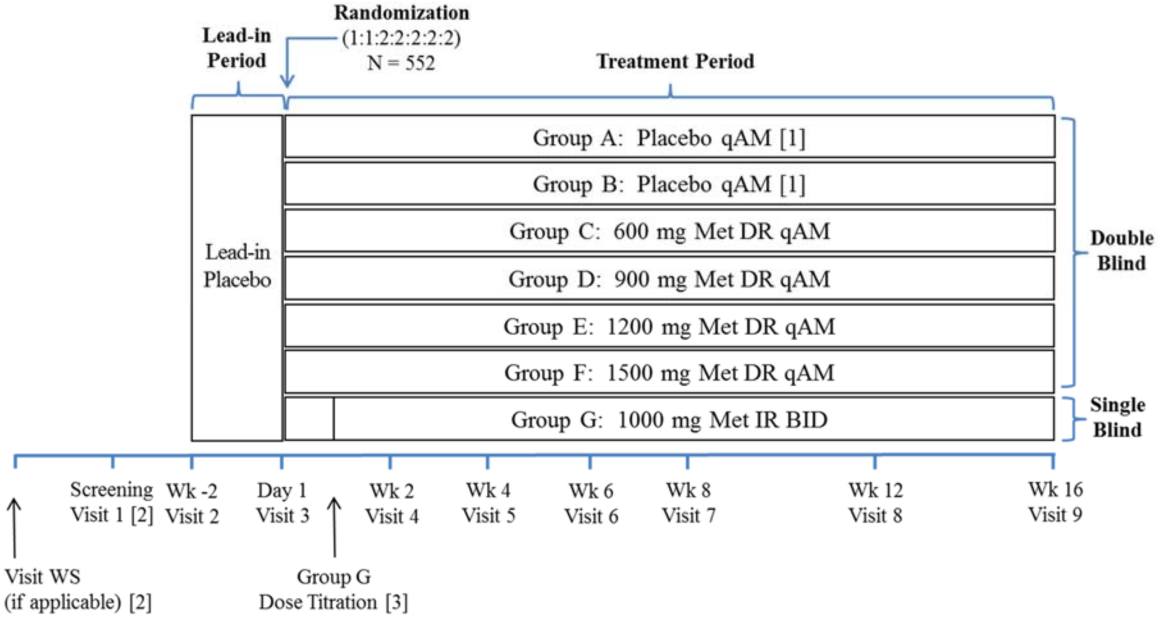

Abbreviations: BID=twice daily; qAM=once daily in the morning; WS=Washout Screening

[1] Placebo tablets will be identical in size and appearance to Met DR tablets to maintain the treatment blind. The 2-week lead-in period will use 600 mg matched placebo tablets (1 tablet qAM).

[2] Subjects may wash out of prior metformin therapy after initiation of screening procedures at Visit WS (Washout Screening) before Visit 1 (Screening) if appropriate based on the investigator's clinical judgment. Such subjects may qualify for study enrollment at Visit 1 (Screening) after a 60- to 75-day metformin wash out period. Visit 2 (Week -2) will occur within 2 weeks following Visit 1 (Screening).

[3] The Met IR group will titrate to a dose of 1000 mg Met IR BID (2000 mg Met IR per day in equal divided doses) on Day 8 from a starting dose of 1000 mg Met IR qAM.

3.2 Visit Structure

The study will consist of a screening visit (Visit 1), a lead-in visit (Visit 2), and 7 treatment visits (Visits 3 through 9). Subjects may wash out of prior metformin therapy after initiation of screening procedures at Visit WS (Washout Screening) before Visit 1 (Screening), if appropriate based on the investigator's clinical judgment. Such subjects may qualify for study enrollment at Visit 1 (Screening) after a 60- to 75-day metformin wash out period. Visit 1 procedures may be conducted over >1 day. Visit 2 (Week -2) will occur within 2 weeks following Visit 1 (Screening). Visit 3 (Day 1) will occur 2 weeks (±3 days) following Visit 2 (Week -2). All visits subsequent to Visit 3 will occur at 2- or 4-week intervals (±3 days relative to Visit 3 [Day 1]). Visits 2, 3, 5, 7, 8, and 9 (Week -2, Day 1, and Weeks 4, 8, 12, and 16) are to occur between 0600 and 1000 hours if possible. Visits 4 and 6 (Weeks 2 and 6) are to occur at least 4 hours after subjects administer the morning dose of study medication and before subjects who are randomized to single-blind Group G administer the evening dose of study medication.

3.3 Study Duration

The total study duration is approximately 18 to 32 weeks depending on the number of days between Visit WS (if applicable), Visit 1, and Visit 2. The study will include a screening period of up to 3 months, a 2-week lead-in period, and a 16-week treatment period.

4 STUDY POPULATION

4.1 Population to Be Studied

Approximately 552 males and non-pregnant females, at least 25 years of age, with T2DM (HbA1c 7.0% to 10.5%) and an estimated glomerular filtration rate (eGFR) of ≥60 mL/min/1.73 m^2 who are not taking metformin for at least 2 months prior to Screening (Visit 1) are to be randomized in this study. Specific study requirements are listed in Sections 4.2 and 4.3. Subjects must meet laboratory eligibility requirements both at Visit 1 and Visit 2; see Sections 6.1.2 and 6.1.3 for further guidance on retesting.

Subjects may wash out of prior metformin therapy after initiation of screening procedures at Visit WS (Washout Screening) if appropriate based on the investigator's clinical judgment. It is recommended (but not required) that subjects have HbA1c 7.0 to 9.5% (inclusive) prior to initiation of the wash out period. Such subjects may qualify for study enrollment at Visit 1 (Screening) after a 60- to 75-day metformin wash out period; for such subjects, eligibility for the study based on thresholds outlined in the inclusion and exclusion criteria (Sections 4.2 and 4.3) is to be determined based on assessments at the time of Visit 1 (Screening).

4.2 Inclusion Criteria

Each subject must meet the following criteria to be enrolled in this study.

1. Is at least 25 years old at Visit 1 (Screening).
2. Is male, or is female and meets all of the following criteria:
    a. Not breastfeeding
    b. Negative pregnancy test result (human chorionic gonadotropin, beta subunit [βhCG]) at Visit 1 (Screening) (not applicable to post-menopausal or surgically sterile females)
    c. Surgically sterile, postmenopausal, or if of childbearing potential, must practice and be willing to continue to practice appropriate birth control (defined as a method which results in a low failure rate of less than 1% per year when used consistently and correctly such as implants, injectables, oral contraceptives, some intrauterine contraceptive devices [IUDs], sexual abstinence, tubal ligation, condom with spermicide, or a vasectomized partner) during the entire duration of the study.

3. Body mass index (BMI) 20.0 to 45.0 kg/m^2 (inclusive) at Visit 1 (Screening).
4. Has a physical examination with no clinically significant abnormalities as judged by the investigator.
5. Has T2DM and an HbA1c of 7.0% to 10.5%, inclusive.
6. Has an eGFR value of ≥60 mL/min/1.73 m^2 based on the Modification of Diet in Renal Disease (MDRD) equation:
   eGFR (mL/min/1.73 m^2) = 175 x $(S_{cr,\,std})^{-1.154}$ x $(Age)^{-0.203}$ x (0.742 if female) x (1.212 if African American).
7. Either is not treated with or has been on a stable treatment regimen with any of the following medications for a minimum of 3 months prior to Visit 1 (Screening):
   a. Thiazolidinedione, sulfonylurea, dipeptidyl peptidase-4 inhibitors, and alpha-glucosidase inhibitors
   b. Hormone replacement therapy (female subjects) and testosterone (male subjects)
   c. Oral contraceptives (female subjects)
   d. Antihypertensive agents
   e. Lipid-lowering agents
   f. Thyroid replacement therapy
   g. Antidepressant agents.
8. Ability to understand and willingness to adhere to protocol requirements.

4.3 Exclusion Criteria

Any subject who meets any of the following criteria is to be excluded from the study.

1. Has a clinically significant medical condition as judged by the investigator that could potentially affect study participation and/or personal well-being, including but not limited to the following conditions:
   a. Hepatic disease
   b. Gastrointestinal disease, including but not limited to:
      i. History or presence of inflammatory bowel disease or other severe gastrointestinal disease, particularly those that may impact gastric emptying, such as gastroparesis and pyloric stenosis
      ii. Surgical gastrointestinal procedure that may impact the gut hormonal response to study medication such as gastric bypass surgery or gastric banding surgery
   c. Endocrine disorder (T2DM is allowed)

   d. Cardiovascular disease
   e. Central nervous system diseases
   f. Psychiatric or neurological disorders
   g. Organ transplantation
   h. Chronic or acute infection
   i. Orthostatic hypotension, fainting spells or blackouts
   j. Allergy or hypersensitivity
2. A history of diabetic ketoacidosis or hyperosmolar non-ketotic hyperglycemia within the past year.
3. Prior major surgery of any kind within 6 months of Visit 1 (Screening).
4. A history of >3% weight change within 3 months of Visit 1 (Screening).
5. A clinical laboratory test (clinical chemistry, hematology, or urinalysis) abnormality, other than that related to T2DM, judged by the investigator to be clinically significant.
6. An alanine aminotransferase (ALT) or aspartate aminotransferase (AST) result >2.5 × upper limit of normal (ULN) or a bilirubin result >1.5 × ULN.
7. A physical, psychological, or historical finding that, in the investigator's opinion, would make the subject unsuitable for the study.
8. Has been treated, is currently being treated, or is expected to require or undergo treatment with any of the following excluded medications:
   a. Metformin within 2 months of Visit 1 (Screening)
   b. Insulin within 2 weeks of Visit 1 (Screening) or for more than 1 week within 3 months of Visit 1 (Screening)
   c. Glucagon-like peptide-1 receptor agonists or sodium-glucose co-transporter 2 inhibitors within 3 months of Visit 1 (Screening)
   d. Drugs known to affect body weight, including prescription medications (e.g., phentermine/topiramate [QSYMIA®], orlistat [XENICAL® or ALLI®], lorcaserin [BELVIQ®], bupropion/naltrexone [CONTRAVE®]), and over-the-counter anti-obesity agents within 3 months of Visit 1 (Screening)
   e. Systemic corticosteroids by oral, intravenous, or intramuscular route; or potent, inhaled, or intrapulmonary steroids known to have a high rate of systemic absorption within 3 months of Visit 1 (Screening)
   f. Planned use of any drug treatment that affects gastric pH (prescription or over-the-counter), such as H2-receptor antagonists and proton pump inhibitors, after Visit 2

(Week -2), or planned chronic use (i.e., more than twice per week) of any antacids after Visit 2 (Week -2).

g. Cationic drugs that are eliminated by renal tubular secretion (e.g., amiloride, digoxin, morphine, procainamide, flecainide, quinidine, quinine, ranitidine, triamterene, trimethoprim, and vancomycin) within 1 week of Visit 1 (Screening)

h. Iodinated contrast dye within 1 week prior to Visit 1 (Screening)

i. Investigational drug within 2 months (or five half-lives of the investigational drug, whichever is greater) of the date of the first dose of randomized study medication

j. Met DR or double-blind matching placebo for Met DR at any time prior to Visit 1 (Screening)

9. Currently abuses drugs or alcohol or has a history of abuse that in the investigator's opinion would cause the individual to be noncompliant with study procedures.
10. Had a blood transfusion or experienced significant blood loss (i.e., >500 mL), including loss due to blood donation, within 2 months prior to Visit 1 (Screening), or is planning to donate blood during the study.
11. Has known immune system based allergies or hypersensitivity to any component of study treatment. A history of gastrointestinal intolerance to metformin is not exclusionary.
12. Is employed by Elcelyx Therapeutics, Inc. (that is an employee, contract worker, or designee of the company).
13. Has a fasting plasma glucose value >270 mg/dL at Visit 1 (Screening), Visit 2 (Week -2), and an unscheduled visit to be completed within 1 week following Visit 2. The unscheduled visit is to be completed only for subjects with a fasting plasma glucose value >270 mg/dL at Visit 1 and Visit 2.

4.4 Subject Restrictions

Once screened and qualified for entry, subjects are to be instructed as follows:

- Take no new prescription medications or over-the-counter preparations without prior approval of the investigator (who may contact the sponsor for consultation); for restrictions on concomitant medications, refer to Section 4.3
- Maintain stable doses of allowed concomitant medication(s) throughout study unless instructed otherwise by the investigator (who may contact the sponsor for consultation)
- Do not take any antacids (e.g., Rolaids® and TUMS®) within 2 hours of study medication administration
- Avoid strenuous exercise and alcohol 24 hours prior to each study visit
- For Visits 2, 3, 5, 7, 8, and 9 (Week -2, Day 1, and Weeks 4, 8, 12, and 16):

  - Arrive at the clinic after fasting (no food or drink except water) overnight for at least 10 hours
  - Bring study medication to the clinic (except for Visit 2), and wait to take the morning dose after the fasting blood draws (except for Visit 9; study medication will not be administered on the day of this visit)
  - Bring the morning dose of approved antidiabetic medications to the clinic if these medications are routinely taken and wait to take the morning dose after the fasting blood draws
- For Visit 4 (Week 2) and Visit 6 (Week 6):
  - Administer the morning dose of study medication with food at least 4 hours before the blood collection at these visits and on the same day as these visits
  - Administer the morning dose of approved antidiabetic medications as typically scheduled before coming to the clinic if these medications are routinely taken
  - Wait to take the evening dose of study medication after the plasma metformin blood collection (Group G only)

The subject may be rescheduled to return to the clinic within 72 hours for their visit if they had not followed the above restrictions. Every effort should be made to keep rescheduled visits within the window for the respective study visit.

The sponsor is to be contacted in a timely manner if study-site personnel are informed of any restriction violations. The sponsor is to decide whether a subject with restriction violations will be allowed to continue study participation.

4.5 Prior and Concomitant Medications

4.5.1 Prior Medications

Prior medications (prescription medications and over-the-counter preparations within the last 3 months) are to be recorded at Visit 1 (Screening).

4.5.2 Concomitant Medications

Subjects are to follow the medication restrictions outlined in the inclusion and exclusion criteria (Sections 4.2 and 4.3) and in the subject restrictions section (Section 4.4) during the study. Dosages for allowed concomitant medications should be maintained constant during the study, unless instructed otherwise by the investigator or a treating physician.

5 STUDY MEDICATIONS

5.1 Formulation, Packaging, and Storage

Study LCRM112 will evaluate the following study medications.

Study medication for the lead-in period:

- Placebo (EFP0084): placebo-matched tablets for EFB0082

Study medications for the treatment period:

- Placebo (EFP0084): placebo-matched tablets for EFB0082
- Placebo (EFP0085): placebo-matched tablets for EFB0083
- Met DR (EFB0082): 600 mg metformin hydrochloride (HCl) delayed-release tablets
- Met DR (EFB0083): 900 mg metformin HCl delayed-release tablets
- Met IR (Glucophage®): 1000 mg metformin HCl immediate-release tablets

Study medications will be supplied in blister packs, should be stored as indicated on the label, and should be used only as directed by study-site personnel.

5.2 Dispensing of Study Medication

Study materials will be provided to subjects by the investigator or medically qualified sub-investigator named on Form Food and Drug Administration (FDA) 1572, or other qualified study-site personnel at Visit 2 and according to the randomization scheme at Visits 3, 5, 7, and 8. Under no circumstances are the investigator or study-site personnel to allow study medication to be used other than as directed by the protocol or administered to persons other than subjects participating in the study.

If material is allocated to a subject incorrectly, the sponsor must be notified.

5.3 Medication Administration Procedures and Route

Subjects are to be instructed to administer study medication orally with water, as intact tablets (swallowed whole, do not cut, chew, or crush). Doses should be taken at approximately the same time each day unless instructed otherwise. Refer to Table 1 for the dose regimen for each treatment group.

For Visits 2, 3, 5, 7, 8, and 9 (Week -2, Day 1, and Weeks 4, 8, 12, and 16), subjects are to bring their study medication with them to the clinic (except for Visit 2), and wait to take their morning dose after the fasting blood draws (except for Visit 9; study medication will not be administered on the day of this visit) as described in Section 6 and Appendix 5.

Below are example instructions for self-administration of study medication:

- Groups A-F: Take one tablet, once daily, at the beginning of your morning meal, starting today until the day before your Visit 3 appointment. Starting after your Visit 3 appointment, take two tablets, once daily, at the beginning of your morning meal through the end of the study.
- Group G: Take one tablet, once daily, at the beginning of your morning meal, starting today until a week after your Visit 3 appointment. Starting a week after your Visit 3 appointment, take one tablet, once daily, at the beginning of your morning meal and one tablet, once daily, at the beginning of your evening meal through the end of the study.
- When you take your tablets, swallow them whole, with water, and do not cut, crush or chew your tablets

Subjects randomized to receive single-blind Met IR will titrate to a dose of 1000 mg Met IR BID (2000 mg Met IR per day in equal divided doses) on Day 8 from a starting dose of 1000 mg Met IR qAM. On Day 8, study site personnel are to make a scheduled telephone call to the subject to remind the subject to increase the dose of treatment by adding the PM dose (Group G only).

5.4 Randomization Schedule and Blinding Procedures

Sufficient study medication will be provided to the study site for enrollment of all subjects. Study medication kits will be labeled with unique package numbers. The study-site pharmacist or other medically qualified personnel must contact the interactive web response system (IWRS) to randomly assign subjects and for kit assignment. Randomization is to be stratified by Screening HbA1c (<8.5% or ≥8.5%).

Lead-in study medication will not be blinded from the investigators but the identity of the treatment will not be disclosed to subjects (single-blind). Groups A, B, C, D, E, and F will be blinded to subjects, investigators, and the sponsor (double-blind). As the tablets for Group G will be sourced from commercial supply, their appearance will be different than Met DR and placebo tablets. The dosing regimen (1 tablet BID at the target dose) will also be different for Group G than for the double-blind groups (2 tablets qAM). Thus, Treatment G will not be blinded from the investigator or site staff, but the identity of the treatment will not be actively disclosed to subjects (single-blind). Because all study medication will be packaged in blister packs and the identity of each treatment will not be actively disclosed to subjects, the difference in the appearance of the tablets and the dosing regimen for Treatment G is not expected to result in breaking of the blind at the subject level.

5.5 Drug Accountability

Study-site personnel will be responsible for study medication accountability. Upon receipt of study medications, study-site personnel are to open the shipment, and verify that the amount

and identity of the contents match that stated in the enclosed shipping form. The sponsor (or designee) is to be notified immediately about any irregularities, discrepancies, or damage.

All study medications administered to each subject are to be documented on a sponsor provided study medication accountability form or equivalent sponsor approved document. Upon completion of the study, unused remaining study medication is to be returned to the sponsor (or designee) or, if prior sponsor approval is obtained, disposed of in accordance with applicable site procedures. Study-site personnel must maintain documentation of any missing or unreturned study medication. The final disposition of all study medication received at the site is to be documented.

5.6 Treatment Compliance

Subjects who do not comply with dosing instructions by taking the morning dose of study medication prior to the fasting blood draws at Visits 3, 5, 7, 8, or 9 (Day 1 and Weeks 4, 8, 12, and 16) should have their visit rescheduled as instructed in Section 4.4 (Subject Restrictions). The time of the most recent dose of study medication prior to the plasma metformin blood draws at Visits 3 through 9 must be documented on the source documents and electronic case report form (eCRF).

Subject compliance with study medication dosing will be assessed at the site during each visit starting at Visit 3. Subjects who miss a dose should be instructed to take the dose only if less than half the time has passed until the next scheduled dose. If more than half the time has passed until the next scheduled dose, the subject should be instructed not to take the missed dose (i.e., do not double dose). Subjects should be reminded of the importance of dosing at approximately the same time each day as instructed. Returned study medication should be counted at each visit starting at Visit 3 and compliance with the scheduled dose should be assessed based on non-directed questioning and study medication accountability. If compliance is not between 85% and 115%, inclusive, the subject will be counseled by the investigator about the importance of compliance with the regimen (Visits 3 through 8). The sponsor retains the right to require the withdrawal of any subject who violates the protocol.

5.7 Study Medication Dosing Diaries

Subjects are to record the time and date of each dose of study medication and complete a dosing diary. At Visit 2, subjects are to be instructed on how to use the dosing diary.

6 STUDY METHODS

6.1 Study Procedures

The procedures to be performed during the study are listed by visit in Appendix 5.

Specific procedures to be performed during the study are described in detail by visit in this section.

6.1.1 Visit WS: Washout Screening Procedures

Subjects may wash out of prior metformin therapy after initiation of screening procedures at Visit WS (Washout Screening) if appropriate based on the investigator's clinical judgment. It is recommended (but not required) that subjects have HbA1c 7.0 to 9.5% (inclusive) prior to initiation of the wash out period. Such subjects may qualify for study enrollment at Visit 1 (Screening) after a 60- to 75-day metformin wash out period; for such subjects, eligibility for the study based on thresholds outlined in the inclusion and exclusion criteria (Sections 4.2 and 4.3) is to be determined based on assessments at the time of Visit 1 (Screening).

At Visit WS, subjects are to arrive at the clinic after fasting (no food or drink except water) overnight for at least 10 hours. The following procedures are to be performed:

- Obtain signatures for the Informed Consent Form (ICF) and Health Insurance Portability and Accountability Act (HIPAA) Authorization form prior to performing any protocol related procedures
- Record complete medical history, including menopausal status for females
- Review all prior medications (prescription medications and over-the-counter preparations within the last month), current medications, and adverse events
- Measure vital signs (sitting systolic and diastolic blood pressure, heart rate, and body weight)
- Perform an abbreviated physical examination and obtain a 12-lead electrocardiogram (ECG)
- Measure height and calculate BMI
- Assess subject eligibility for metformin wash out based on inclusion and exclusion criteria and the investigator's clinical judgment
- Collect blood samples for the following assessments:
    - HbA1c
    - Fasting plasma glucose and lactate
    - Clinical chemistry and hematology
- Collect urine for the following assessments:
    - Urinalysis
    - βhCG for female subjects of childbearing potential

- Review clinical status per the investigator's standard of care, including home blood glucose monitoring (refer to Section 7.2)

Individuals are to be disqualified if results of any clinical laboratory test or ECG are abnormal and clinically significant as judged by the investigator or medical monitor. Individuals may re-qualify within 5 days of Visit WS following an abnormal test result by having that test repeated once with acceptable results as judged by the investigator and medical monitor (or designees).

When Visit WS results are available, individuals will be notified by study-site personnel of their wash out eligibility status. Those who qualify will be instructed to discontinue prescribed metformin within 7 days of Visit WS and will be eligible to return to the study site to be screened at Visit 1 (Screening) after completion of the 60- to 75-day metformin wash out period.

6.1.2 Visit 1: Screening Procedures

At Visit 1, subjects are to arrive at the clinic after fasting (no food or drink except water) overnight for at least 10 hours. The following procedures are to be performed:

- Obtain signatures for the ICF and HIPAA Authorization forms prior to performing any protocol related procedures (if not obtained at Visit WS)
- Record complete medical history, including menopausal status for females (if not obtained at Visit WS)
- Review all prior medications (prescription medications and over-the-counter preparations within the last 3 months), current medications, and adverse events
- Measure vital signs (sitting systolic and diastolic blood pressure, heart rate, and body weight)
- Perform physical examination and obtain a 12-lead ECG
- Measure height (if not obtained at Visit WS) and calculate BMI
- Assess subject eligibility based on inclusion and exclusion criteria
- Collect blood samples for the following assessments:
    - HbA1c
    - Fasting plasma glucose and lactate
    - Clinical chemistry and hematology
- Collect urine for the following assessments:
    - Urinalysis

  - βhCG for female subjects of childbearing potential
- Review clinical status per the investigator's standard of care, including home blood glucose monitoring (refer to Section 7.2)

Individuals are to be disqualified if results of any clinical laboratory test or ECG are abnormal and clinically significant as judged by the investigator or medical monitor. Individuals may re-qualify for study enrollment within 2 weeks of Screening following an abnormal test result by having that test repeated once with acceptable results as judged by the investigator and medical monitor (or designees).

Individuals who do not meet study requirements outlined in the inclusion and exclusion criteria (Sections 4.2 and 4.3) may re-screen for the study with a new screening number after 2 weeks if appropriate based on the investigator's clinical judgment.

When all of the screening results are available, individuals will be notified by study-site personnel of their eligibility status. Those who qualify will be eligible to return to the study site to be enrolled at Visit 2.

6.1.3 Visit 2 (Week -2): Enrollment, Lead-in Period

Visit 2 (Week -2) will occur within 2 weeks following Visit 1 (Screening), between 0600 and 1000 hours if possible. Subjects are to arrive at the clinic after fasting (no food or drink except water) overnight for at least 10 hours. Subjects are to bring their morning dose of approved antidiabetic medications with them to the clinic if they are routinely taking these medications and wait to take their morning dose after the fasting blood draws. The following procedures are to be performed:

- Measure vital signs (sitting systolic and diastolic blood pressure, heart rate, and body weight)
- Collect blood samples for the following assessments:
  - HbA1c
  - Fasting plasma glucose and lactate
  - Clinical chemistry and hematology
- Collect urine for the following assessments:
  - Urinalysis
  - βhCG for female subjects of childbearing potential
- Review medications and adverse events
- Review clinical status per the investigator's standard of care, including home blood glucose monitoring. The investigator (or qualified designee) is to confirm based on home

glucose monitoring results whether the participant continues to be suitable for enrollment (refer to Section 7.2)

- Confirm study eligibility
- Enroll eligible subjects
- Dispense lead-in study medication and provide dosing instructions
- Administer lead-in study medication
- Provide dosing diary instruction and record the date and time of the first dose of lead-in study medication

Subjects may be discharged from the clinic after the visit's procedures are completed.

Individuals are to be withdrawn from lead-in if results of any clinical laboratory test fails eligibility thresholds outlined in the inclusion and exclusion criteria (Sections 4.2 and 4.3), unless relevant criteria defined below are met.

- Subjects who have a fasting plasma glucose value >270 mg/dL at Visit 1 (Screening) and Visit 2 (Week -2) may have the test repeated at an unscheduled visit within 1 week following Visit 2. The repeat fasting plasma glucose value must be ≤ 270 mg/dL for the subject to continue in the study.
- If the result of the HbA1c test at Visit 2 fails eligibility thresholds outlined in inclusion criterion 5 (HbA1c of 7.0% to 10.5% inclusive), individuals may re-qualify for randomization by having the test repeated once at an unscheduled visit within 7 days following Visit 2 if appropriate based on the investigator's clinical judgment in consultation with the medical monitor (or designees). The repeat HbA1c value must be 7.0% to 10.5% inclusive for the subject to continue in the study.
- If the result of the eGFR test at Visit 2 fails the eligibility threshold outlined in inclusion criterion 6 (eGFR of ≥60 mL/min/1.73 m^2), the subject is to be withdrawn unless the value is assessed as not a clinically significant change from the qualifying Visit 1 value by the investigator in consultation with the medical monitor (or designees).
- If the result of the liver function tests at Visit 2 fails eligibility thresholds outlined in exclusion criteria 6 (ALT >2.5 x ULN, AST >2.5 x ULN, and/or bilirubin results >1.5 x ULN), the subject is to be withdrawn unless the value is assessed as not a clinically significant change from the qualifying Visit 1 value by the investigator in consultation with the medical monitor (or designees).

For all clinically significant clinical laboratory test abnormalities, individuals may re-qualify for randomization by having the test repeated once at an unscheduled visit within 7 days following Visit 2 if appropriate based on the investigator's clinical judgment in consultation

with the medical monitor (or designees) with acceptable results as judged by the investigator and medical monitor (or designees).

Subjects who discontinue from the study during the lead-in period are not required to return to the clinic for an Early Termination visit.

6.1.4 Visit 3 (Day 1): Randomization

Visit 3 (Day 1) is to be scheduled at 2 weeks ±3 days relative to Visit 2 (Week -2) between 0600 and 1000 hours if possible. Subjects are to arrive at the clinic after fasting (no food or drink except water) overnight for at least 10 hours. Subjects are to bring their study medication and morning dose of any approved antidiabetic medications with them to the clinic and wait to take their morning doses after the fasting blood draws. The following procedures are to be performed:

- Measure vital signs (sitting systolic and diastolic blood pressure, heart rate, and body weight)
- Collect blood samples for the following assessments:
    - HbA1c
    - Fasting plasma glucose and lactate
    - Plasma metformin
    - Clinical chemistry and hematology
    - Vitamin B_{12}
- Collect urine for the following assessments:
    - Urinalysis
    - βhCG for female subjects of childbearing potential
- Review concomitant medications and adverse events
- Review clinical status per the investigator's standard of care, including home blood glucose monitoring. The investigator (or qualified designee) is to confirm based on home glucose monitoring results whether the participant continues to be suitable for randomization (refer to Section 7.2)
- Confirm study eligibility
- Randomize eligible subjects
- Dispense randomized study medication and provide dosing instructions
- Administer randomized study medication
- Collect unused study medication and previously dispensed study medication containers

- Assess study medication compliance and review dosing diary

The investigator (or qualified designee) is to confirm based on adequate compliance with study procedures whether the participant continues to be suitable for randomization.

Subjects may be discharged from the clinic after the visit's procedures are completed.

Subjects randomized to receive single-blind Met IR will titrate to a dose of 1000 mg Met IR BID (2000 mg Met IR per day in equal divided doses) on Day 8 from a starting dose of 1000 mg Met IR qAM. On Day 8, study site personnel are to make a scheduled telephone call to the subject to remind the subject to increase the dose of treatment by adding the PM dose (Group G only). Treatment G will not be blinded from the study site personnel but the identity of the treatment will not be actively disclosed to subjects (single-blind).

6.1.5 Visit 5 (Week 4), Visit 7 (Week 8), and Visit 8 (Week 12): Randomized Treatment Period

Visits 5, 7, and 8 are to be scheduled at 4, 8, and 12 weeks ± 3 days relative to Visit 3 (Day 1) between 0600 and 1000 hours if possible. Subjects are to arrive at the clinic after fasting (no food or drink except water) overnight for at least 10 hours. Subjects are to bring their study medication and morning dose of any approved antidiabetic medications with them to the clinic, and wait to take their morning doses of study and antidiabetic medications after the fasting blood draws. The following procedures are to be performed:

- Measure vital signs (sitting systolic and diastolic blood pressure, heart rate, and body weight)
- Collect blood samples for the following assessments:
  - HbA1c
  - Fasting plasma glucose and lactate
  - Plasma metformin
  - Clinical chemistry
- Review concomitant medications and adverse events
- Review clinical status per the investigator's standard of care, including home blood glucose monitoring (refer to Section 7.2)
- Dispense randomized study medication and review dosing instructions
- Administer randomized study medication
- Collect unused study medication and previously dispensed study medication containers
- Assess study medication compliance and review dosing diary

Subjects may be discharged from the clinic after each visit's procedures are completed.

6.1.6 Visit 4 (Week 2) and Visit 6 (Week 6): Randomized Treatment Period

Visit 4 and Visit 6 are to be scheduled at 2 weeks and 6 weeks ± 3 days relative to Visit 3 (Day 1). Subjects are to administer their morning dose of study medication with food at least 4 hours before the blood collection at these visits and on the same day as these visits. Subjects who routinely take morning doses of approved antidiabetic medications are also to administer these medications as typically scheduled before coming to the clinic. Subjects randomized to Group G are to wait to take the evening dose of study medication after the plasma metformin blood collection. The following procedures are to be performed:

- Measure vital signs (sitting systolic and diastolic blood pressure, heart rate, and body weight)
- Collect blood samples for the following assessment:
    - Plasma metformin
- Review concomitant medications and adverse events
- Review clinical status per the investigator's standard of care, including home blood glucose monitoring (refer to Section 7.2)
- Assess study medication compliance and review dosing diary

Subjects may be discharged from the clinic after each visit's procedures are completed.

6.1.7 Visit 9 (Week 16): Randomized Treatment Period and Study Termination

Visit 9 is to be scheduled at 16 weeks ±3 days relative to Visit 3 (Day 1) between 0600 and 1000 hours if possible. Subjects are to arrive at the clinic after fasting (no food or drink except water) overnight for at least 10 hours. Subjects are to bring their study medication and morning dose of any approved antidiabetic medications with them to the clinic, and wait to take their morning doses of antidiabetic medications after the fasting blood draws. Study medication will not be administered on the day of this visit. The following procedures are to be performed:

- Measure vital signs (sitting systolic and diastolic blood pressure, heart rate, and body weight)
- Perform physical examination and obtain a 12-lead ECG
- Collect blood samples for the following assessments:
    - HbA1c
    - Fasting plasma glucose and lactate
    - Plasma metformin
    - Clinical chemistry and hematology

  - Vitamin B_{12}
- Collect urine for the following assessments:
  - Urinalysis
  - βhCG for female subjects of childbearing potential
- Review concomitant medications and adverse events
- Review clinical status per the investigator's standard of care, including home blood glucose monitoring (refer to Section 7.2)
- Collect unused study medication and previously dispensed study medication containers
- Assess study medication compliance and review dosing diary

Subjects who complete all study procedures at Visit 9 are study completers and may be discharged from the clinic.

6.1.8 Early Termination

Subjects who are randomized at Visit 3 (Day 1) and who withdraw from the study prior to completion of Visit 9 are to complete early termination procedures in a timely manner. Subjects are to arrive at the clinic after fasting (no food or drink except water) overnight for at least 10 hours. Subjects are to bring their study medication and morning dose of any approved antidiabetic medications with them to the clinic, and wait to take their morning doses of antidiabetic medications after the fasting blood draws. Study medication will not be administered on the day of this visit. The following procedures are to be performed:

- Measure vital signs (sitting systolic and diastolic blood pressure, heart rate, and body weight)
- Perform physical examination and obtain a 12-lead ECG
- Collect blood samples for the following assessments:
  - HbA1c
  - Fasting plasma glucose and lactate
  - Plasma metformin
  - Clinical chemistry and hematology
  - Vitamin B_{12}
- Collect urine for the following assessments:
  - Urinalysis
  - βhCG for female subjects of childbearing potential

- Review concomitant medications and adverse events
- Review clinical status per the investigator's standard of care, including home blood glucose monitoring (refer to Section 7.2)
- Collect unused study medication and previously dispensed study medication containers
- Assess study medication compliance and review dosing diary

7 ETHICAL SAFETY CONSIDERATIONS

7.1 Common Adverse Events

Based on safety data from previous clinical studies, diarrhea, nausea, and vomiting are the most likely side effects (or risks) of metformin treatment. If episodes are severe, the investigator should contact the sponsor to discuss appropriate clinical management as well as the continued participation of subjects experiencing these events. Investigators should make frequent safety and tolerability assessments throughout study conduct.

7.2 Standard of Care

Subjects are to be instructed per the investigator site's standard of care at each visit on diet, exercise, and home blood glucose monitoring as well as on the signs and symptoms of hypoglycemia and on supplemental oral glucose treatment, if needed. Subjects will also be instructed to promptly report as directed any values associated with hypoglycemia as described in Section 7.3 or greater than the prespecified hyperglycemic thresholds defined in Section 7.4. Assessment and action should then occur as deemed appropriate by the investigator. The investigator (or designee) will review the glucose meter readings in accordance with the study site's standard of care at every visit.

7.3 Hypoglycemia

Hypoglycemia, a significant problem with other modalities of antidiabetic treatment, is rare when metformin is used as monotherapy, as metformin does not stimulate insulin secretion.

Specific criteria for monitoring hypoglycemia events have been designed to ensure subject safety and to closely monitor hypoglycemia. All subjects will be asked to record a blood glucose measurement if they experience symptoms of hypoglycemia (e.g., impaired autonomic responses such as tremulousness, sweating, palpitations, and hunger). Following appropriate treatment of the event, subjects will report the occurrence of a hypoglycemia event and the associated blood glucose value to study-site personnel. Study-site personnel will gather additional specific information about the reported event and then provide that information to the sponsor and investigator. If the investigator determines that a subject experienced symptoms consistent with hypoglycemia, the event should be documented in the subject's source documentation and the hypoglycemia adverse event eCRF page must be

completed. If a blood glucose value of <45 mg/dL is noted within the data (i.e., home blood glucose monitoring or fasting plasma glucose measures) that is asymptomatic and not previously recorded as an adverse event of hypoglycemia, the investigator should assess whether the circumstances around the value are consistent with hypoglycemia. If the assessment is that the value is consistent with hypoglycemia, the hypoglycemia adverse event eCRF page should be completed. If the hypoglycemic episode intensity is classified as severe or requires adjustments to other antidiabetic medications, the investigator is required to contact the sponsor to discuss appropriate clinical management as well as the continued participation of the subject in the study.

The criteria for evaluating the intensity of a hypoglycemic episode are the following:

MILD: Usually transient, requires no special treatment, and does not interfere with the subject's daily activities

MODERATE: Usually causes a low level of inconvenience or concern to the subject and may interfere with daily activities, but is usually ameliorated by simple therapeutic measures

SEVERE: Requires the assistance of another person to obtain treatment (may include intravenous glucose, intramuscular glucagon, or oral carbohydrate) for the event

Whenever the investigator is confident in making a unifying diagnosis, all related signs, symptoms, and abnormal test results associated with an episode of low plasma glucose concentration should be grouped together as a single adverse event in the source document and eCRF.

7.4 Hyperglycemia

A subject with a fasting plasma glucose or HbA1c value greater than a prespecified threshold as defined below is to have the test repeated within 3 to 7 days from the date the sample was collected. If the results from both tests are above the threshold, the subject is to be appropriately monitored and rescued with antidiabetic therapy as per the judgment of the investigator.

- Fasting plasma glucose value greater than 270 mg/dL from Week 4 through Week 8
- Fasting plasma glucose value greater than 240 mg/dL from >Week 8 through Week 16
- HbA1c value increased by more than 1.5% relative to baseline from Week 4 through Week 16
- HbA1c value greater than 11.0% from Week 4 through Week 16

The investigator may contact the medical monitor to discuss appropriate clinical management. If it is determined that the subject should be discontinued from the study due to loss of glucose control despite initiating rescue medication, the subject disposition eCRF page should report the following as the primary reason for Study Termination:

- Discontinuation due to loss of glucose control as defined in the protocol

The associated event will also be recorded as an adverse event on the adverse events eCRF page with action taken of study drug discontinued. Discontinuation due to loss of glucose control as defined in the protocol will be considered a withdrawal due to adverse event. Rescue medications may include dose adjustments to current antidiabetic medications and/or addition of thiazolidinediones, sulfonylureas, dipeptidyl peptidase-4 inhibitors, alpha-glucosidase inhibitors, insulin, and/or glucagon-like peptide-1 receptor agonists. Prescribed metformin and sodium-glucose co-transporter 2 inhibitors are not permitted as rescue medications.

7.5 Lactic Acidosis

A rare, but serious metabolic complication of lactic acidosis can occur due to metformin accumulation. When this condition occurs, it is fatal in approximately 50% of cases. The reported incidence of lactic acidosis in patients receiving metformin is very low (approximately 0.03 cases/1000 patient-years). Metformin is known to be substantially excreted by the kidney, and the risk of metformin accumulation and attendant lactic acidosis increases with the degree of impairment of renal function. Because this study excludes subjects with renal disease and moderate or severe dysfunction, metformin accumulation is unlikely and the risk of lactic acidosis is low.

8 EFFICACY ASSESSMENTS

Samples for the measurement of HbA1c and fasting plasma glucose concentrations will be collected according to the schedules presented in Appendix 5 and in Section 6. The centralized laboratory or sponsor will provide specific instructions for collection, processing, packaging, and shipping of all samples.

9 PHARMACOKINETIC ASSESSMENTS

Samples for the measurement of plasma metformin concentrations will be collected according to the schedules presented in Appendix 5 and in Section 6. The laboratory will provide specific instructions for collection, processing, packaging, and shipping of all samples.

10 SAFETY ASSESSMENTS

Safety will be assessed throughout the study by examination of adverse events and concomitant medication reviews, physical examinations, 12-lead ECGs, vital signs (sitting systolic and diastolic blood pressure, heart rate, and body weight), and clinical laboratory measures (including anion gap, lactate, and vitamin B_{12} concentrations).

10.1 Adverse Events and Subject Withdrawals

10.1.1 Adverse Events

An adverse event is any untoward medical occurrence in a clinical investigation after the subject has signed an ICF. An adverse event can therefore be any unfavorable and unintended sign (including an abnormal laboratory finding, for example), symptom, or disease temporally associated with the use of the study medication, whether or not considered related to the study medication.

ALL ADVERSE EVENTS THAT OCCUR AFTER THE SUBJECT HAS SIGNED THE ICF WILL BE RECORDED ON SOURCE DOCUMENTS. ADVERSE EVENTS FOR SUBJECTS WHO ARE ENROLLED IN THE STUDY WILL BE ENTERED ON SOURCE DOCUMENTS AND/OR eCRFs. Adverse events include those reported spontaneously by the subject and those noted incidentally or as the result of non-directed questioning by the investigator or study-site personnel. To avoid vague, ambiguous, or colloquial expressions, the adverse event should be recorded on the source documents and/or eCRFs using standard medical terminology that is as specific as possible, rather than the subject's own words. Whenever the investigator is confident in making a unifying diagnosis, all related signs, symptoms, and abnormal test results should be grouped together as a single adverse event in the source document and/or eCRF (e.g., cough and rhinitis should be reported as an "upper respiratory tract infection").

All clinically significant abnormalities noted upon physical examination, and clinical laboratory, ECG, and vital sign assessments that occur during the study and were not present prior to the signing of the ICF, should be reported as an adverse event, except for abnormalities present at Screening that may be considered part of the medical history. In addition, all clinically significant adverse events that continue at Study Termination should be followed up by the investigator and evaluated with additional tests if necessary, until the underlying cause is diagnosed or resolution occurs. Follow-up information should be recorded on the source documents and reported to the sponsor. Adverse events will be evaluated for intensity and causal relationship with the use of the study medication by the investigator.

Adverse events that occur following completion of study/early termination procedures should be recorded on the adverse event page of the source document and/or eCRF only if the investigator considers the event as clinically significant and as related to study medication or study procedures. All serious adverse events (SAEs) that occur within 30 days of administration of the last dose of study medication (regardless of causality) must be reported immediately. Concomitant medications used following study termination procedures should be recorded only if relevant to treatment of subjects for events described above.

10.1.1.1 Intensity

The intensity of each adverse event will be characterized as MILD, MODERATE, or SEVERE, as follows:

MILD: Usually transient, requires no special treatment, and does not interfere with the subject’s daily activities.

MODERATE: Usually causes a low level of inconvenience or concern to the subject and may interfere with daily activities, but is usually ameliorated by simple therapeutic measures.

SEVERE: Interrupts a subject’s usual daily activities and generally requires systemic drug therapy or other treatment.

10.1.1.2 Causality

The investigator will grade the association of the adverse event as UNRELATED or RELATED to study medication. The following criteria should be considered for determining relatedness:

UNRELATED: The adverse event is judged to be produced by the subject's clinical state or by other therapies administered to the subject.

RELATED: The adverse event is judged to be related to the administration of study medication.

10.1.1.3 Pregnancy

For female subjects, a pregnancy test will be performed according to the schedules presented in Appendix 5 and Section 6 unless the subject is post-menopausal or has had a hysterectomy. Subjects who become pregnant during the study will be withdrawn from the study immediately upon confirmation of pregnancy. The reason for withdrawal will be documented on the source document and/or eCRF page as a “protocol violation”. Pregnancy information will be collected on the appropriate forms as supplied by the sponsor. These subjects will be required to return to the study site for early termination procedures. In addition, the sponsor will monitor all documented pregnancies through outcome when possible. While pregnancy itself is not considered an adverse event or SAE, any pregnancy complications will be recorded as an adverse event or SAE.

10.1.2 Serious Adverse Events

ANY SAE THAT OCCURS AFTER THE SIGNING OF THE ICF THROUGH 30 DAYS AFTER ADMINISTRATION OF THE LAST DOSE OF STUDY MEDICATION MUST BE REPORTED IMMEDIATELY (WITHIN 24 HOURS OF KNOWLEDGE).

Any adverse event that results in any of the following outcomes will be considered an SAE. The following outcomes are defined according to Code of Federal Regulations (CFR) Title 21 Part 312.32.

- Death
- Life threatening situation (subject was at risk of death at the time of the event. This does not refer to an event that might have caused death if it was of greater intensity.)
- Inpatient hospitalization or prolongation of inpatient hospitalization
- Persistent or significant incapacity or substantial disruption of the ability to conduct normal life functions
- Congenital anomaly or birth defect
- Important medical events that may not result in death, be life threatening, or require hospitalization but may jeopardize the subject and may require medical or surgical intervention to prevent one of the above outcomes (based upon appropriate medical judgment), e.g., allergic bronchospasm requiring intensive treatment in an emergency room or at home, blood dyscrasias or convulsions that do not result in inpatient hospitalization, or the development of drug dependency or drug abuse

If an SAE occurs, the investigator should first initiate appropriate procedures to treat the subject. Study-site personnel should make every effort (within 24 hours) to obtain the investigator's clinical opinion about the information available for the event. However, if the information cannot be obtained, the sponsor should still be contacted with all available information. Specifically, the investigator must assess the following:

- That the term chosen to describe the event is as specific and accurate as possible and represents the investigator's unifying diagnosis (when applicable) for the event
- The relationship of the event to study medication (related or unrelated)
- The relationship of the event to study conduct (related or unrelated). Examples of SAEs related to study conduct may include an event caused by a study procedure (e.g., blood draw, imaging) or an event that occurred as a result of discontinuing medications in a washout period
- The treatment phase during which the event occurred (e.g., randomized treatment period, washout)
- Whether the event is life threatening or is persistently or significantly disabling
- Whether hospitalization or prolongation of hospitalization was required due to the event
- Description of the event
- Intensity of the event

- That the onset date and time of the first symptoms and/or signs, as well as the investigations performed, are consistent with and reflect the clinical start of the event and the unifying diagnosis for the event
- Treatment received
- The end date (if applicable) of the resolution of all symptoms, signs, abnormal test results; determination of a final diagnosis; or the date of return to a new stable baseline
- Outcome and planned follow-up
- Subject's status in clinical study participation (e.g., continuing study, early terminator)

The following information is also required:

- Investigator's name and study-site number
- Protocol number and title
- Subject's initials and subject identification number
- Subject's date of birth, sex, and race
- Dates of administration of study medication(s) and dosage (if applicable)
- Subject's medical history relevant to the SAE
- Concomitant medications, including dosage, route of administration, duration of therapy, and indication

In cases where the investigator learns of the event after its occurrence and resolution, the time and circumstances of the event should be recorded. The reporting requirements must still be followed.

All SAEs occurring from the time of informed consent through 30 days after administration of the last dose of study medication must be reported to Medpace **within 24 hours** of the knowledge of the occurrence (this refers to any adverse event that meets any of the aforementioned serious criteria).

To report the SAE, the SAE form should be completed electronically in the Electronic Data Capture (EDC) system for the trial. When the form is completed, Safety personnel will be notified electronically and will retrieve the form. If EDC is unavailable, the completed paper back-up SAE report form should be faxed or emailed to Medpace (fax number/email address listed below) within 24 hours of awareness. When the EDC system becomes available, the SAE information must be entered within 24 hours of the system becoming available. If the event meets serious criteria and it is not possible to access the form, an email should be sent to Medpace Safety at medpace-safetynotification@medpace.com or call the Medpace SAE

hotline (phone number listed below). Safety personnel are available for SAE reporting on a 24-hour basis. Reports are reviewed during normal business hours.

Safety Contact Information:

Medpace Clinical Safety
Medpace, Inc.
5375 Medpace Way
Cincinnati, OH 45227

Medpace SAE hotline:
Telephone: +1-800-730-5779, ext. 2999 **or** +1-513-579-9911, ext. 2999
Facsimile: +1-866-336-5320 **or** +1-513-579-0444
e-mail: medpace-safetynotification@medpace.com

The investigator is required to submit SAE reports to the Institutional Review Board (IRB) in accordance with local requirements. All investigators involved in trials using the same investigational product will receive any safety alert notifications for onward submission to their local IRB as required. All reports sent to investigators will be blinded.

The investigator must continue to follow the subject until the SAE has subsided, or until the condition becomes chronic in nature, stabilizes (in the case of persistent impairment), or the subject dies. Within 24 hours of receipt of follow-up information, the investigator must update the SAE form electronically in the EDC system for the trial and submit any supporting documentation (e.g., laboratory test reports, subject discharge summary, or autopsy reports) to Medpace Safety personnel via fax or email. When the SAE form is updated, Safety personnel will be notified electronically and will retrieve the form. If it is not possible to access the form, refer to procedures outlined above for initial reporting of SAEs.

10.2 Clinical Laboratory Evaluations

The investigator will evaluate all screening and safety laboratory reports, and will sign and date the review. Any out of range laboratory results should be assessed for clinical significance. The investigator should follow all clinically significant laboratory abnormalities occurring during the study that were not present at baseline. These abnormalities should be evaluated with additional tests, if necessary, until the underlying cause is diagnosed or resolution occurs. The diagnosis and resolution date must be reported to the sponsor.

Samples for clinical chemistry, hematology, urinalysis, lactate, and vitamin B_{12} will be collected according to the schedules presented in Appendix 5 and in Section 6. The laboratory will provide specific instructions for collection, processing, packaging, and shipping of all samples.

10.2.1 Chemistry

Chemistry assessments will include the following for the chemistry panel: urea nitrogen, creatinine, total protein, albumin, uric acid, total bilirubin, alkaline phosphatase, ALT, AST, gamma glutamyltranspeptidase, creatine phosphokinase, sodium, potassium, chloride, bicarbonate, phosphorus, and calcium (or other routine chemistry panels as approved by the sponsor). Additional chemistry assessments include lactate and vitamin B_{12}.

Anion gap will be calculated as: [Sodium] – ([Chloride] + [Bicarbonate])

10.2.2 Hematology

Hematology assessments will include the following: red cell count, hemoglobin, hematocrit, white cell count, platelets, differential count, mean cell volume, mean corpuscular hemoglobin, and mean corpuscular hemoglobin concentration (or other routine hematology assessments as approved by the sponsor).

10.2.3 Urinalysis

Urinalysis assessments will include the following: pH, specific gravity, glucose, blood, ketones, and protein (or other routine urinalysis as approved by the sponsor).

10.3 Other Safety Assessments

Clinically significant abnormalities in other observations related to safety must be followed up by the investigator and evaluated with additional tests if necessary, until the underlying cause is diagnosed or resolution occurs. The diagnosis and resolution date must be reported to the sponsor.

Measurements will be performed as discussed in this section and according to the schedules presented in Appendix 5 and Section 6.

10.3.1 Vital Signs

Vital sign measurements in this study will include sitting systolic and diastolic blood pressure, heart rate, and body weight. Blood pressure and heart rate should be measured after the subject rests for approximately 5 minutes and with the subject in a sitting position. The blood pressure measurement should be repeated after at least 30 seconds and the average of the two readings recorded.

Subjects should be lightly clothed (no shoes) when measuring body weight and the same scale should be used throughout the study. Subjects should void before body weight is measured.

10.3.2 Physical Examinations

All body systems listed in the source document will be evaluated.

10.3.3 Electrocardiogram

Parameters will include ventricular rate, PR interval, QRS duration, QT interval, and corrected QT interval using Bazett's and Fridericia's correction methods.

10.3.4 Pregnancy Testing

Female subjects of childbearing potential (not post-menopausal or surgically sterile) will provide urine for pregnancy tests. Study medication will not be administered until a negative result is obtained at Visit 2 (Week -2).

11 BLOOD VOLUME

During this study, blood will be drawn for various analytes and panels, including chemistry, hematology, and other safety assessments, and efficacy and pharmacokinetic assessments. The maximum amount of blood to be drawn during the entire study is expected to be approximately 170 mL for each subject and the maximum to be drawn at a single visit will be approximately 25 mL.

12 DISCONTINUING SUBJECTS

Every effort should be made to conduct all protocol-required procedures to complete the study. Subjects may be removed from the study for the following reasons:

- **Withdrawal of Consent:** Subject wishes to exercise the right to withdraw from the study as stated in the ICF (all subjects reserve the right to withdraw from the study without prejudice)
- **Adverse Event:** Subject experiences an adverse event that, in the investigator's opinion, necessitates withdrawal from the study
- **Loss of Glucose Control as defined in the protocol** (Section 7.4)
- **Investigator Decision:** Investigator feels it is in the subject's best interest to terminate participation for reasons other than an adverse event
- **Protocol Violation:** Subject is noncompliant with protocol procedures, becomes pregnant, violates study entry criteria, or starts an exclusionary concomitant medication
- **Lost to Follow-Up:** Subject fails to return for study visits and cannot be reached with reasonable, repeated attempts
- **Administrative Reasons:** Elcelyx Therapeutics, Inc., the FDA, or other regulatory authority discontinues the study protocol or the study site discontinues participation

Any withdrawal must be fully documented in the subject's source document and eCRF. The documentation must include the reason for the withdrawal and details of any sequelae (followed until symptoms resolve or improve, as appropriate). Withdrawals due to an adverse event must be documented on both the disposition page and adverse event page of the source document and eCRF.

When a subject is lost to follow-up (i.e., fails to return for study visits), a reasonable effort (e.g., documented by receipts for certified mailings) will be made to contact the subject to determine why the subject failed to return and to attempt to schedule the Early Termination visit.

12.1 Unblinding

For Met DR treatments and placebo, the sponsor, subject, and investigator (and all study-site personnel) are to be blinded to treatment allocation during the conduct of the study. As described in Section 5.4, Treatment G will not be blinded from the investigator or site personnel but the identity of the treatment will not be actively disclosed to subjects (single-blind). Every effort should be made to ensure that subjects remain blinded to their treatment. The investigator is to notify the sponsor if an event occurs that requires a subject's treatment assignment to be unblinded. The subject's treatment may be unblinded only after an irrevocable decision has been made to withdraw a subject from the study and if immediate knowledge of the study medication received is needed to optimize the clinical management of the subject.

13 DATA MANAGEMENT

13.1 General Guidelines

The sponsor may designate a Contract Research Organization (CRO) or specialty provider to perform data management.

Clinical study data will be reported (captured) by study-site personnel on eCRFs. The eCRF data will be entered by study-site personnel and then reviewed and electronically signed by an investigator listed on Form FDA 1572. All study-site personnel must use an electronic signature access method to enter, review, or correct study data. Electronic signature procedures shall comply with the CFR Title 21 Part 11and the International Conference on Harmonisation (ICH) Guidelines for Good Clinical Practices (GCP) (Topic E6, April 1996). Passwords and electronic signatures will be strictly confidential.

All eCRF data will be downloaded from the EDC system and reformatted into Statistical Analysis System (SAS) data sets. The sponsor's data management department will receive electronic transfers of laboratory data from a central laboratory as well as other data from third-party vendors as appropriate. The electronic data format of all transfers will be agreed upon with the sponsor.

The clinical monitoring personnel will verify data recorded in the EDC system with source documents at the study sites according to the data management plan and clinical monitoring plan. The data will be subjected to consistency and validation checks within the EDC system with supplemental validation following download to a SAS data set.

Adverse events and concomitant medications will be coded. The sponsor or designee will perform a medical safety review of the coding.

Completed eCRF images with a date- and time-stamped electronic audit trail indicating the user, the data entered, and any reason for change (if applicable) will be archived at the study site and archived with backup at the sponsor's site.

14 STATISTICAL CONSIDERATIONS

14.1 Analysis Populations

The following populations will be used for the summaries and analyses of the study data:

- **Enrolled**: The Enrolled Population will consist of subjects who take at least one dose of lead-in study medication during the lead-in period.
- **Randomized**: The Randomized Population will consist of subjects who are randomized on Day 1.
- **Intent-to-Treat (ITT)**: The ITT Population will consist of randomized subjects who take at least one dose of randomized study medication. The ITT Population will be used for analyses of safety and for sensitivity analyses of primary, secondary, and additional endpoints.
- **Modified ITT (mITT)**: The mITT Population will consist of all ITT subjects who have at least one post-baseline value for HbA1c that is collected no more than 1 week after discontinuing study medication and prior to administration of any rescue medications for worsening hyperglycemia. The mITT Population will be the primary analysis population for the primary, secondary, and additional efficacy endpoints.
- **Evaluable**: The Evaluable Population will consist of mITT subjects who complete the study through Week 16 with no major protocol deviations and who never receive rescue medication for worsening hyperglycemia. The Evaluable Population will be used for sensitivity analyses of primary, secondary, and additional endpoints.

14.2 Study Endpoints

The primary endpoint is the change in HbA1c from baseline to Week 16.

The secondary endpoints are the:

- Change in fasting plasma glucose concentrations from baseline to Week 16

- Proportion of subjects achieving HbA1c ≤7% at Week 16

Additional endpoints are:

- Change in HbA1c from baseline to intermediate visits over the 16-week treatment period
- Proportion of subjects achieving HbA1c ≤6.5% at Week 16
- Change in fasting plasma glucose concentrations from baseline to intermediate visits over the 16-week treatment period
- Proportion of subjects requiring rescue medications for worsening hyperglycemia during the treatment period
- Plasma fasting metformin concentrations

The safety endpoints are:

- Adverse events, with a focus on treatment-emergent adverse events, defined as those occurring at or after the first administration of randomized study medication at Visit 3 through Study Termination, or existing prior to the time of and worsening after the time of the first administration of randomized study medication
- Changes from baseline in ECG results
- Changes from baseline in concomitant medications and physical examination findings
- Changes from baseline in vital signs and clinical laboratory measures (including plasma lactate, anion gap, and vitamin B_{12})

14.3 Summary of Study Subjects

14.3.1 Demographic and Baseline Characteristics

Demographic and baseline characteristics will be summarized descriptively for each treatment using the Randomized Population. The demographic and baseline characteristics include, but are not limited to: sex, age, age group (<65 years or ≥65 years), race, ethnicity, body weight, height, BMI, BMI stratum (<30 kg/m^2 or ≥30 kg/m^2), HbA1c, HbA1c stratum (<8.5% or ≥8.5%), and fasting plasma glucose concentration.

14.3.2 Subject Disposition, Medical History, Protocol Deviations, and Study Medication Compliance

The numbers and percentages of subjects will be provided for each analysis population by treatment using the Randomized Population. For subjects who withdraw from the study early, the primary reason for withdrawal will be summarized by treatment. Study medication compliance will be summarized by treatment using the ITT Population. The numbers and percentages of subjects with protocol deviations will be summarized by deviation category

using the Randomized Population. All the following deviations will be summarized using the ITT population:

- Inclusion or exclusion criteria not satisfied
- Significant deviations related to study medication administration
- Non-permitted concomitant medications

14.3.3 Concomitant Medications

All medications will be coded using the World Health Organization Drug Dictionary, and Anatomical Therapeutic Chemical (ATC) classification system.

Medications will be classified according to the following definitions:

- Pre-treatment medication: medications stopped prior to the first dose of study medication at Visit 2 (Week -2).
- Lead-in medication: medications started on or after the date of the first single-blind dose of study medication at Visit 2 (Week -2) and prior to the date of the first dose of randomized study medication at Visit 3 (Day 1).
- Concomitant medication: medications taken during conduct of the treatment period, including:
    - Prior concomitant medication: medications started prior to the first dose of randomized study medication and continuing past Visit 3 (Day 1).
    - New concomitant medication: medications started on or after the date of the first dose of randomized study medication.

The number and percentage of ITT subjects taking concomitant medications during the 16-week treatment period will be summarized by treatment (and overall) and ATC classification (ATC level 2 and level 4).

14.4 Methods for Handling Data Issues

14.4.1 Defining the Study Baseline

In general, the baseline value for each variable is defined as the last measurement collected prior to the first dose of randomized study medication at Visit 3 (Day 1).

14.4.2 Handling of Multiple Observations

A subject may have multiple scheduled or unscheduled lab values for the same analyte associated with the same scheduled visit. To define the baseline, all scheduled or unscheduled lab values prior to the first dose of randomized study medication will be sorted

by collection time and the last sample collected in chronological order will be used as the baseline value.

For multiple post-baseline values associated with the same scheduled visit, only values from scheduled visits will be considered and the last sample collected in chronological order will be used in the summary or analysis of efficacy endpoints.

All results, scheduled or unscheduled, will be listed.

14.4.3 Handling of Missing Data

Missing safety data will be treated as missing with no imputation. For the primary analyses of the primary, secondary, and additional endpoints, missing data will be treated as missing with no imputation and analyses will be conducted in the mITT Population. In addition, endpoint values based on measurements obtained after initiation of rescue medication for worsening hyperglycemia will be censored for the primary analyses.

Supportive analyses of the primary, secondary, and additional endpoints will be conducted in the mITT population and missing data will be imputed using the last observation carried forward (LOCF) approach as follows:

1. For subjects who have missing data prior to completing all study procedures through Week 16, but have data collected for at least one post-baseline visit prior to Week 16, missing values at any visit up to Week 16 will be imputed using the values from their previous visit (scheduled or unscheduled including Early Termination) in accordance with the LOCF approach. In the LOCF approach, values collected no more than 1 week after discontinuation of randomized study medication and prior to administration of any rescue medication for worsening hyperglycemia are eligible to be carried forward. Values at Visit 3 (Day 1) will not be carried forward.
2. Endpoint values based on measurements obtained after initiation of rescue medication for worsening hyperglycemia will be censored. Thus, in addition to imputation of missing data, the LOCF method will also be applied to the censored values.

In addition, an analysis that uses the LOCF method in the ITT Population will also be conducted. In this analysis, endpoint values based on measurements obtained after rescue medication will not be censored. For subjects who have missing data prior to completing all study procedures through Week 16, but have data collected for at least one post-baseline visit prior to Week 16, missing values at any visit up to Week 16 will be imputed using the values from their previous visit (scheduled or unscheduled including Early Termination) in accordance with the LOCF approach regardless of whether the subject had already discontinued study medication more than one week prior to the value to be carried forward. Values at Day 1 will not be carried forward.

14.5 Efficacy Analyses

14.5.1 General Considerations

The mITT Population will be used for the analyses of all the endpoints (primary, secondary, additional). Efficacy analyses will also be conducted using the Evaluable and ITT Populations for selected endpoints, which will be defined in the Statistical Analysis Plan (SAP).

All summary tables for quantitative parameters will display n, mean, standard deviation, standard error, median, range (minimum and maximum), as well as number of missing data (if relevant). All summary tables for qualitative parameters will display counts, percentages and number of missing data (if relevant).

14.5.2 Hypotheses Testing for the Primary Efficacy Endpoint

The primary hypotheses to be tested for the primary endpoint (change from baseline to Week 16 in HbA1c) are based on assessment of Met DR relative to placebo (P, treatments A and B pooled). Define μ_j, j = C, D, E, and F, and μ_P as the changes from baseline to Week 16 in HbA1c for treatments C, D, E, F, and placebo, respectively. The primary hypotheses to be tested for the primary endpoint are

H_{0i}: $\mu_j = \mu_P$

H_{ai}: $\mu_j \neq \mu_P$

where (i,j) = (1, C; 2, D; 3, E; 4, F)

The primary analysis population for analyses of the primary endpoint will be the mITT Population. Missing data will be treated as missing with no imputation as described in Section 14.4.3 and endpoint values based on measurements obtained after initiation of rescue medication for worsening hyperglycemia will be censored.

14.5.3 Analyses of the Primary Efficacy Endpoint

The primary efficacy endpoint will be summarized descriptively for each treatment in the mITT Population using observed data with censoring of values after initiation of rescue medication for worsening hyperglycemia and also with missing and censored data imputation using LOCF. The primary analysis model will employ a repeated measures linear mixed-effects model. This model will be implemented for the mITT Population with observed data and censoring of values after initiation of rescue medication for worsening hyperglycemia. The model will have random subject effects, fixed class effects for treatment, time (i.e., week), treatment-by-time interaction, and HbA1c value at Screening as a covariate. The covariance structure to model the within-patient errors will be unstructured. If this analysis fails to converge, additional structures will be tested and the covariance structure with the

best fit as determined by Akaike's information criterion will be used in the primary analysis. The least squares (LS) means and 95% two-sided confidence intervals for LS means and LS mean differences will be obtained within the framework of this model. Supportive analyses will be conducted in the mITT and ITT Populations with the LOCF approach used to impute missing and censored data as described in Section 14.4.3 and also conducted in the Evaluable Population using observed data. The model employed for the supportive analyses will be a general linear model (GLM) with treatment as a factor and HbA1c value at Screening as a covariate. For all analyses, model validity will be assessed. If model assumptions are not adequately met, alternative analysis methods will be implemented including but not limited to nonparametric methods.

14.5.4 Analyses of Secondary and Additional Endpoints

The secondary and additional endpoints will be summarized descriptively for each treatment using the mITT Population in the same manner as described for the primary efficacy endpoint. The analysis models for the non-dichotomous endpoints will employ models, data imputation methods, and analysis populations similar to those described for the primary and supportive analyses of the primary efficacy endpoint, but will also include the baseline value corresponding to the dependent variable as a covariate. The LS means and 95% two-sided confidence intervals for LS means and LS mean differences will be obtained within the framework of each model. Analyses of dichotomous endpoints (the proportion of subjects achieving HbA1c targets of ≤7% and ≤6.5% at Week 16 and the proportion of subjects requiring rescue medication for worsening hyperglycemia during the treatment period) will employ a logistic regression model with the same model framework as described for the primary efficacy endpoint. Model validity will be assessed and alternative analysis methods implemented as appropriate.

14.5.5 Dose Response Analyses

The dose response for the primary and secondary endpoints will be depicted using graphical methods in the mITT and Evaluable Populations. Inferential testing for dose response will be conducted in these populations using both parametric and nonparametric methods.

14.5.6 Multiplicity

No adjustments for multiplicity will be made in testing the primary hypotheses or in testing secondary and additional endpoints. No adjustments for multiplicity will be applied in subgroup analyses.

14.5.7 Subgroup Analyses

Subgroup analyses of key efficacy endpoints will be conducted without adjustment for multiplicity. These analyses will be conducted using the framework of the previously described analysis models with additional inclusion of a factor for the subgroup and the treatment-by-subgroup factor interaction. For each subgroup factor, a test for the treatment-by-subgroup factor interaction will be performed using a likelihood ratio test comparing the models with and without the interaction term at the 0.1 significance level. Estimates arising from these models and corresponding 95% confidence intervals will be reported for each subgroup. Potential subgroups of interest will be defined based on demographic factors and baseline characteristics of interest in the study population such as but not limited to:

- Baseline HbA1c
- Demographic characteristics (e.g., age, sex, race, BMI)
- Prior use of metformin (yes/no)
- Prior total daily dose of metformin

14.6 Safety Analyses

The analysis of safety data will be performed using the ITT Population. Safety will be assessed by examination of adverse events, concomitant medications, physical examinations, 12-lead ECGs, vital signs, and clinical laboratory measures (including anion gap, lactate, and vitamin B_{12}).

14.6.1 Extent of Exposure

The duration of time on study and time on study medication for the ITT population will be summarized for each treatment using descriptive statistics. The total subject-years on study medication and total subject-years on study will also be included in this summary. The number of subjects on treatment for certain time intervals will also be summarized.

14.6.2 Adverse Events

All adverse events will be coded using the Medical Dictionary for Regulatory Activities.

Treatment-emergent adverse events (TEAEs) will be defined as those occurring at or after the first administration of randomized study medication at Visit 3 (Day 1) through Study Termination, or existing prior to the time of and worsening after the time of the first administration of randomized study medication. Adverse events prior to the first administration of study medication will be classified as pretreatment (non-treatment-emergent). TEAEs, TEAEs leading to withdrawals, and serious TEAEs will be summarized by treatment.

14.6.3 Clinical Laboratory Evaluations

All clinical laboratory measures obtained from hematology, clinical chemistry, urinalysis, anion gap, lactate, and vitamin B_{12} with changes from Screening will be listed and summarized by treatment, subject, and visit. Laboratory assessments that are outside of normal ranges will be flagged.

14.6.4 Physical Examinations, 12-Lead ECGs, and Vital Signs

All results will be listed by treatment, subject, and visit. Changes from Screening in ECG parameters will be summarized descriptively by treatment. Changes from baseline to subsequent study visits in vital signs will be summarized descriptively by treatment.

14.7 Interim Analysis

An interim analysis may be conducted once all subjects have either completed 4 weeks of treatment or discontinued the study prior to completing 4 weeks of treatment. The analysis, if conducted, will include the changes in fasting plasma glucose and HbA1c from baseline to Week 4. Results from the analysis will be used for planning purposes only. Results from the analysis will remain confidential from personnel actively involved in study conduct so that the blind can be maintained until the end of the treatment period. Procedures for controlling the Type I error rate for the interim and final analyses will be specified in the SAP.

14.8 Randomization and Stratification

Randomization will be conducted centrally using screening HbA1c as a stratification factor (<8.5%, ≥8.5%).

14.9 Justification of Sample Size

The sample size for this study is based on analysis to be conducted using hypotheses H_{0i} and H_{ai} in the mITT Population. The power is calculated based on detecting a statistically significant difference for at least one Met DR treatment versus placebo. The assumptions for the calculation are shown below.

- Two-sided $\alpha = 0.05$
- Power = 90%
- Two sample t-test with a common SD = 1.195%
- True value of $\mu_i - \mu_P = -0.6\%$
- Balanced randomization
- Percentage of subjects with no data available = 5%

Using these assumptions, the number of subjects required is 85 in each treatment. After adjusting for the missing data rate, this results in approximately 92 randomized subjects in each treatment. Thus, the total sample size planned for the study is 552 randomized subjects.

15 INVESTIGATOR AND SPONSOR OBLIGATIONS

15.1 Medical Supervision

Medical supervision is the responsibility of the investigator named on Form FDA 1572. The investigator may delegate day to day activities to a sub-investigator listed on Form FDA 1572, but retains overall responsibility for ensuring that the study is conducted properly and in accordance with the study protocol. A list will be maintained that includes all qualified persons to whom the investigator may delegate significant study related duties. The investigator is responsible for ensuring that drugs and devices are available for treating possible medical emergencies, or that emergency medical facilities are available and accessible. The investigator is responsible for ensuring that the study is conducted according to GCP, other applicable regulatory guidelines, and sound medical practices.

15.2 Study Initiation and Discontinuation

Prior to initiation of the study, the investigator must provide the sponsor with the following documents (copies of which must be retained by the investigator):

- Signed original copy of the protocol acceptance statement that commits the investigator to follow the protocol exactly and to conduct the study according to GCP
- Completed and signed original Form FDA 1572
- Current curriculum vitae, as indicated by version date in the footer or the investigator's signature and date. Also required is a current state license for the investigator and for other medically qualified sub-investigators
- Signed financial disclosure forms for the investigator and all sub-investigators listed on Form FDA 1572
- Signed copy of the IRB approval letter that lists the approved items
- List of the IRB members who voted on the approval, including their specialty and affiliation, or the IRB assurance numbers if the roster cannot be obtained
- Copy of the IRB-approved ICF
- Copy of the local laboratory license, certification, and reference range values for any determinations required by the protocol, if applicable

Upon receipt of all necessary paperwork, the sponsor will arrange for all study materials to be delivered to the study site. The sponsor or designee will train all personnel expected to be

involved in the study. This training may include a review of the study protocol and a review of overall responsibilities, including study medication accountability and study file maintenance.

The sponsor has the right to terminate the study at any time for any of the following reasons:

- Non-adherence of study-site personnel to the protocol or GCP
- Unavailability of the investigator or study-site personnel to the sponsor's monitoring personnel or designee(s)
- Other administrative reasons

Additionally, individual subjects may be excluded from the study if a medical records review indicates protocol violations or if other factors appear to jeopardize the validity of the study. Throughout the course of the study, the investigator is to make a reasonable effort to maintain the enrollment rate that was agreed upon with the sponsor. The investigator will also make a reasonable effort to enroll appropriate subjects. The sponsor may elect to terminate the study at a given site if the enrollment rate lags or if significant numbers of non-evaluable subjects are enrolled.

15.3 Laboratory Accreditation

The laboratory facility used for analysis of safety clinical laboratory samples must provide evidence of adequate licensure or accreditation. Copies of laboratory certification, licensure, and reference ranges (as appropriate) will be supplied to the sponsor prior to study initiation. The sponsor or designee should be notified of any changes in reference range values or certification/license renewal during the course of the study. If a central laboratory is used, the central laboratory will provide a copy of accreditation and related reference range values.

15.4 Data Reporting

Data for each subject will be recorded on the eCRF. If the source document is to be used as the eCRF for any data parameters, this is to be documented prior to study conduct. An eCRF must be completed for every subject randomized in the study. When data are complete, the investigator or medically qualified sub-investigator listed on Form FDA 1572 will apply his/her signature on the eCRF indicating he/she has reviewed and approves of the data collected on the eCRF.

15.5 Study Monitoring

The investigator will allow qualified sponsor representatives or designee(s) to conduct periodic audits of study documents and corresponding portions of office, clinical, and laboratory records for each subject at each study site. Reviews of study data may be

performed during routine monitoring visits or via electronic methods, both during the study and following study completion. These visits are to provide the sponsor with the opportunity to evaluate study progress; verify the accuracy and completeness of data; and ensure that all protocol requirements and investigator obligations are being fulfilled. Finally, any inconsistencies in the study records should be resolved during these visits or at the direction of the sponsor.

The sponsor may terminate study conduct if study-site personnel do not follow the protocol or GCP. Additionally, individual subjects may be excluded if medical records review indicates significant protocol deviations or if other factors appear to jeopardize the validity of the study.

15.6 Record Retention

The investigator is to maintain adequate records for the study, including subjects' source documents, eCRFs, medical records, laboratory reports, signed ICFs, study medication accountability records, safety reports, information regarding subjects who discontinued the study, and any other pertinent data. All records and reports will be retained by the investigator for at least 2 years after the last approval of a marketing application in an ICH region. If the marketing application is rejected or if the marketing application is not filed, the records must be retained for 2 years following notification by the sponsor that investigations have been discontinued and that the FDA and other government regulatory agencies have been notified. Records should be available for copying and inspection if requested by a properly authorized employee of the FDA or other government regulatory agency, in accordance with federal or other applicable regulations. The investigator must notify the sponsor immediately in the event of accidental loss or destruction of any study records.

Sponsor specific essential documents will be retained at least 2 years after the last approval of a marketing application in an ICH region or at least 2 years following the formal discontinuation of clinical development of the study medication. These documents should be retained for a longer period, however, if required by applicable country regulatory requirements. For example, subject identification codes must be retained for at least 15 years following the completion or discontinuation of the study, according to European Union Directive 91/507/EEC.

15.7 Deviation from the Protocol

The investigator is not to deviate from the protocol. In medical emergencies, the investigator will use medical judgment and will remove the subject from immediate hazard. The investigator will immediately notify the sponsor and IRB regarding the nature of the emergency and the course of action taken as appropriate based on the IRB's reporting criteria. The investigator is to notify the sponsor of any inadvertent protocol deviations upon

discovery and is to document the deviations appropriately. The sponsor assumes no liability for any unapproved deviations.

Major changes in the protocol initiated by the sponsor will be provided as an amendment and will be approved by the IRB prior to implementation.

15.8 Quality Assurance

The sponsor may conduct a discretionary quality assurance audit of this study. If such an audit occurs, the investigator agrees to allow the auditor direct access to all relevant documents and to allocate his or her time and that of study-site personnel to the auditor to discuss findings and any relevant issues. In addition, regulatory agencies may conduct a regulatory inspection of this study. If such an inspection occurs, the investigator agrees to inform the sponsor upon notification by the regulatory agency. The investigator agrees to allow the inspector direct access to all relevant documents and to allocate his or her time and that of study-site personnel to the inspector to discuss findings and any relevant issues. The investigator will allow sponsor personnel to be present to observe the inspection, if requested.

16 DISCLOSURE OF DATA AND PUBLICATIONS

Subjects' medical information obtained as a result of this study is considered confidential, and disclosure to third parties other than those noted below is prohibited. Subject to any applicable authorization(s), all reports and communications relating to subjects in this study will identify subjects only by initials and number. Medical information resulting from a subject's participation in this study may be given to the subject's personal physician, other authorized parties, or to the appropriate medical personnel responsible for the subject's welfare. Data generated in this study will be available for inspection on request by government regulatory agency auditors, the sponsor, the sponsor's medical monitor (or designee) and its corporate partners for the study medication, if any, and their designated representatives, the IRB, and other authorized parties.

If requested, the investigator agrees to furnish the sponsor or appropriate designee with complete subject identification in a confidential disclosure for long term follow-up if needed. This disclosure will be treated in accordance with applicable law, with strict adherence to professional standards of confidentiality and will be filed by the sponsor with adequate security and restricted accessibility.

All information concerning the study medication and the sponsor's operations (such as patent applications, formulas, manufacturing processes, basic scientific data, or other information supplied by the sponsor and not previously published) are considered confidential and shall remain the sole property of the sponsor. The investigator agrees to use this information only in conducting this study and to not use it for other purposes without the sponsor's prior written consent.

The information developed in this clinical study may be disclosed by the sponsor as required, to authorized parties, other clinical investigators, private companies, and government agencies.

Any information, inventions, discoveries (whether patentable or not), innovations, suggestions, ideas, and reports made or developed by the investigator(s) as a result of conducting this study shall be promptly disclosed to the sponsor and shall be the sole property of the sponsor. The investigator agrees, upon the sponsor's request and at the sponsor's expense, to execute such documents and to take such other actions as the sponsor deems necessary or appropriate to obtain patents in the sponsor's name covering any of the foregoing.

The results of this study may be published under the direction of the sponsor. Results will not be published without the sponsor's prior review and approval.

17 ETHICAL CONSIDERATIONS

17.1 Institutional Review Board

The study will be conducted in accordance with the Declaration of Helsinki (1964) and all its amendments.

The protocol and ICF will be reviewed and approved by a duly constituted IRB before individuals are screened for study entry. The investigator will ensure that all aspects of the IRB review are conducted in accordance with current institutional, local, and national regulations. A letter documenting the IRB approval will be provided to the sponsor prior to initiation of the study. Amendments to the protocol will be subject to the same requirements as the original protocol. The investigator will submit all periodic reports and updates that the IRB may require, including any final closeout reports. The investigator will inform the IRB of any reportable adverse events.

18 INFORMED CONSENT

Each individual will be provided with oral and written information describing the nature, purpose and duration of the study, participation/termination conditions, and risks and benefits. Prior to initiation of any study-related procedures, subjects will sign and date the ICF to participate in the study. The signed original ICF will be retained with the study site's records and each subject will receive a copy of each form they have signed.

19 FINAL REPORT

All data, including subject characteristics, methodology, and clinical findings, will be presented in a final clinical study report to be prepared by the sponsor or designee.

REFERENCE LIST

1. Bristol-Myers Squibb Company. Glucophage®, Glucophage® XR [package insert], Princeton, NJ 08543, USA, Rev January 2009.
2. Graham GG, Punt J, Arora M, Day RO, Doogue MP, Duong JK, Furlong TJ, Greenfield JR, Greenup LC, Kirkpatrick CM, Ray JE, Timmins P, Williams KM. Clinical pharmacokinetics of metformin. Clin Pharmacokinet. 2011 Feb;50(2):81-98.
3. Stepensky D, Friedman M, Raz I, Hoffman A. Pharmacokinetic-pharmacodynamic analysis of the glucose-lowering effect of metformin in diabetic rats reveals first-pass pharmacodynamic effect. Drug Metab Dispos. 2002 Aug;30(8):861-8.
4. Vidon N, Chaussade S, Noel M, Franchisseur C, Huchet B, Bernier JJ. Metformin in the digestive tract. Diabetes Res Clin Pract. 1988 Feb 19;4(3):223-9.

INVESTIGATOR'S ACCEPTANCE

**Study Title:**	**A RANDOMIZED, DOUBLE-BLIND, PARALLEL-GROUP, MULTICENTER, PLACEBO-CONTROLLED, DOSE-RANGING STUDY TO EVALUATE THE GLYCEMIC EFFECTS, SAFETY, AND TOLERABILITY OF METFORMIN DELAYED-RELEASE IN SUBJECTS WITH TYPE 2 DIABETES MELLITUS**
**Final Date:**	18 May 2015
**Amendment 1:**	24 July 2015
**Amendment 2:**	08 October 2015
**Amendment 3:**	30 November 2015
**Amendment 4:**	18 January 2016
**Amendment 5:**	30 March 2016

I have read the protocol described above. I agree to comply with all applicable regulations and to conduct the study as described in the protocol. I agree to its terms and will conduct the study according to Good Clinical Practices.

____
Investigator Name (Printed)

____________________________________ ____________________________
Investigator Signature Date

Appendix 1. Protocol Amendment 1: Summary of Changes

Date: 24 July 2015

Amendment Purpose: The purpose of this amendment is to remove the metformin delayed-release (Met DR) twice daily (BID) treatment arm, add a Met DR 1500 mg once daily in the morning (qAM) treatment arm, change the metformin immediate-release (Met IR) reference arm from 1500 mg to 2000 mg per day, and modify eligibility criteria.

SECTION	ORIGINAL PROTOCOL	AMENDMENT 1 TEXT
Protocol Summary – Study Design; Section 3.1 – Design Description	During the lead-in period, subjects will be enrolled and will receive placebo twice daily (BID) (1× 600 mg matched placebo tablet in the morning and 1 x 600 mg matched placebo tablet in the evening).<br><br>During the treatment period, subjects will be randomly assigned to 1 of 7 treatment arms (Groups A, B, C, D, E, F, and G) in the ratio of 3:1:4:4:4:4:4, respectively.	During the lead-in period, subjects will be enrolled and will receive placebo once daily in the morning (qAM) (1× 600 mg matched placebo tablet) at the beginning of the morning meal.<br><br>During the treatment period, subjects will be randomly assigned to 1 of 7 treatment arms (Groups A, B, C, D, E, F, and G) in the ratio of 1:1:2:2:2:2:2, respectively
Protocol Summary – Table; Table 1: Randomized Treatment Arms and Dose Regimens (Protocol LCRM112)		*(Revised treatment arms, N, and dose regimens)*
Protocol Summary – overall study design figure; Figure 1 - Study Design		*(Revised treatment arms, N, and dose regimens)*
Protocol Summary – Study Population; Section 4.1 – Population to Be Studied	Approximately 552 males and non-pregnant females, at least 18 years of age, with T2DM (HbA1c 7.5% to 11.0%) and an estimated glomerular filtration rate (eGFR) of ≥60 mL/min/1.73 m^2 who are not taking metformin for at least 2 months prior to Screening are to be randomized in this study.	Approximately 552 males and non-pregnant females, at least 25 years of age, with T2DM (HbA1c 7.5% to 10.5%) and an estimated glomerular filtration rate (eGFR) of ≥60 mL/min/1.73 m^2 who are not taking metformin for at least 2 months prior to Screening are to be randomized in this study.
Protocol Summary – Study Medication; Section 5.1 – Formulation, Packaging and Storage	Met IR (Glucophage®): 500 mg metformin HCl immediate-release tablets	Met IR (Glucophage®): 1000 mg metformin HCl immediate-release tablets
Protocol Summary – Study Methods	*Not applicable*	Subjects randomized to receive single blind Met IR will titrate to a dose of 1000 mg Met IR BID (2000 mg Met IR per day in equal divided doses) on Day 8 from a starting dose of 1000 mg Met IR qAM. On Day 8, study site personnel are to make a scheduled telephone call to the subject to

SECTION	ORIGINAL PROTOCOL	AMENDMENT 1 TEXT
		remind the subject to increase the dose of treatment by adding the PM dose (Group G only). Treatment G will not be blinded from the study site personnel but the identity of the treatment will not be actively disclosed to subjects (single-blind).
	Subjects should administer their doses of study medication at approximately the same times each day throughout the study with food unless instructed otherwise and record the time and date of each dose of study medication in a dosing diary.	Subjects should administer their doses of study medication at the beginning of a meal and at approximately the same time each day throughout the study unless instructed otherwise by the investigator. Subjects will be instructed to record the time and date of each dose of study medication and complete a dosing diary.
Section 1.3.1 – Rationale for the Conduct of the Study	The purpose of the present study is to compare the glycemic effects of Met DR to placebo in subjects with T2DM over 16 weeks. The study is designed to evaluate several doses and dose regimens of Met DR compared to placebo. A single-blind reference treatment of 1500 mg Met IR will also be included	The purpose of the present study is to compare the glycemic effects of Met DR to placebo in subjects with T2DM at 16 weeks. The study is designed to evaluate several doses of Met DR compared to placebo. A single-blind reference treatment of 2000 mg Met IR per day administered as equal divided doses (1000 mg Met IR BID) will also be included.
Section 1.3.2 Dosage Selection	600 mg Met DR was previously been shown to have similar magnitude of effect as 1000 mg Met XR on fasting plasma glucose over 12 weeks.<br><br>The glycemic effect of a 1200 mg Met DR dose given either once daily in the morning (qAM) or as divided doses (i.e., 600 mg twice daily [BID]) will be evaluated.	600 mg Met DR has previously been shown to have similar magnitude of effect as 1000 mg Met XR on fasting plasma glucose over 12 weeks.<br><br>The glycemic effect of 1500 mg Met DR and a single blind active reference treatment of 2000 mg Met IR will be evaluated to explore the higher end of the dose range.<br><br>In the present study, subjects will receive total daily doses of 600, 900, 1200 or 1500 mg Met DR or 2000 mg Met IR. The single blind Met IR group will titrate to 2000 mg from a starting dose of 1000 mg (see Section 5.3). The recommended Met IR starting dose for patients with T2DM is 500 mg BID or 850 mg once a day, given

SECTION	ORIGINAL PROTOCOL	AMENDMENT 1 TEXT
		with meals, and the maximum recommended dose is 2550 mg/day given in divided doses BID or three times a day with meals. Thus, the target dosages selected for this study are within the recommended daily dose for patients diagnosed with T2DM.
Section 4.2 – Inclusion Criteria	Is at least 18 years old at Visit 1 (Screening).<br><br>Body mass index (BMI) 25.0 to 45.0 kg/m^2 (inclusive) at Visit 1 (Screening).<br><br>Has T2DM and an HbA1c of 7.5% to 11.0%, inclusive, at Visit 1 (Screening).<br><br>Either is not treated with or has been on a stable treatment regimen with any of the following medications for a minimum of 3 months prior to Visit 1 (Screening): Thiazolidinedione, sulfonylurea, dipeptidyl peptidase-4 inhibitors, glucagon-like peptide-1 receptor agonists, and alpha-glucosidase inhibitors	Is at least 25 years old at Visit 1 (Screening).<br><br>Body mass index (BMI) 20.0 to 45.0 kg/m^2 (inclusive) at Visit 1 (Screening).<br><br>Has T2DM and an HbA1c of 7.5% to 10.5%, inclusive, at Visit 1 (Screening).<br><br>Either is not treated with or has been on a stable treatment regimen with any of the following medications for a minimum of 3 months prior to Visit 1 (Screening): Thiazolidinedione, sulfonylurea, dipeptidyl peptidase-4 inhibitors, and alpha-glucosidase inhibitors
Section 4.3 – Exclusion Criteria	Has been treated, is currently being treated, or is expected to require or undergo treatment with any of the following excluded medications:<br>Sodium-glucose co-transporter 2 inhibitors pharmacological therapy within 2 months of Visit 1 (Screening)	Has been treated, is currently being treated, or is expected to require or undergo treatment with any of the following excluded medications: Glucagon-like peptide-1 receptor agonists or sodium-glucose co-transporter 2 inhibitors pharmacological therapy within 3 months of Visit 1 (Screening)
Section 4.4 – Subject Restrictions	Bring study medication to the clinic (except for Visit 2), and wait to take the morning dose after the fasting blood draws (except for Visit 8; study medication will not be administered on the day of this visit)	Bring study medication to the clinic (except for Visit 2), and wait to take the morning dose after the fasting blood draws (except for Visit 9; study medication will not be administered on the day of this visit)
Section 5.3 – Medication Administration Procedures and Route	Subjects are to be instructed to administer study medication orally with water, as intact tablets (swallowed whole, do not chew or crush).<br><br>*Not applicable*	Subjects are to be instructed to administer study medication orally with water, as intact tablets (swallowed whole, do not cut, chew or crush).<br><br>Refer to Table 1 for the dose regimen for each treatment group.

SECTION	ORIGINAL PROTOCOL	AMENDMENT 1 TEXT
	Groups A-F: During the first two weeks, take one tablet, once daily, at the beginning of your morning meal and one tablet, once daily, at the beginning of your evening meal, starting today until the day before your Visit 3 appointment. Starting after your Visit 3 appointment, take two tablets, once daily, at the beginning of your morning meal and two tablets, once daily at the beginning of your evening meal through the end of the study.<br><br>Group G: During the first two weeks, take one tablet, once daily, at the beginning of your morning meal and one tablet, once daily, at the beginning of your evening meal, starting today until the day before your Visit 3 appointment. Starting after your Visit 3 appointment, take two tablets, once daily, at the beginning of your morning meal and one tablet, once daily, at the beginning of your evening meal through the end of the study.<br><br>When you take your tablets, swallow them whole by mouth, with water, and do not crush or chew your tablets<br><br>*Not applicable*	Groups A-F: Take one tablet, once daily, at the beginning of your morning meal, starting today until the day before your Visit 3 appointment. Starting after your Visit 3 appointment, take two tablets, once daily, at the beginning of your morning meal through the end of the study.<br><br>Group G: Take one tablet, once daily, at the beginning of your morning meal, starting today until a week after your Visit 3 appointment. Starting a week after your Visit 3 appointment, take one tablet, once daily, at the beginning of your morning meal and one tablet, once daily, at the beginning of your evening meal through the end of the study.<br><br>When you take your tablets, swallow them whole, with water, and do not cut, crush or chew your tablets<br><br>Subjects randomized to receive single blind Met IR will titrate to a dose of 1000 mg Met IR BID (2000 mg Met IR per day in equal divided doses) on Day 8 from a starting dose of 1000 mg Met IR qAM. On Day 8, study site personnel are to make a scheduled telephone call to the subject to remind the subject to increase the dose of treatment (Group G only).
Section 5.4 – Randomization Schedule and Blinding Procedures	Treatments A, B, C, D, E, and F will be blinded to subjects, investigators, and the sponsor (double blind). As the tablets for Treatment G will be sourced from commercial supply, their appearance will be slightly different than Met DR and placebo tablets and the number of tablets	Groups A, B, C, D, E, and F will be blinded to subjects, investigators, and the sponsor (double blind). As the tablets for Group G will be sourced from commercial supply, their appearance will be different than Met DR and placebo tablets. The dosing regimen (1 tablet BID at the

SECTION	ORIGINAL PROTOCOL	AMENDMENT 1 TEXT
	administered per day will be different. Thus, Treatment G will not be blinded from the investigators but the identity of the treatment will not be disclosed to subjects (single-blind). Because all study medication will be packaged in blister packs and the identity of each treatment will not be disclosed to subjects, the difference in the appearance and number of tablets for Treatment G is not expected to result in breaking of the blind at the subject level.	target dose) will also be different for Group G than for the double blind groups (2 tablets qAM). Thus, Treatment G will not be blinded from the investigator or site staff, but the identity of the treatment will not be actively disclosed to subjects (single-blind). Because all study medication will be packaged in blister packs and the identity of each treatment will not be actively disclosed to subjects, the difference in the appearance of the tablets and the dosing regimen for Treatment G is not expected to result in breaking of the blind at the subject level.
Section 5.6 – Treatment Compliance	The time of the most recent dose of study medication prior to the fasting blood draws at Visits 4 through 9 must be documented on the source documents and electronic case report form (eCRF).	The time of the most recent dose of study medication prior to the plasma metformin blood draws at Visits 4 through 9 must be documented on the source documents and electronic case report form (eCRF).
Section 5.7 – Study Medication Dosing Diaries	Subjects are to record the time and date of each dose of study medication in a dosing diary. At Visit 2, subjects are to be instructed on how to use the dosing diary and will record their first dose of study medication administered at Visit 2 in the diary under supervision of study-site personnel.	Subjects are to record the time and date of each dose of study medication and complete a dosing diary. At Visit 2, subjects are to be instructed on how to use the dosing diary.
Section 6.1.3 – Visit 3 (Day 1): Randomization	*Not applicable*	Subjects randomized to receive single blind Met IR will titrate to a dose of 1000 mg Met IR BID (2000 mg Met IR per day in equal divided doses) on Day 8 from a starting dose of 1000 mg Met IR qAM. On Day 8, study site personnel are to make a scheduled telephone call to the subject to remind the subject to increase the dose of treatment (Group G only). Treatment G will not be blinded from the study site personnel but the identity of the treatment will not be actively disclosed to subjects (single-blind).
Section 6.1.7 – Early Termination	Subjects who withdraw prior to completion of Visit 8 are to complete early termination procedures in a timely manner.	Subjects who withdraw prior to completion of Visit 9 are to complete early termination procedures in a timely manner.

SECTION	ORIGINAL PROTOCOL	AMENDMENT 1 TEXT
Section 14.7 – Interim Analysis	An interim analysis may be conducted once all subjects have either completed 4 weeks of treatment or discontinued the study prior to completing 4 weeks of treatment. The analysis will include the changes in fasting plasma glucose and HbA1c from baseline to Week 4. Results from the analysis will be used to confirm dose selection for subsequent studies of Met DR. Results from the analysis will remain confidential from personnel actively involved in study conduct so that the blind can be maintained until the end of the treatment period. Procedures for controlling the Type I error rate for the interim and final analyses will be specified in the SAP.	An interim analysis may be conducted once all subjects have either completed 4 weeks of treatment or discontinued the study prior to completing 4 weeks of treatment. The analysis, if conducted, will include the changes in fasting plasma glucose and HbA1c from baseline to Week 4. Results from the analysis will be used for planning purposes only. Results from the analysis will remain confidential from personnel actively involved in study conduct so that the blind can be maintained until the end of the treatment period. Procedures for controlling the Type I error rate for the interim and final analyses will be specified in the SAP.
Section 10.1.1 – Adverse Events	ALL ADVERSE EVENTS THAT OCCUR AFTER THE SUBJECT HAS SIGNED THE ICF WILL BE RECORDED ON SOURCE DOCUMENTS. ADVERSE EVENTS FOR SUBJECTS WHO ARE RANDOMIZED IN THE STUDY WILL BE ENTERED ON SOURCE DOCUMENTS AND/OR eCRFs.	ALL ADVERSE EVENTS THAT OCCUR AFTER THE SUBJECT HAS SIGNED THE ICF WILL BE RECORDED ON SOURCE DOCUMENTS. ADVERSE EVENTS FOR SUBJECTS WHO ARE ENROLLED IN THE STUDY WILL BE ENTERED ON SOURCE DOCUMENTS AND/OR eCRFs.
Section 12.1 - Unblinding	As described in Section 5.4, Treatment G will not be blinded from the investigator or site personnel but the identity of the treatment will not be disclosed to subjects (single-blind).	As described in Section 5.4, Treatment G will not be blinded from the investigator or site personnel but the identity of the treatment will not be actively disclosed to subjects (single-blind).
Appendix 1: Study Plan (Protocol LCRM112)	*Not applicable*	*Combined rows for Medical History, Height and BMI and HbA1c, Fasting Plasma Glucose, Lactate, respectively*<br><br>*Added footnote* [5] Subjects randomized to Group G will titrate to a 1 tablet BID dose on Day 8 from a starting dose of 1 tablet qAM. On Day 8, study site personnel are to make a scheduled telephone call to the subject to remind the subject to increase the dose of treatment by adding the PM dose (Group G only).

Appendix 2. Protocol Amendment 2: Summary of Changes

Date: 08 October 2015

Amendment Purpose: The purpose of this amendment is to modify the screening visit structure for subjects who wash out of prior metformin therapy and to clarify the following items: the timing of Visits 4 and 6 relative to the evening dose of Group G study medication, instructions in case of missed doses of study medication, exclusion of individuals with potential prior exposure to metformin delayed-release (Met DR), eligibility requirements and withdrawal procedures for subjects enrolled into lead-in, and permitted hyperglycemia rescue medications.

SECTION	AMENDMENT 1 TEXT	AMENDMENT 2 TEXT
Protocol Summary – Study Design; Section 3.1 – Design Description, Figure 1	*Not applicable*<br><br>[2] Visit 2 (Week -2) will occur within 4 weeks following Visit 1 (Screening).	*Added Visit WS to figure*<br><br>[2] Subjects may wash out of prior metformin therapy after initiation of screening procedures at Visit WS (Washout Screening) before Visit 1 (Screening) if appropriate based on the investigator's clinical judgment. Such subjects may qualify for study enrollment at Visit 1 (Screening) after a 60- to 75-day metformin wash out period. Visit 2 (Week -2) will occur within 2 weeks following Visit 1 (Screening).
Protocol Summary – Study Design; Section 3.1 – Design Description	There will be two placebo arms (Groups A and B), four Met DR arms (Groups C, D, E, and F) and one Met IR arm (Group G). Groups A, B, C, D, E, and F will be double blinded. Group G will be single blinded.	There will be 2 placebo arms (Groups A and B), 4 Met DR arms (Groups C, D, E, and F) and 1 Met IR arm (Group G). Groups A, B, C, D, E, and F will be double-blinded. Group G will be single-blinded.
Protocol Summary – Visit Structure; Section 3.2 – Visit Structure	*Not applicable*<br><br>Visit 2 (Week -2) will occur within 4 weeks following Visit 1 (Screening).	Subjects may wash out of prior metformin therapy after initiation of screening procedures at Visit WS (Washout Screening) before Visit 1 (Screening), if appropriate based on the investigator's clinical judgment. Such subjects may qualify for study enrollment at Visit 1 (Screening) after a 60- to 75-day metformin wash out period. Visit 1 procedures may be conducted over >1 day.<br><br>Visit 2 (Week -2) will occur within 2 weeks following Visit 1 (Screening).

SECTION	AMENDMENT 1 TEXT	AMENDMENT 2 TEXT
	Visit 3 (Day 1) will occur two weeks (±3 days) following Visit 2 (Week -2).<br><br>Visits 4 and 6 (Weeks 2 and 6) are to occur at least 4 hours after subjects administer study medication in the morning with food.<br><br>Required assessments by visit are presented in Appendix 1.	Visit 3 (Day 1) will occur 2 weeks (±3 days) following Visit 2 (Week -2).<br><br>Visits 4 and 6 (Weeks 2 and 6) are to occur at least 4 hours after subjects administer the morning dose of study medication and before subjects who are randomized to single-blind Group G administer the evening dose of study medication.<br><br>*Deleted from summary; not applicable for Section 3.2.*
Protocol Summary – Study Duration; Section 3.3 – Study Duration	The total study duration is approximately 18 to 22 weeks depending on the number of days between the start of Visit 1 and Visit 2. The study will include a screening period of up to 4 weeks, a 2-week lead-in period, and a 16-week treatment period.	The total study duration is approximately 18 to 32 weeks depending on the number of days between Visit WS (if applicable), Visit 1, and Visit 2. The study will include a screening period of up to 3 months, a 2-week lead-in period, and a 16-week treatment period.
Protocol Summary – Study Population	Subjects may wash out of prior metformin therapy after initiation of screening procedures if appropriate per the clinical judgment of the investigator; such subjects may re-screen for the study with a new screening number after the wash out period.	Subjects may wash out of prior metformin therapy after initiation of screening procedures at Visit WS (Washout Screening) if appropriate based on the investigator's clinical judgment and guided by the following criteria: HbA1c 7.0 to 9.5% (inclusive) prior to initiation of wash out.
Protocol Summary – Study Medication; Section 5.1 – Formulation, Packaging, and Storage	Met DR (EFB0082): 600 mg HCl delayed-release tablets	Met DR (EFB0082): 600 mg metformin hydrochloride (HCl) delayed-release tablets
Protocol Summary – Study Medication	All active Met DR study medication tablet strengths (600 and 900 mg tablets) will have matching placebo tablets that are identical in size and appearance to maintain the treatment blind. Met IR is included as an unblinded active reference arm and will not have matching placebo tablets.	*Deleted*
Protocol Summary – Study Methods	Subjects randomized to receive single blind Met IR will titrate to a dose of 1000 mg Met IR BID (2000 mg Met IR per day in equal divided doses) on Day 8 from a starting dose of 1000 mg Met IR qAM	Subjects randomized to receive single-blind Met IR will titrate to a dose of 1000 mg Met IR twice daily (BID) (2000 mg Met IR per day in equal divided doses) on Day 8 from a starting dose of 1000 mg Met IR qAM.

SECTION	AMENDMENT 1 TEXT	AMENDMENT 2 TEXT
Protocol Summary – Study Methods; Section 6.1.6 – Visit 4 (Week 2) and Visit 6 (Week 6): Randomized Treatment Period	*Not applicable*	Subjects randomized to Group G are to wait to take the evening dose of study medication after the plasma metformin blood collection.
Section 1.3.2 – Dosage Selection	Based on Phase 2 studies, 1200 mg Met DR is expected to be well tolerated and provide efficacy results similar to 1500 mg Met IR with lower plasma exposure.<br><br>The glycemic effect of 1500 mg Met DR and a single blind active reference treatment of 2000 mg Met IR will be evaluated to explore the higher end of the dose range.	Based on Phase 2 studies, 1200 mg Met DR is expected to be well tolerated and provide efficacy results similar to 2000 mg Met IR with lower plasma exposure.<br><br>The glycemic effect of 1500 mg Met DR will be evaluated to explore the higher end of the dose range. 2000 mg Met IR will be included as a single-blind active reference treatment.
Section 4.1 – Study Population	Approximately 552 males and non-pregnant females, at least 25 years of age, with T2DM (HbA1c 7.5% to 10.5%) and an eGFR of ≥60 mL/min/1.73 m^2 who are not taking metformin for at least 2 months prior to Screening are to be randomized in this study.<br><br>*Not applicable*	Approximately 552 males and non-pregnant females, at least 25 years of age, with T2DM (HbA1c 7.5% to 10.5%) and an estimated glomerular filtration rate (eGFR) of ≥60 mL/min/1.73 m^2 who are not taking metformin for at least 2 months prior to Screening are to be randomized in this study.<br><br>Such subjects may qualify for study enrollment at Visit 1 (Screening) after a 60- to 75-day metformin wash out period; for such subjects, eligibility for the study based on thresholds outlined in the inclusion and exclusion criteria (Sections 4.2 and 4.3) is to be determined based on assessments at the time of Visit 1 (Screening).
Section 4.3 – Exclusion Criteria	Exclusion Criteria 8.c. Glucagon-like peptide-1 receptor agonists or sodium-glucose co-transporter 2 inhibitors pharmacological therapy within 3 months of Visit 1 (Screening)<br><br>*Not applicable*	Exclusion Criteria 8.c. Glucagon-like peptide-1 receptor agonists or sodium-glucose co-transporter 2 inhibitors within 3 months of Visit 1 (Screening)<br><br>Exclusion Criteria 8.j. Met DR or double-blind matching placebo for Met DR at any time prior to Visit 1 (Screening)
Section 4.4 – Subject Restrictions	*Not applicable*	Wait to take the evening dose of study medication after the plasma metformin blood collection (Group G only)
Section 5.6 – Treatment Compliance	The time of the most recent dose of study medication prior to the	The time of the most recent dose of study medication prior to the

SECTION	AMENDMENT 1 TEXT	AMENDMENT 2 TEXT
	plasma metformin blood draws at Visits 4 through 9 must be documented on the source documents and electronic case report form (eCRF).<br><br>Subjects who miss a dose should be instructed to take their next dose as originally scheduled.	plasma metformin blood draws at Visits 3 through 9 must be documented on the source documents and electronic case report form (eCRF).<br><br>Subjects who miss a dose should be instructed to take the dose only if less than half the time has passed until the next scheduled dose. If more than half the time has passed until the next scheduled dose, the subject should be instructed not to take the missed dose (i.e., do not double dose). Subjects should be reminded of the importance of dosing at approximately the same time each day as instructed.
Section 6.1 – Study Procedures	*Not applicable*	*Section numbers adjusted per addition of Section 6.1.1 – Visit WS (Washout Screening)*
Section 6.1.1 – Visit WS: Washout Screening Procedures	*Not applicable*	*Addition of Section 6.1.1 – Visit WS (Washout Screening)*
Section 6.1.2 – Visit 1: Screening Procedures	Obtain signatures for the Informed Consent Form (ICF) and Health Insurance Portability and Accountability Act (HIPAA) Authorization forms prior to performing any protocol related procedures<br><br>Record complete medical history, including menopausal status for females<br><br>Measure height and calculate BMI<br><br>βhCG for female subjects of childbearing potential (prior to the first blood draw)<br><br>Subjects may wash out of prior metformin therapy after initiation of screening procedures if appropriate per the clinical judgment of the investigator; such subjects may re-screen for the study with a new screening number after the wash out period.	Obtain signatures for the ICF and HIPAA Authorization forms prior to performing any protocol related procedures (if not obtained at Visit WS)<br><br>Record complete medical history, including menopausal status for females (if not obtained at Visit WS)<br><br>Measure height (if not obtained at Visit WS) and calculate BMI<br><br>βhCG for female subjects of childbearing potential<br><br>*Deleted*
Section 6.1.3 – Visit 2 (Week -2) Enrollment, Lead-in Period	Visit 2 (Week -2) will occur within 4 weeks following Visit 1 (Screening) between 0600 and 1000 hours if possible.	Visit 2 (Week -2) will occur within 2 weeks following Visit 1 (Screening), between 0600 and 1000 hours if possible.

SECTION	AMENDMENT 1 TEXT	AMENDMENT 2 TEXT
	βhCG for female subjects of childbearing potential (prior to the first blood draw)<br><br>*Not applicable*	βhCG for female subjects of childbearing potential<br><br>Individuals are to be withdrawn from lead-in if results of any clinical laboratory test fails eligibility thresholds outlined in the inclusion and exclusion criteria (Sections 4.2 and 4.3). Subjects who discontinue from the study during the lead-in period are not required to return to the clinic for an Early Termination visit.
Section 6.1.8 – Early Termination	Subjects who withdraw from the study prior to completion of Visit 8 are to complete early termination procedures in a timely manner.	Subjects who are randomized at Visit 3 (Day 1) and who withdraw from the study prior to completion of Visit 9 are to complete early termination procedures in a timely manner.
Section 7.4 - Hyperglycemia	Rescue medications may include antidiabetic medications that are listed as exclusionary in Section 4.3.	Rescue medications may include dose adjustments to current antidiabetic medications and/or addition of thiazolidinediones, sulfonylureas, dipeptidyl peptidase-4 inhibitors, alpha-glucosidase inhibitors, insulin, and/or glucagon-like peptide-1 receptor agonists. Prescribed metformin and sodium-glucose co-transporter 2 inhibitors are not permitted as rescue medications.
Section 10.3.2 – Physical Examinations	All body systems listed in the source document and eCRF will be evaluated.	All body systems listed in the source document will be evaluated.
Section 11 – Blood Volume	The maximum amount of blood to be drawn during the entire study is expected to be approximately 150 mL for each subject and the maximum to be drawn at a single visit will be approximately 25 mL.	The maximum amount of blood to be drawn during the entire study is expected to be approximately 170 mL for each subject and the maximum to be drawn at a single visit will be approximately 25 mL.
Appendix 2: Study Plan (Protocol LCRM112)	*Not applicable*<br><br>All visits subsequent to Visit 2 will occur at 2- or 4-week intervals (±3 days relative to Visit 3 [Day 1]).	*Added Visit WS (Washout Screening); minor editorial revisions*<br><br>All visits subsequent to Visit 3 will occur at 2- or 4-week intervals (±3 days relative to Visit 3 [Day 1]).
All Sections	*Not applicable*	*Minor editorial revisions were applied throughout*

Appendix 3. Protocol Amendment 3: Summary of Changes

Date: 30 November 2015

Amendment Purpose: The purpose of this amendment is several-fold and includes: further clarifying exclusion of subjects who plan to use any drug treatment that affects gastric pH (such as H2-receptor antagonists and proton pump inhibitors or chronic antacid use), updating the name and contact information of the medical monitor, clarifying the HbA1c recommendation regarding subjects who will be washed off prior metformin, excluding subjects with significant blood loss prior to study entry or who are planning to donate blood during the study, and excluding subjects with a sustained fasting plasma glucose value >270 mg/dL prior to randomization into the study.

SECTION	AMENDMENT 2 TEXT	AMENDMENT 3 TEXT
Title Page	Bernard E. Ilson, MD, FACP<br>Vice President, Medical Affairs<br>Medpace<br>Telephone: (513) 579-9911	Phillippa Miranda, MD<br>Medical Director<br>Medpace<br>Telephone: (513) 579-9911 ext. 2275
Approval Page	____________________<br>Bernard E. Ilson, MD,<br>FACP<br><br>Date<br>Vice President, Medical Affairs<br>Medpace	____________________<br>Phillippa Miranda, MD<br>Medical Director<br>Medpace
Synopsis - Study Population<br>Section 4.1 - Population to be Studied<br>Section 6.1.1 - Visit WS: Washout Screening Procedures	Subjects may wash out of prior metformin therapy after initiation of screening procedures at Visit WS (Washout Screening) if appropriate based on the investigator's clinical judgment and guided by the following criteria: HbA1c 7.0 to 9.5% (inclusive) prior to initiation of wash out.	Subjects may wash out of prior metformin therapy after initiation of screening procedures at Visit WS (Washout Screening) if appropriate based on the investigator's clinical judgment. As subjects are required to have HbA1c 7.5 to 10.5% (inclusive) at Visit 1 and Visit 2, it is recommended (but not required) that subjects have HbA1c 7.0 to 9.5% (inclusive) prior to initiation of the wash out period.
Section 4.3 - Exclusion Criteria	Exclusion criteria 8.f. Chronic or frequent use, in the judgment of the investigator, of any drug treatment that affects gastric pH (prescription or over-the-counter), including proton pump inhibitors or any antacids or medications such as Rolaids® or Pepcid® within 1 month of Visit 1 (Screening)	Exclusion criteria 8.f. Planned use of any drug treatment that affects gastric pH (prescription or over-the-counter), such as H2-receptor antagonists and proton pump inhibitors, after Visit 2 (Week -2), or planned chronic use of any antacids (i.e., more than twice per week) after Visit 2 (Week -2).
Section 4.3 - Exclusion Criteria	Exclusion criterion 10. Has donated blood within 3 months of the date of the first dose of randomized study medication, or is planning to donate blood during the study.	Exclusion criterion 10. Had a blood transfusion or experienced significant blood loss (i.e., >500 mL), including loss due to blood donation, within 2 months prior to

SECTION	AMENDMENT 2 TEXT	AMENDMENT 3 TEXT
		Visit 1 (Screening), or is planning to donate blood during the study.
Section 4.3 - Exclusion Criteria	*Not applicable*	Exclusion criterion 13. Has a fasting plasma glucose value >270 mg/dL at Visit 1 (Screening), Visit 2 (Week -2), and an unscheduled visit to be completed within 1 week following Visit 2. The unscheduled visit is to be completed only for subjects with a fasting plasma glucose value >270 mg/dL at Visit 1 and Visit 2.
Section 4.4 - Subject Restrictions	*Not applicable*	Do not take any antacids (e.g., Rolaids® and TUMS®) within 2 hours of study medication administration
Section 6.1.3 - Visit 2 (Week -2): Enrollment, Lead-in Period	*Not applicable*	Subjects who have a fasting plasma glucose value >270 mg/dL at Visit 1 (Screening) and Visit 2 (Week -2) may have the test repeated at an unscheduled visit within 1 week following Visit 2.

Appendix 4. Protocol Amendment 4: Summary of Changes

Date: 18 January 2016

Amendment Purpose: The purpose of this amendment is to adjust the timing of study procedures relevant to Visit WS, to clarify the restriction on intrapulmonary steroids, to further clarify that subjects must meet laboratory eligibility requirements both at Visit 1 and Visit 2, and to provide guidance on retesting permitted for Visit 2. The addition of a criterion for acceptable change in HbA1c at Visit 2 relative to Visit 1 is intended to account for potential variability in HbA1c assay results.

SECTION	AMENDMENT 3 TEXT	AMENDMENT 4 TEXT
Summary – Study Population Section 4.1 – Population to be Studied	Approximately 552 males and non-pregnant females, at least 25 years of age, with T2DM (HbA1c 7.5% to 10.5%) and an estimated glomerular filtration rate (eGFR) of ≥60 mL/min/1.73 m^2 who are not taking metformin for at least 2 months prior to Screening are to be randomized in this study.	Approximately 552 males and non-pregnant females, at least 25 years of age, with T2DM (HbA1c 7.5% to 10.5%) and an estimated glomerular filtration rate (eGFR) of ≥60 mL/min/1.73 m^2 who are not taking metformin for at least 2 months prior to Screening (Visit 1) are to be randomized in this study.
Section 4.1 – Population to be Studied	N/A	Subjects must meet laboratory eligibility requirements both at Visit 1 and Visit 2; see Sections 6.1.2 and 6.1.3 for further guidance on retesting.
Section 4.2 – Inclusion Criteria	Inclusion Criterion 5. Has T2DM and an HbA1c of 7.5% to 10.5%, inclusive, at Visit 1 (Screening).	Inclusion Criterion 5. Has T2DM and an HbA1c of 7.5% to 10.5%, inclusive.
Section 4.3 – Exclusion Criteria	Exclusion Criterion 5. A clinical laboratory test (clinical chemistry, hematology, or urinalysis) abnormality, other than that related to T2DM, judged by the investigator to be clinically significant at Visit 1 (Screening).	Exclusion Criterion 5. A clinical laboratory test (clinical chemistry, hematology, or urinalysis) abnormality, other than that related to T2DM, judged by the investigator to be clinically significant.
Section 4.3 – Exclusion Criteria	Exclusion Criterion 8e. Systemic corticosteroids by oral, intravenous, or intramuscular route; or potent, inhaled, or intrapulmonary (including ADVAIR®) steroids known to have a high rate of systemic absorption within 3 months of Visit 1 (Screening)	Exclusion Criterion 8e. Systemic corticosteroids by oral, intravenous, or intramuscular route; or potent, inhaled, or intrapulmonary steroids known to have a high rate of systemic absorption within 3 months of Visit 1 (Screening)
Section 6.1.1 – Visit WS: Washout Screening Procedures	Individuals may re-qualify within 2 weeks of Visit WS following an abnormal test result by having that test repeated once with acceptable	Individuals may re-qualify within 5 days of Visit WS following an abnormal test result by having that test repeated once with acceptable

	results as judged by the investigator and medical monitor (or designees).	results as judged by the investigator and medical monitor (or designees).
Section 6.1.1 – Visit WS: Washout Screening Procedures	Those who qualify will be instructed to discontinue prescribed metformin and will be eligible to return to the study site to be screened at Visit 1 (Screening) after completion of the 60- to 75-day metformin wash out period.	Those who qualify will be instructed to discontinue prescribed metformin within 7 days of Visit WS and will be eligible to return to the study site to be screened at Visit 1 (Screening) after completion of the 60- to 75-day metformin wash out period.
Section 6.1.3 – Visit 2 (Week -2): Enrollment, Lead-in Period	Individuals are to be withdrawn from lead-in if results of any clinical laboratory test fails eligibility thresholds outlined in the inclusion and exclusion criteria (Sections 4.2 and 4.3).<br>• Subjects who have a fasting plasma glucose value >270 mg/dL at Visit 1 (Screening) and Visit 2 (Week -2) may have the test repeated at an unscheduled visit within 1 week following Visit 2.	Individuals are to be withdrawn from lead-in if results of any clinical laboratory test fails eligibility thresholds outlined in the inclusion and exclusion criteria (Sections 4.2 and 4.3), unless relevant criteria defined below are met.<br>• Subjects who have a fasting plasma glucose value >270 mg/dL at Visit 1 (Screening) and Visit 2 (Week -2) may have the test repeated at an unscheduled visit within 1 week following Visit 2. The repeat fasting plasma glucose value must be ≤ 270 mg/dL for the subject to continue in the study.<br>• If the result of the HbA1c test at Visit 2 fails eligibility thresholds outlined in inclusion criterion 5 (HbA1c of 7.5% to 10.5% inclusive) then both of the below criteria must be met for the subject to continue in the study.<br>o The value increased or decreased by ≤ 0.3% relative to a qualifying Visit 1 value, and<br>o The value is assessed as not a clinically significant change from the Visit 1 value by the investigator in consultation with the medical monitor (or designees)<br>• If the result of the eGFR test at Visit 2 fails the eligibility threshold outlined in inclusion criterion 6 (eGFR of ≥60 mL/min/1.73 m^2), the subject is to be withdrawn unless

		the value is assessed as not a clinically significant change from the qualifying Visit 1 value by the investigator in consultation with the medical monitor (or designees).<br>• If the result of the liver function tests at Visit 2 fails eligibility thresholds outlined in exclusion criteria 6 (ALT >2.5 x ULN, AST >2.5 x ULN, and/or bilirubin results >1.5 x ULN), the subject is to be withdrawn unless the value is assessed as not a clinically significant change from the qualifying Visit 1 value by the investigator in consultation with the medical monitor (or designees).<br>For all clinically significant clinical laboratory test abnormalities, individuals may re-qualify for randomization by having the test repeated once at an unscheduled visit within 7 days following Visit 2 if appropriate based on the investigator's clinical judgment in consultation with the medical monitor (or designees) with acceptable results as judged by the investigator and medical monitor (or designees).
Section 6.1.4 – Visit 3 (Day 1): Randomization	N/A	The investigator (or qualified designee) is to confirm based on adequate compliance with study procedures whether the participant continues to be suitable for randomization.
Appendix 4: Study Plan (Protocol LCRM112)	N/A<br><br>*Footnotes [5] and [6]*	[5] Subjects must meet laboratory eligibility requirements both at Visit 1 and Visit 2; see Sections 6.1.2 and 6.1.3 for further guidance on retesting.<br><br>*Adjusted numbering to [6] and [7]*

Appendix 5. Study Plan (Protocol LCRM112)

Evaluation	Visit WS[1] (Washout Screening)	Visit 1 (Screening)	Visit 2 (Week -2)	Visit 3 (Day 1)	Visit 4, 6 (Weeks 2, 6)	Visits 5, 7, 8 (Weeks 4, 8, 12)	Study Term. Visit 9 (Week 16)	Early Term.
Fast (≥10 Hours) [2]	X	X	X	X		X	X	X
Informed Consent/HIPAA	X	X [3]						
Medical History, Height, and BMI	X	X [3]						
Vital Signs	X	X	X	X	X	X	X	X
Abbreviated Physical Examination	X							
Physical Examination		X					X	X
12-Lead ECG	X	X					X	X
HbA1c, Fasting Plasma Glucose, Lactate	X	X	X	X		X	X	X
Plasma Metformin				X	X	X	X	X
Chemistry	X	X	X	X		X	X	X
Hematology, Urinalysis	X	X	X	X			X	X
Vitamin B_{12}				X			X	X
Urine Pregnancy Test (Females of Childbearing Potential)	X	X	X	X			X	X
Concomitant Medications Review	X [4]	X [4]	X	X	X	X	X	X
Adverse Events and Standard of Care Review	X	X	X	X	X	X	X	X
Study Eligibility Assessment	X	X [5]	X	X				
Randomization				X				
Dispense Study Medication			X	X		X		
Study Medication Administration [6]			X	X [7]		X		
Dosing Diary Training			X					
Collect Unused Study Medication				X		X	X	X
Study Medication Compliance, Dosing Diary Review				X	X	X	X	X

Appendix 5. Study Plan (Protocol LCRM112) - Continued

BMI = body mass index; ECG = electrocardiogram; HbA1c = hemoglobin-specific A1c fraction; HIPAA = Health Insurance Portability and Accountability Act; term = termination.

[1] Subjects may wash out of prior metformin therapy after initiation of screening procedures at Visit WS if appropriate based on the investigator's clinical judgment. Such subjects may qualify for study enrollment at Visit 1 (Screening) after a 60- to 75-day metformin wash out period; for such subjects, eligibility for the study based on thresholds outlined in the inclusion and exclusion criteria (Sections 4.2 and 4.3) is to be determined based on assessments at the time of Visit 1 (Screening).

[2] Prior to the first blood draw of the visit.

[3] Obtain if not previously obtained at Visit WS.

[4] All prior medications within the last 3 months relative to Visit 1 (Screening) are to be recorded.

[5] Subjects must meet laboratory eligibility requirements both at Visit 1 and Visit 2; see Sections 6.1.2 and 6.1.3 for further guidance on retesting.

[6] Study medication will be administered after the fasting blood draws are completed.

[7] Subjects randomized to Group G will titrate to a 1 tablet BID dose on Day 8 from a starting dose of 1 tablet qAM. On Day 8, study site personnel are to make a scheduled telephone call to the subject to remind the subject to increase the dose of treatment by adding the PM dose (Group G only).

Note: Visit 2 (Week -2) will occur within 2 weeks following Visit 1 (Screening). Visit 3 (Day 1) will occur two weeks (±3 days) after Visit 2 (Week -2). All visits subsequent to Visit 3 will occur at 2- or 4-week intervals (±3 days relative to Visit 3 [Day 1]).
